# Brasilterpenes A–E, Bergamotane Sesquiterpenoid Derivatives with Hypoglycemic Activity from the Deep Sea-Derived Fungus *Paraconiothyrium brasiliense* HDN15-135

**DOI:** 10.3390/md20050338

**Published:** 2022-05-23

**Authors:** Wenxue Wang, Yeqin Shi, Yuanyuan Liu, Yundong Zhang, Jiajin Wu, Guojian Zhang, Qian Che, Tianjiao Zhu, Mingyu Li, Dehai Li

**Affiliations:** 1 Key Laboratory of Marine Drugs, Chinese Ministry of Education, School of Medicine and Pharmacy, Ocean University of China, Qingdao 266003, China; bx_wwx@163.com (W.W.); lyy19970310@163.com (Y.L.); zhangyundong0908@163.com (Y.Z.); wjj981123@163.com (J.W.); zhangguojian@ouc.edu.cn (G.Z.); cheqian064@ouc.edu.cn (Q.C.); zhutj@ouc.edu.cn (T.Z.); 2 Fujian Provincial Key Laboratory of Innovative Drug Target Research, School of Pharmaceutical Science, Xiamen University, Xiamen 361102, China; syq001235@163.com; 3 Laboratory for Marine Drugs and Bioproducts of Qingdao National Laboratory for Marine Science and Technology, Qingdao 266237, China

**Keywords:** marine-derived fungus, ascomycete fungus *Paraconiothyrium brasiliense*, bergamotane sesquiterpenoids, hypoglycemic activity

## Abstract

Five bergamotane sesquiterpenoid derivatives, brasilterpenes A–E (**1**–**5**), bearing an unreported spiral 6/4/5 tricyclic ring system, were isolated from the deep sea-derived ascomycete fungus *Paraconiothyrium brasiliense* HDN15-135. Their structures, including absolute configurations, were established by extensive spectroscopic methods complemented by single-crystal X-ray diffraction analyses, electronic circular dichroism (ECD), and density-functional theory (DFT) calculations of nuclear magnetic resonance (NMR) data including DP4+ analysis. The hypoglycemic activity of these compounds was assessed using a diabetic zebrafish model. Brasilterpenes A (**1**) and C (**3**) significantly reduced free blood glucose in hyperglycemic zebrafish in vivo by improving insulin sensitivity and suppressing gluconeogenesis. Moreover, the hypoglycemic activity of compound **3** was comparable to the positive control, anti-diabetes drug rosiglitazone. These results suggested brasilterpene C (**3**) had promising anti-diabetes potential.

## 1. Introduction

Diabetes is a chronic metabolic condition marked by high blood glucose (or blood sugar) levels, which can cause catastrophic damage to the heart, blood vessels, eyes, kidneys, and nerves over time [1]. Over the last few decades, the prevalence of diabetes has progressively increased, and a global consensus has been made to halt the rise in diabetes and obesity [2]. Hence, new therapeutic techniques and medications are urgently required. Marine-derived sesquiterpenoids have attracted much attention due to structural diversity and biological activities [3,4,5,6]. According to studies, they have hypoglycemic activity and can trigger pancreatic β-cell regeneration [7,8], suggesting that sesquiterpenoids could be the leading compounds for anti-diabetes drug development.

**Figure 1:**
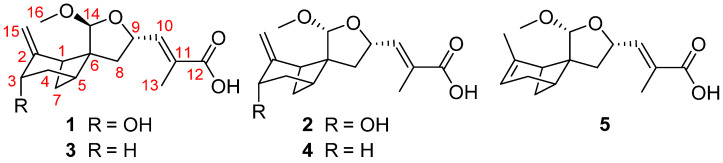
Structures of compounds **1**–**5**.

In our ongoing investigations of bioactive compounds from marine-derived fungi [9,10], an ascomycete fungal strain, *Paraconiothyrium brasiliense* HDN15-135, isolated from the deep-sea sediment (−3824 m) from the Indian Ocean, attracted our attention. Several chemical studies of the genus *Paraconiothyrium* have revealed that they can produce various types of active terpenoids, for example, hawaiinolide A (a cytotoxic cleistanthane diterpenoid) [11], brasilamides B–D (three bergamotane sesquiterpenoids with HIV-1 inhibitory activity) [12], brasilamide E (a cytotoxic bisabolane sesquiterpenoid) [13], and paraconiothin C (an eremophilane sesquiterpenoid with liver X receptor inhibitory activity) [14]. A variety of culture conditions were used to explore the metabolic potential of HDN15-135. It can produce a series of terpene-like terminal absorption compounds in the static culture of 2^#^ media (Figure S1). Further exploration of the components led to the isolation of five bergamotane sesquiterpenoid derivatives, named brasilterpenes A–E (**1**–**5**) (Figure 1).

Since the first discovery of *β*-bergamotene in 1963, about 33 bergamotane type sesquiterpenoids have been isolated, which were classified into five core ring systems, bicyclo[3.1.1]heptan [15,16,17,18,19,20,21,22,23], tricyclo[4.3.0.0^4,7^]nonane [12,20,24,25,26,27], tricyclo[3.3.1.0^2,7^]nonane [12,24], 6/4/5/5 tetracyclic skeleton [20,23,28,29,30], and 5/6/4/5 tetracyclic skeleton [23] (Figure S2). Unusually, compounds **1**–**5** possessed an unreported 6/4/5 tricyclic ring system. Among them, compounds **1** and **3** with a special *S* configuration at C-14 could reduce free blood glucose in hyperglycemic zebrafish by improving insulin sensitivity and suppressing gluconeogenesis in vivo. Details of the isolation, structural elucidation, and hypoglycemic activity of compounds **1**–**5** are described herein. A plausible biosynthetic pathway and structure–activity relationships for these isolates are also proposed.

## 2. Results and Discussion

The fungal strain *P. brasiliense* HDN15-135 was inoculated (40 L) under static condition at room temperature for 30 days. The ethyl acetate extract (38 g) was fractionated by extensive column chromatography (silica gel, ODS, Sephadex LH-20 and semi-preparative C18 HPLC chromatography) to afford five bergamotane-sesquiterpenoid derivatives (**1**–**5**) (Figure 1).

Brasilterpene A (**1**), was isolated as colorless crystals. Its molecular formula was determined as C_16_H_22_O_5_ based on the deprotonated molecule peak at *m*/*z* 293.1393 [M − H]^−^ obtained by HRESIMS, which requires six degrees of unsaturation. The IR spectrum showed absorption bands for a carbonyl group at 1699 cm^−1^ and hydroxy groups at 3421 cm^−1^. The 1D NMR data of **1** (Table 1) revealed 22 protons and 16 carbon signals. Analysis of the ^1^H NMR along with the HSQC spectra indicated resonances of one methyl group (*δ*_H_ 1.77), one methoxy group (*δ*_H_ 3.20), three aliphatic methylenes (*δ*_H_ 1.55, 1.64, 1.97, 2.17, 2.38, and 2.42), one olefinic methylene (*δ*_H_ 4.80, 5.03), two aliphatic methines (*δ*_H_ 2.23, 2.61), two oxygen-linked methines (*δ*_H_ 4.28, 4.81), one ketal methine (*δ*_H_ 4.37), one olefinic methine (*δ*_H_ 6.60), and two active hydrogens (*δ*_H_ 4.90, 12.40). Furthermore, analysis of the ^13^C NMR spectrum revealed two double bonds (*δ*_C_ 153.8, 142.1, 128.3, and 110.7), one ketal carbon (*δ*_C_ 105.7), and one carbonyl carbon (*δ*_C_ 168.6). These groups accounted for three out of the six degrees of unsaturation, requiring three additional rings in **1**.

**Figure 2:**
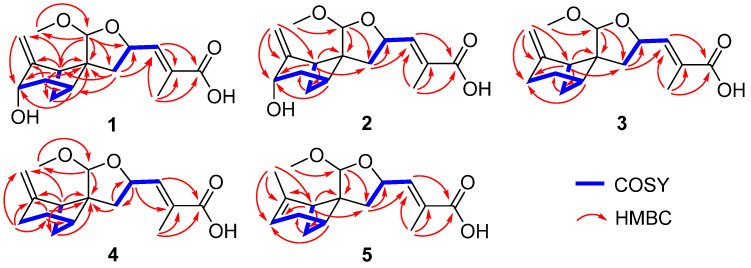
Key HMBC and COSY correlations of compounds **1**–**5**.

The 2-methylenebicyclo[3.1.1]heptane moiety in **1** was established by the COSY correlations of H-3/H-4/H-5/H-7/H-1, and the HMBC correlations from H-1 to C-2/C-3/C-6/C-7/C-15 (Figure 2), identical to that of massarinolin C [20]. The characteristic HMBC correlations from H-14 to C-6/C-8/C-9/C-16, and the diagnostic chemical shifts of C-9 (*δ*_C_ 73.0), C-14 (*δ*_C_ 105.7), and C-16 (*δ*_C_ 54.0) revealed that the ketal fragment was located at C-14, leading to the formation of 2-methoxytetrahydrofuran. This moiety and bicyclo[3.1.1]heptane were connected via spiro carbon *C*6, based on the key HMBC correlations from H-14 to C-1/C-6, and from H-8 to C-1/C-5/C-6. The methacrylic acid group was attached to C-9, established by the HMBC correlations at H-13/C-10/C-11/C-12 and COSY correlation at H-9/H-10. Thus, compound **1** was elucidated as a bergamotane sesquiterpenoid derivative, bearing an unreported spiral 6/4/5 tricyclic ring system (Figure 2).

Brasilterpene B (**2**) had the same molecular formula and planar structure as **1**, suggested by the identical HRESIMS and highly similar NMR data (Table 1 and Figure 2). The slight differences in the chemical shifts between **1** and **2** were those of 2-methoxytetrahydrofuran moiety, which suggested that they may be configurational isomers.

**Figure 3:**
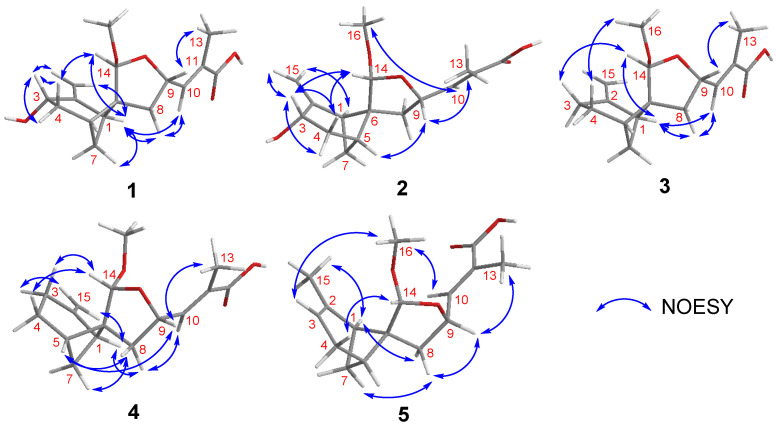
Key NOESY correlations of compounds **1**–**5**.

**Figure 4:**
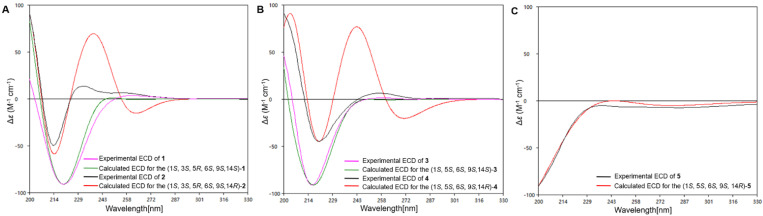
Calculated ECD spectra of compounds **1**–**5** and the experimental ECD curves: (**A**) ECD spectra of compounds **1** and **2**. (**B**) ECD spectra of compounds **3** and **4**. (**C**) ECD spectra of compound **5**.

**Figure 5:**
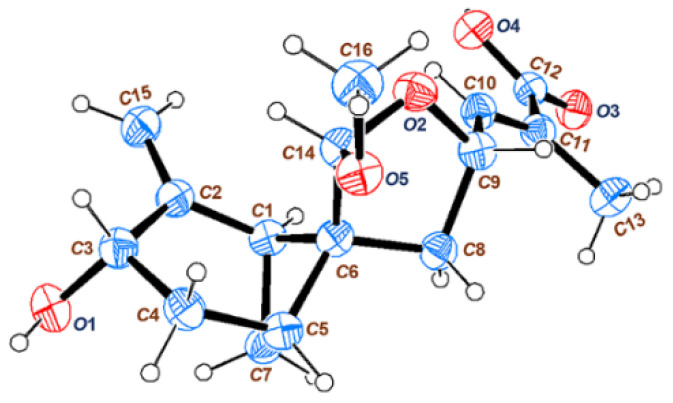
ORTEP drawing for the crystal structure of **1** (30% probability ellipsoids).

The relative configurations of **1** and **2** were determined by key NOESY correlations as shown in Figure 3. The NOESY correlation between H-9 and H_3_-13 in **1** and **2** indicated the *E* geometry of *△*^10^ double bond. The NOESY cross-peaks of H-14/H-1/H-10 in **1** indicated they were coplanar. Due to the rigidity of bicyclo[3.1.1]heptane ring system and the NOESY correlation of H-14/H-4a, the relative of compound **1** was suggested to be 1S*,5R*,6S*,9S*, and 14S*. However, there is no reliable evidence to determine the relative configuration of C-3. To distinguish the two possible epimers 1*S*,3*R*,5*R*,6*S*,9*S*,14*S*-**1** (**1a**) and 1*S*,3*S*,5*R*,6*S*,9*S*,14*S*-**1** (**1b**), the computations of ^1^H and ^13^C NMR chemical shifts for **1a** and **1b** were performed (Tables S7 and S8) [31]. The DP4+ probability analysis indicated that **1b** appeared consistent with the NMR experimental data with a probability of 100% (Table 2) [32]. The absolute configuration of **1** was further confirmed by ECD calculations [23] (Figure 4A) and the X-ray diffraction analysis (Figure 5) with a Flack parameter of -0.05(8). In compound **2**, the NOESY correlations of H-10/H_3_-16, H-5/H-9, and H-3/H-14/H-4a (*δ*_H_ 2.34) suggested that **2** was probably the C-14 epimer of **1** (Figure 3). Finally, the absolute configuration of **2** was determined by the good match of the experimental and calculated ECD curves (Figure 4A).

Compounds **3** and **4** were obtained as colorless oil. Both of their molecular formulas were deduced as C_16_H_22_O_4_ based on the HRESIMS data (*m/z* 277.1441 [M − H]^−^ and 277.1442 [M − H]^−^, respectively). The 1D NMR data of **3** (Table 3) were similar to those of **1**, except for the lack of the oxygen-linked methine (*δ*_H_ 4.28, *δ*_C_ 64.8) and the presence of a methylene (*δ*_H_ 2.53 and 2.31, *δ*_C_ 23.2) at 2-methylene-bicyclo[3.1.1]heptane moiety, which was confirmed by the ^1^H-^1^H COSY correlations between H-3/H-4/H-5/H-7/H-1 (Figure 2). The relative configuration of compound **3** was proved to be 1S*,5S*,6S*,9S*, and 14S*, based on the NOESY correlations of H-9/H_3_-13, H-10/H-1/H-14/H-3b (*δ*_H_ 2.31) and H-4a (*δ*_H_ 2.00)/H_3_-16 (Figure 3). A careful analysis of the HSQC, COSY, and HMBC spectra of **4** indicated that it possessed the same planar structure as **3** (Figure 2), and the NOESY cross-peaks of H-5/H-9/H_3_-13, H-10/H-8b (*δ*_H_ 1.78)/H-1, and H-3b (*δ*_H_ 2.37)/H-14/H-4 in **4**, suggested a different configuration of C-14 between **3** and **4** (Figure 3). The absolute configurations of **3** and **4** were subsequently determined by comparing the experimental and calculated ECD spectra (Figure 4B).

Brasilterpene E (**5**) showed a molecular formula of C_16_H_22_O_4_ deduced from the deprotonated HRESIMS peak at *m/z* 277.1444 (calcd. 277.1445) and similar NMR data to those of **4** (Table 3 and Table 4). The major differences were the replacement of the exocyclic double bond at 2-methylene-bicyclo[3.1.1]heptane moiety in **4** by an endocyclic one in **5**. The *△*^2^ double bond was further supported by the HMBC correlations of H-1(*δ*_H_ 2.12)/C-3 (*δ*_C_ 114.7), H_3_-15 (*δ*_H_ 1.66)/C-3, H-5 (*δ*_H_ 2.43)/C-3, and ^1^H-^1^H COSY correlations of H-3 (*δ*_H_ 5.15)/H-4/H-5/H-7/H-1 (Figure 2). The NOESY correlations of H-10/H_3_-16, H-13/H-9/H-8a (*δ*_H_ 2.35)/H-7a (*δ*_H_ 2.15), H-8b (*δ*_H_ 1.77)/H-1, H-4a (*δ*_H_ 2.29)/H-14, and H-3/H_3_-16 in **5** suggested the 1S*,5S*,6S*,9S*,14R* relative configuration, same as **4** (Figure 3). Then, the absolute configuration was determined to be 1*S*,5*S*,6*S*,9*S*, and 14*R* by comparing its experimental and calculated ECD spectra (Figure 4C).

**Scheme 1:**
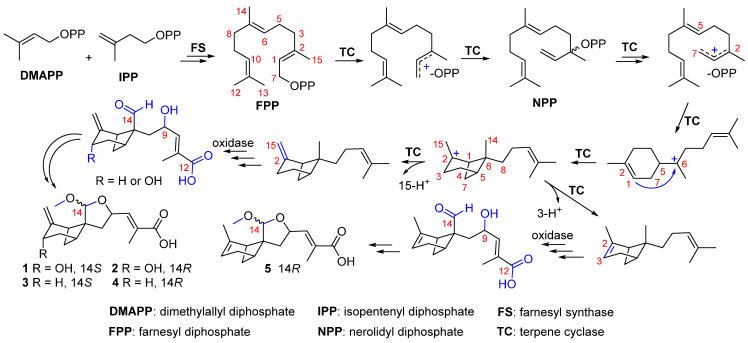
Proposed biosynthetic pathway for compounds **1**–**5**.

As a class of unusual bergamotane-sesquiterpenoid derivates, brasilterpenes A–E (**1**–**5**), were proposed to be biosynthesized by the farnesyl diphosphate (FPP) synthesis pathway (Scheme 1). The bergamotane-sesquiterpenoid skeleton was cyclized from FPP via nerolidyl diphosphate (NPP) followed by a bisabolyl cation intermediate [33]. Then compounds **1**–**5** were generated by further oxidation, nucleophilic attack of OH-9, and methylation. Due to the flexibility of nucleophilic attack direction during the formation of the furan ring, compounds **1**–**4** appeared in pairs as C-14 epimers.

**Figure 6:**
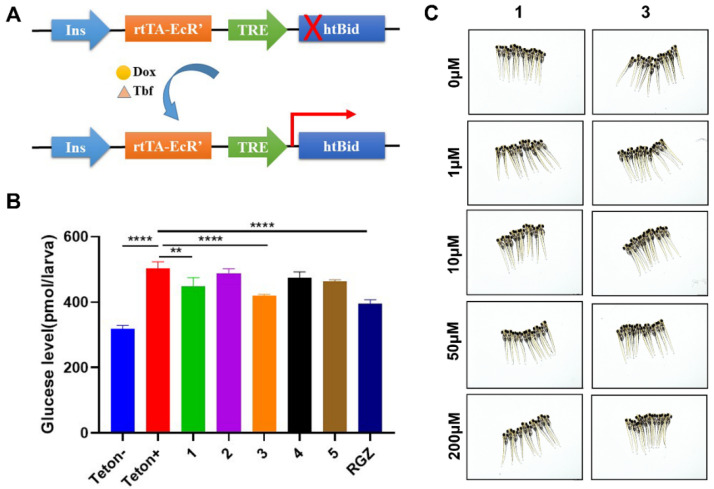
Compounds **1** and **3** had hypoglycemic activity and low toxic effect in zebrafish: (**A**) Schematic diagram of β-cell ablation of transgenic zebrafish *Tg(-1.2ins:htBid^TE−ON^; LR).* In the transgene, the proapoptotic gene human truncated Bid (htBid) is under the control of a TRE-based promoter that is activated by a TE-ON driven by the zebrafish insulin promoter. After incubation with doxycycline (Dox) and tebufenozide (Tbf), the htBid will express and cause β-cell apoptosis. (**B**) The total free glucose level of 6 dpf *Tg(-1.2ins:htBid^TE−ON^; LR)* was treated with 10 μM of different compounds. After induction of the β-cell ablation, 10 μM of each compound was incubated with these larvae individually for 24 h, then their glucose levels were measured. Teton−, without β-cell ablation. Teton+, β-cell ablation without compound incubation. Rosiglitazone (RGZ) was used as a positive control. The values shown are means ± SEM from three independent experiments. ** *p* < 0.01, and **** *p* < 0.0001 by one-way ANOVA. (**C**) Compounds **1** and **3** did not show any toxic effect in zebrafish up to 200 μM, and the concentration of each compound was indicated.

To screen these compounds for hypoglycemic activity, the β-cell-specific ablation transgenic zebrafish, *Tg(-1.2ins:htBid^TE−ON^; LR)* [34], was applied. In this transgenic line, the proapoptotic protein human truncated Bid (htBid) is target expressed in the β cells under the control of the tetracycline and ecdysone-inducible system (Figure 6A) [35]. After the zebrafish is incubated with doxycycline (Dox) and tebufenozide (Tbf), the β cells of the zebrafish can be induced to express the htBid protein, which results in the ablation of β cells and increased free blood glucose (Figure 6B, Teton− vs. Teton+). This model was then used to assess all isolated compounds (**1**–**5**). After the incubation of β-cell-ablated zebrafish larvae with 10 μM of these five compounds for 24 h, the total glucose level from each group was measured. As shown in Figure 6B, the free glucose level of the β-cell-ablated group (Teton+) was 502.8 ± 12.2 pmol/larva, while compounds **1** and **3** significantly reduced the glucose levels to 449.3 ± 15.1 and 420.4 ± 2.3 pmol/larva, respectively. The hypoglycemic activity of compound **3** was comparable to the positive control, anti-diabetes drug rosiglitazone (RGZ, with a glucose level of 395.6 ± 6.6 pmol/larva). Moreover, compounds **1** and **3** did not show any toxic effect to the zebrafish larvae up to 200 μM (Figure 6C).

The hypoglycemic activity of compounds **1** and **3** may be related to the *S* configuration at C-14, which was the unique structural difference compared to **2** and **4**. Compound **3** was more active than **1**, which indicated that the presence of the hydroxyl group at C-3 may reduce the blood glucose level. Similarly, compound **4** was more active than **2**, which illustrated the above reduction. Furthermore, compound **5** was more active than **4**, which suggests that the endocyclic *△*^2^ double bond may increase hypoglycemic activity.

**Figure 7:**
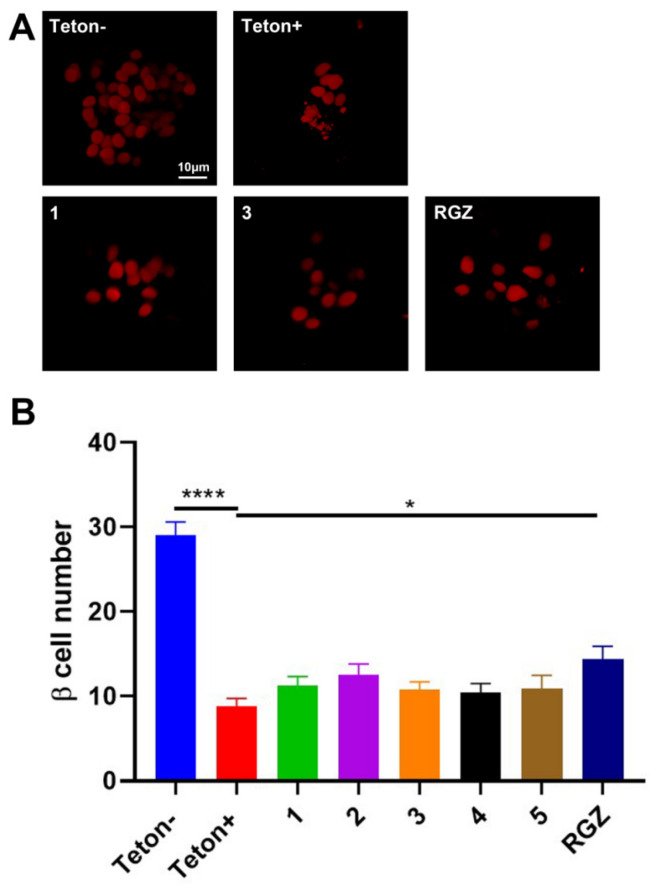
Compounds **1** and **3** did not increase β-cell regeneration: (**A**) Representative confocal images of the β-cell number from *Tg(-1.2ins:htBid^TE−ON^; LR)*; *Tg(-1.2ins:H2BmCherry)* larvae treated with or without 10 μM of compounds. Scale bar indicates 10 μm. (**B**) Quantification of β-cell number from the larvae treated with or without compounds. Teton−, without β-cell ablation. Teton+, β-cell ablation without compound incubation. Rosiglitazone (RGZ) was used as control. The values shown are means ± SEM from three independent experiments. * *p* < 0.05, and **** *p* < 0.0001 by one-way ANOVA.

The hypoglycemic effects may be due to increased β-cell mass or increased glucose uptake. To survey whether compounds **1** and **3** induce β-cell regeneration in *Tg(-1.2ins:htBid^TE−ON^; LR)*, the *Tg(-1.2ins:htBid^TE−ON^; LR)* was crossed with *Tg(-1.2ins:H2BmCherry)* whose β cells were labeled with mCherry fluorescence. Without the induction, the β-cell number of the double transgenic *Tg(-1.2ins:htBid^TE−ON^; LR); Tg(-1.2ins:H2BmCherry)* larvae was 29.0 ± 1.6 (Figure 7, Teton−). While the β-cell number significantly decreased to 8.8 ± 1.6 after induction (Figure 7, Teton+). However, treated with 10 μM, compounds **1**–**5** did not change the β-cell number in each group (Figure 7), which suggested that the hypoglycemic activity of compounds **1** and **3** was not due to β-cell regeneration.

**Figure 8:**
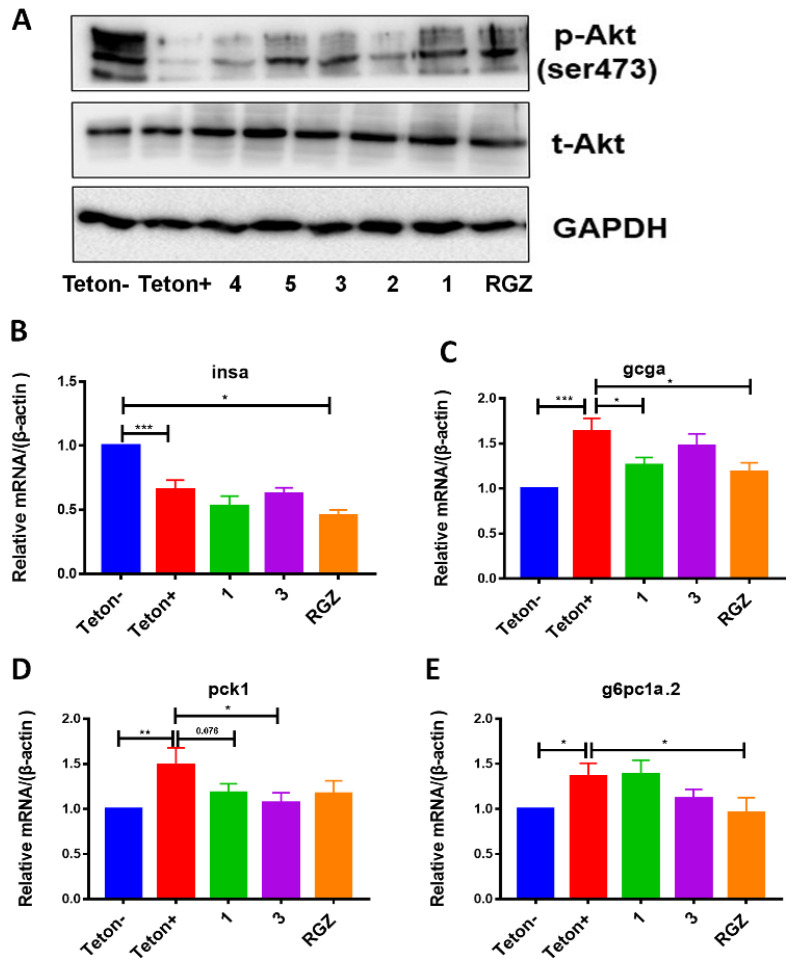
Compounds **1** and **3** improved insulin sensitivity and suppressed gluconeogenesis: (**A**) Western blot for phosphorylated Akt (p-Akt) and total Akt (t-Akt) in ablated *Tg(-1.2ins:htBid^TE−ON^; LR)* larvae treated with or without 10 μM of compounds. GAPDH was used as an internal control. (**B**–**E**) Quantitative RT-PCR analysis of the expression of key gluconeogenic genes *insa* (**B**), *gcga* (**C**), *pck1* (**D**), and *g6pc1a.2* (**E**). Expression of β-actin was used as an internal control for all real-time PCR experiments. Teton−, without β-cell ablation. Teton+, β-cell ablation without compound incubation. Rosiglitazone (RGZ) was used as a control compound. All the values shown are means ± SEM from three independent experiments. * *p* < 0.05, ** *p* < 0.01, and *** *p* < 0.001 by one-way ANOVA.

The hypoglycemic mechanism of compounds **1** and **3** was then investigated**.** The insulin signaling sensitivity, which is frequently indicated by the phosphorylation of Akt, was first determined. The Akt phosphorylation of 10 μM of compounds treated β-cell-ablated *Tg(-1.2ins:htBid^TE−ON^; LR)* was detected by Western blot. As shown in Figure 8A, the levels of Akt phosphorylation in **1** and **3** were increased, which suggested increased insulin sensitivity. The genes involved in glucose homeostasis were also assessed, including *insa* (insulin a), *gcga* (glucagon a), *pck1* (phosphoenolpyruvate carboxykinase 1), and *g6pc1a.2* (glucose-6-phosphatase catalytic subunit 1a, tandem duplicate 2). Ablation of β cells significantly decreases *insa* expression (Figure 8B, Teton+ vs. Teton−1). However, compounds **1** and **3** treatment did not change the *insa* levels (Figure 8B). For the gluconeogenic genes, *gcga*, *pck1,* and *g6pc1a.2,* compound **1** treated larvae significantly decreased *gcga*, and **3** have a similar trend but without significance (Figure 8C). Compound **3** significantly down-regulated the *pck1* expression*,* and **1** has a similar trend with the *p* value of 0.076 (Figure 8D). Compound **3** treatment reduced the *g6pc1a.2* level, albeit there is no significance (Figure 8E). These data suggested that compounds **1** and **3** suppressed gluconeogenesis.

## 3. Materials and Methods

### 3.1. General Experimental Procedures

Optical rotations were obtained on a P-1020 digital polarimeter (JASCO, Tokyo, Japan) using MeOH as the solvent at 25 °C. HRESIMS spectra were measured on a Micromass EI-4000 (Autospec-Ultima-TOF, Waters, Shanghai, China). NMR spectra was performed on BRUKER AVANCE NEO 400 MHz (Bruker, Beijing, China) and Agilent 500 MHz DD2 (Agilent, Beijing, China) and JEOL JNM-ECP600 spectrometers (JEOL, Beijing, China), and the 1D and 2D spectra were referenced to the residual deuterated solvent peaks at *δ*_C_ 39.52 and *δ*_H_ 2.50 (DMSO-*d*_6_). UV data were measured on a Beckman DU 640 spectrophotometer (Beckman Ltd., Shanghai, China). IR spectra were recorded on a Nicolet NEXUS 470 spectrophotometer (Thermo Scientific, Beijing, China) in KBr disks. A JASCO J-715 spectropolarimeter (JASCO, Tokyo, Japan) was used to obtain ECD spectra. X-ray crystal data were measured on an Agilent Gemini Ultra diffractometer with Cu Kα radiation. Silica gel (100–200 and 200–300 mesh, Qingdao Marine Chemical Factory, Qingdao, China), reversed-phase silica gel (50 μm; Silicycle, Shanghai, China), and Sephadex LH-20 gel (GE Healthcare, Bio-Sciences Corp, Piscataway, NJ, USA) were used for column chromatography (CC). An ODS column (YMC-Pack ODS-A, 10 × 250mm, 5 μm) was used for semi-preparative HPLC.

### 3.2. Fungal Material

The fungus was isolated from deep-sea sediment from the Indian Ocean (depth 3824 m, 15.66° S, 88.00° E, collected in May 2014). The strain was deposited at the Key Laboratory of Marine Drugs, the Ministry of Education of China, School of Medicine and Pharmacy, Ocean University of China, Qingdao, People’s Republic of China. The fungal strain was identified as *P. brasiliense* according to its morphological characteristics and by comparison of the ITS sequence amplification. The ITS sequence was deposited at GenBank with accession number ON025790.

### 3.3. Fermentation, Extraction, and Isolation

Erlenmeyer flasks (1000 mL) containing 300 mL of 2^#^ media were directly inoculated with spores of fungus under static condition at room temperature for 30 days. The 2^#^ media was composed of mannitol (2.0%), yeast extract (0.3%), monosodium glutamate (1.0%), glucose (1.0%), maltose (2.0%), corn steep liquor (0.1%), KH_2_PO_4_ (0.05%), MgSO_4_·7H_2_O (0.03%) in natural seawater (collected from Jiao Zhou Bay, Qingdao, China). The fermentation broth (40 L) was filtered through muslin cloth to separate the supernatant from the mycelia. The mycelia were macerated and extracted with MeOH, then were concentrated to afford an aqueous solution. The fermentation broth combined with the aqueous solution was extracted three times with ethyl acetate and concentrated to give the crude extract (38 g).

The EtOAc extract was subjected to silica gel column chromatography (DCM, MeOH, v/v, gradient 100:0−0:100) to generate seven fractions, Fr.1–Fr.7. The Fr.5 (1.53 g) was separated by VLC of ODS to afford twenty fractions (Fr.5-1 to Fr.5-20) by elution with a stepped gradient of mixtures of MeOH-H_2_O (from 15:85 to 65:35). Fr.5-3 (123.5 mg) was further fractioned by a Sephadex LH-20 column with MeOH to provide three subfractions (Fr.5-3-1 to Fr.5-3-3). The purification of Fr.5-3-2 (74.2 mg) gave compounds **1** (*t*_R_ = 21.8 min, 18.3 mg) and **2** (*t*_R_ = 18.1 min, 12.4 mg) using RP-HPLC (MeOH-H_2_O, 30:70, 1‰ CF_3_COOH), respectively. Fr.4 (1.37 g) was also separated by VLC (ODS) using a stepped gradient elution of MeOH-H_2_O (10:90 to 100:0) to yield 18 subfractions (Fr.4-1 to Fr.4-18). Fr.4-11 (185.7 mg) and Fr.4-12 (84.2 mg) were subjected to Sephadex LH-20 column with MeOH to provide four and three subfractions (Fr.4-11-1 to Fr.4-11-4, Fr.4-12-1 to Fr.4-12-3), respectively. Fr.4-11-4 (65.7 mg) were further purified using RP-HPLC (MeOH-H_2_O, 70:30 and MeCN-H_2_O, 55:45, 1‰ CF_3_COOH) to yield compounds **3** (10.1 mg, *t*_R_ = 16.2 min) and **4** (6.8 mg, *t*_R_ = 13.7 min), respectively. Compound **5** (3.4 mg, *t*_R_ = 15.1 min) was isolated from Fr.4-12-3 (16.6 mg) by RP-HPLC using MeCN-H_2_O (60:40) with 1‰ CF_3_COOH.

Brasilterpene A (**1**): colorless crystal; ${\left[\alpha \right]}_{D}^{25}$ +16.7 (*c* 0.27, MeOH); UV (MeOH) *λ*_max_ (log *ε*) 206 (2.15), 220 (2.11) nm; IR (KBr) *ν*_max_ 3421, 2934, 1699, 1653, 1025 cm^−1^; ECD (4.5 mM, MeOH) *λ*_max_ (Δ*ε*) 200 (+3.81), 220 (−17.01), 258 (+0.98) nm; ^1^H and ^13^C NMR data, Table 1; HRESIMS *m/z* [M − H]^−^ 293.1393 (calcd for C_16_H_21_O_5_, 293.1394).
Brasilterpene B (**2**): colorless oil; ${\left[\alpha \right]}_{D}^{25}$ −30.2 (*c* 0.23, MeOH); UV (MeOH) *λ*_max_ (log *ε*) 206 (2.01), 220 (2.36) nm; IR (KBr) *ν*_max_ 3412, 2933, 1699, 1652, 1024, 993 cm^−1^; ECD (3.8 mM, MeOH) *λ*_max_ (Δ*ε*) 200 (+12.53), 220 (−7.05), 234 (+2.48) nm; ^1^H and ^13^C NMR data, Table 1; HRESIMS *m/z* [M − H]^−^ 293.1391 (calcd for C_16_H_21_O_5_, 293.1394).
Brasilterpene C (**3**): colorless oil; ${\left[\alpha \right]}_{D}^{25}$ +11.9 (*c* 0.33, MeOH); UV (MeOH) *λ*_max_ (log *ε*) 206 (2.51), 217 (2.50) nm; IR (KBr) *ν*_max_ 3394, 2937, 1699, 1653, 1207, 1020 cm^−1^; ECD (6.0 mM, MeOH) *λ*_max_ (Δ*ε*) 200 (+14.98), 217 (−31.0), 255 (+0.45) nm; ^1^H and ^13^C NMR data, Table 3; HRESIMS *m/z* [M − H]^−^ 277.1441 (calcd for C_16_H_21_O_4_, 277.1445).
Brasilterpene D (**4**): colorless oil; ${\left[\alpha \right]}_{D}^{25}$ −58.2 (*c* 0.37, MeOH); UV (MeOH) *λ*_max_ (log *ε*) 210 (2.06), 223 (2.29) nm; IR (KBr) *ν*_max_ 3402, 2937, 1699, 1207, 992 cm^−1^; ECD (5.4 mM, MeOH) *λ*_max_ (Δ*ε*) 200 (+21.87), 217 (−10.78), 258 (+2.01) nm; ^1^H and ^13^C NMR data, Table 3; HRESIMS *m/z* [M − H]^−^ 277.1442 (calcd for C_16_H_21_O_4_, 277.1445).
Brasilterpene E (**5**): colorless oil; ${\left[\alpha \right]}_{D}^{25}$ −104.2 (*c* 0.19, MeOH); UV (MeOH) *λ*_max_ (log *ε*) 221 (3.12) nm; IR (KBr) *ν*_max_ 3388, 2936, 1696, 1207, 1093, 1037, 992 cm^−1^; ECD (4.2 mM, MeOH) *λ*_max_ (Δ*ε*) 200 (−14.97), 237 (−0.82), 280 (−1.20) nm; ^1^H and ^13^C NMR data, Table 4; HRESIMS *m/z* [M − H]^−^ 277.1444 (calcd for C_16_H_21_O_4_, 277.1445).

### 3.4. X-ray Crystallographic Analysis of Brasilterpene A (**1**)

A crystal of compound **1** suitable for X-ray diffraction was obtained by slow evaporation of a solution in MeOH. Single-crystal X-ray diffraction data were measured with an Agilent Gemini Ultra diffractometer with Cu Kα radiation (*λ* = 1.541 84 Å). The structure was solved by direct methods (SHELXS-97) and refined using full-matrix least-squares difference Fourier techniques. Crystallographic data for **1** have been deposited in the Cambridge Crystallographic Data Center (CCDC No. 2160281). A copy of the data can be obtained, free of charge, on application to the Director, CCDC, 12 Union Road, Cambridge CB2 1EZ, UK (e-mail: deposit@ccdc.cam.ac.uk).

Crystal data for Brasilterpene A (**1**): C_16_H_22_O_5_, fw = 294.33, space group *P*2_1_2_1_2, unit cell dimensions *a* = 23.288(2) Å, *b* = 6.5246(9) Å, *c* = 10.1849(14) Å, *V* = 1535.6(3) Å3, *α* = *γ* = 90°, *β* = 97.116(5)°, *Z* = 4, *D*_calcd_ = 1.273 mg/m^3^, crystal size 0.28×0.08×0.12 mm^3^, *μ* = 0.774 mm^−1^, and *F*(000) = 632.0. Independent reflections: 2782 (R_int_ = 0.0233). The final refinement gave *R*_1_ = 0.0700 and *wR*_2_ = 0.1498 (*I* > 2*σ*(*I*)). The absolute structure parameter was −0.05(8).

### 3.5. Computational Section

Monte Carlo conformational searches were carried out with *Spartan’*14 software using the Merck Molecular Force Field (MMFF)[36]. Two diastereomers of compound **1** were subjected to conformational analysis and geometrical optimization using B3LYP/6-31+G(d) in DMSO (PCM) in Gaussian 09 [37]. NMR chemical shift calculation was performed with the GIAO method using B3LYP/6-311G+(d,p) in DMSO (PCM). All chemical shifts were Boltzmann averaged, and the shielding constant was used for DP4+ probability calculation based on the method [32]. As for the calculation of ECD, the conformers of compounds **1**–**4** were initially optimized at the B3LYP/6-31+G(d) in MeOH (PCM), and compound **5** was optimized at the B3LYP/TZVP in MeOH (PCM). The theoretical calculation of the ECD spectra was conducted in MeOH using density functional theory (DFT) with B3LYP and 6-31+G(d) basis set in Gaussian 09 for all conformers of compounds **1**–**5**. ECD spectra were generated using the program SpecDis [38] by applying a Gaussian band shape with a 0.30 eV width for **1**, **3,** and **4**, a 0.40 eV width for **2**, and a 0.65 eV width for **5**. The calculated spectra were shifted by −6 nm for **1**, −3 nm for **2**, −14 nm for **3**, and −30 nm for **5** to facilitate comparison to the experimental data.

### 3.6. Assays of Free Blood Glucose in Hyperglycemic Zebrafish

The *Tg(-1.2ins:htBid^TE−ON^; LR)* were treated with 25 μM doxycycline hyclate (Sigma-Aldrich, Shanghai, China) and 50 μM tebufenozide (Sigma-Aldrich) in 0.3X Danieau solution for 48 h (from 3 to 5 dpf) before compounds **1**–**5** treatment.

After 48 h of drug-induced β-cell ablation, larvae were rinsed with 0.3X Danieau solution to remove the inducer drugs. Then the zebrafish larvae were cultured in 12-well plates incubated with each compound at a final concentration of 10 μM, respectively. For the Teton− (without β-cell ablation) and Teton+ (β-cell ablation but did not add compounds) group, the DMSO was added as a control.

Free glucose was determined by a glucose assay kit (A22189, Amplex Red Glucose/Glucose Oxidase Assay Kit) similar to Li et al. [39]. After treatment of the compounds for 24 h, a pool of 10 larvae was homogenized in 100 μL of sample buffer. The homogenate was spun at 10,000 rpm for 10 min. Then the supernatant was determined according to the manufacturer’s instructions. Each sample was measured for three pools.

### 3.7. Evaluation of Toxicological Effect in Zebrafish Larvae

Five dpf zebrafish larvae were used to evaluate the potential toxicity exerted by compounds **1** and **3** in vivo. Twenty zebrafish larvae were selected and equally placed in 12-well plates and incubated with compounds **1** and **3** from five dpf to six dpf. Each compound with final concentration of 1, 5, 10, 50, and 200 μM was added to the wells, and the DMSO was added separately as the control well. After 24 h of incubation, the mortality and morphological abnormality were observed using a Leica stereomicroscope.

### 3.8. Assays of the Hypoglycemic Mechanism of Compounds **1** and **3**

After larvae were treated with compounds for 24 h, a pool of 20 larvae was homogenized in 200 μL of RIPA lysis buffer. The homogenate was spun at 4 °C, 10,000 rpm for 10 min. Then the supernatant was denatured for 10 min at 95 °C with a loading buffer. Western blot analysis was carried out using antibodies of p-Akt (Ser473) (1:1000, Proteintech 66444-1-Ig), t-Akt (1:1000, Proteintech 10176-2-AP), and GAPDH (1:5000, Proteintech 60004-1-Ig).

After larvae were treated with compounds for 24 h, a pool of 30 larvae was homogenized in 1000 μL of Trizol Reagents (Thermo Fisher, Waltham, MA, USA). Ensure that the ratio of 260 to 280 extracted RNA samples was between 1.0 and 2.0. Then the RNA was reverse transcribed by the M-MLV (Promega, Madison, WI, USA) with oligo(dT)16 primers. On a real-time PCR system, the cDNA product was subject to qRT-PCR using the Power SYBR green PCR master mix (Lifeint, Xiamen, China). QRT-PCR was performed using 95 °C for 2 min, then 95 °C for 15 s, 60 °C for 1 min for 40 cycles. The following primers were used: insa: 5′-GTAAGCACTAACCCAGGCACA-3′ and 5′-GGGCAGATTTAGGAGGAAGG-3′; gcga: 5′-CGACAGCACAAGCACAGAGACAG-3′ and 5′-GACGTTTGACAGAACCACCATTTC-3′; pck1: 5′-CCACTCAAGAAGCCGCTGGTCA-3′ and 5′-CCCTCCTCTTTAGCGATGCGTGA-3′; g6pc1a.2: 5′-CTGGTCACCTCCATCCTCGCCAT-3′ and 5′-CATGCCTGAAATGACACCAGCGAA-3′; β-actin: 5′-ACAGGGAAAAGATGACACAG-3′ and 5′-AGAGTCCATCACGATACCAG-3′.

### 3.9. Statistical Analysis

Statistical analysis was performed using GraphPad PRISM 8 software. Results are presented as mean values ± SEM. No outlying values were excluded from the datasets used for statistical analysis. The statistics were performed using one-way ANOVA followed by Bonferroni post hoc test or *t*-test. *p* < 0.05 was considered significant.

## 4. Conclusions

In summary, five unusual bergamotane sesquiterpenoid derivatives were isolated from the marine-derived fungus *P. brasiliense* HDN15-135. Compounds **1**–**5** contained unreported skeleton with a 6/4/5 tricyclic ring system, which was reported for the first time. Compounds **1** and **3** significantly reduced the glucose levels in the hyperglycemic zebrafish model, and the hypoglycemic mechanism of compounds **1** and **3** was proposed to improve insulin sensitivity and suppress gluconeogenesis rather than induce pancreatic β cell regeneration. The study of structure–activity relationship showed that the *S* configuration at C-14, the presence of endocyclic double bond (*△*^2^), and the absence of hydroxyl at C-3 may contribute to the improvement of hypoglycemic activity. Therefore, this study may provide a valuable structural template for the development of hypoglycemic drugs.

## Figures and Tables

**Table 1:** ^1^H NMR (400 MHz) and ^13^C NMR spectroscopic data of **1** and **2** in DMSO-*d*_6_.

No.	1 *^a^*		2 *^b^*	
	*Δ*c, Type	*δ*_H_ (*J* in Hz)	*δ*c, Type	*δ*_H_ (*J* in Hz)
1	51.1, CH	2.61, t (5.6)	47.5, CH	2.74, t (5.6)
2	153.8, C		154.3, C	
3	64.8, CH	4.28, m	64.6, CH	4.33, m
4	35.9, CH_2_	2.38, m	36.0, CH_2_	2.34, m
		1.64, dt (13.7, 3.2)		1.70, dt (13.9, 3.4)
5	38.7, CH	2.23, m	38.44, CH	2.24, m
6	54.2, C		54.4, C	
7	29.1, CH_2_	2.17, m	31.0, CH_2_	2.26, m
		1.55, d (9.0)		1.40, d (8.2)
8	40.8, CH_2_	2.42, dd (12.7, 8.9)	38.42, CH_2_	2.46, dd (12.0, 6.8)
		1.97, dd (12.7, 5.0)		1.80, dd (12.0, 9.4)
9	73.0, CH	4.81, m	73.6, CH	4.83, m
10	142.1, CH	6.60, dd (7.9, 1.4)	143.1, CH	6.50, dd (8.2, 1.4)
11	128.3, C		127.9, C	
12	168.6, C		168.7, C	
13	12.7, CH_3_	1.77, d (1.4)	12.4, CH_3_	1.76, d (1.4)
14	105.7, CH	4.37, s	104.9, CH	4.48, s
15	110.7, CH_2_	5.03, t (1.6)	108.7, CH_2_	4.94, t (1.9)
		4.80, t (1.6)		4.71, t (1.9)
16	54.0, CH_3_	3.20, s	54.3, CH_3_	3.16, s
OH-3		4.90, d (4.7)		4.91, d (5.4)
COOH-12		12.40, s		12.29, s

*^a^* DEPTQ-NMR measured at 100 MHz. *^b^* DEPTQ-NMR measured at 125 MHz.

**Table 2:** DP4+ analysis using the calculated shielding tensors for compounds **1a** (1*S*,3*R*,5*R*,6*S*,9*S*,14*S*-**1**) and **1b** (1*S*,3*S*,5*R*,6*S*,9*S*,14*S*-**1**).

	Data Type	1a	1b
sDP4+^*a*^	^1^H	0.00%	100.00%
	^13^C	0.89%	99.11%
	^1^H + ^13^C	0.00%	100.00%
uDP4+^*b*^	^1^H	0.00%	100.00%
	^13^C	4.59%	95.41%
	^1^H + ^13^C	0.00%	100.00%
DP4+	^1^H	0.00%	100.00%
	^13^C	0.04%	99.96%
	^1^H + ^13^C	0.00%	100.00%

*^a^* sDP4+: scaled DP4+. *^b^* uDP4+: unscaled DP4+.

**Table 3:** ^1^H NMR (500 MHz) and ^13^C NMR (100 MHz) spectroscopic data of **3** and **4** in DMSO-*d*_6_.

No.	3		4	
	*δ*c, Type	*δ*_H_ (*J* in Hz)	*δ*c, Type	*δ*_H_ (*J* in Hz)
1	51.4, CH	2.55, t (5.6)	47.8, CH	2.72, t (5.4)
2	149.9, C		149.7, C	
3	23.2, CH_2_	2.53, m	23.0, CH_2_	2.55, m
		2.31, m		2.37, m
4	23.4, CH_2_	2.00, m	23.3, CH_2_	1.87, m
		1.76, m		
5	38.8, CH	2.28, m	38.4, CH	2.24, m
6	54.5, C		54.7, C	
7	26.5, CH_2_	2.15, m	28.1, CH_2_	2.22, m
		1.38, d (9.5)		1.30, d (8.9)
8	40.3, CH_2_	2.41, dd (12.5, 9.3)	38.1, CH_2_	2.45, dd (11.9, 6.8)
		1.97, dd (12.6, 4.4)		1.78, m, overlapped
9	73.1, CH	4.83, m	73.6, CH	4.84, m
10	142.5, CH	6.61, d (7.8)	143.3, CH	6.51, d (8.2)
11	128.0, C		127.7, C	
12	168.6, C		168.7, C	
13	12.7, CH_3_	1.77, s	12.4, CH_3_	1.77, s
14	105.6, CH	4.48, s	104.7, CH	4.52, s
15	107.6, CH_2_	4.71, s	106.9, CH_2_	4.61, s
		4.63, s		4.58, s
16	53.9, CH_3_	3.21, s	54.4, CH_3_	3.18, s
COOH-12		12.40, s		12.36, s

**Table 4:** ^1^H NMR (600 MHz) and ^13^C NMR (100 MHz) spectroscopic data of **5** in DMSO-*d*_6_.

No.	5		No.	5	
	*δ*c, Type	*δ*_H_ (*J* in Hz)		*δ*c, Type	*δ*_H_ (*J* in Hz)
1	42.8, CH	2.12, m	9	75.4, CH	4.94, m
2	145.6, C		10	143.2, CH	6.53, d (7.6)
3	114.7, CH	5.15, m	11	127.6, C	
4	30.6, CH_2_	2.29, m	12	168.9, C	
		2.25, m	13	12.0, CH_3_	1.76, s
5	38.4, CH	2.43, d (3.9)	14	105.5, CH	4.42, s
6	53.3, C		15	22.5, CH_3_	1.66, m
7	29.9, CH_2_	2.15, m	16	54.3, CH_3_	3.18, s
		1.18, d (8.1)	COOH-12		12.29, s
8	38.2, CH_2_	2.35, dd (12.0, 6.8)			
		1.77, dd (12.0, 9.9)			

## Data Availability

The data presented in this study are available in this article and supplementary material.

## Supplementary Materials

The following supporting information can be downloaded at: https://www.mdpi.com/article/10.3390/md20050338/s1, Figure S1: HPLC analysis of the crude extract of HDN15-135; Figure S2: Structures of reported bergamotane sesquiterpenoids; Figures S3–S42: HRESIMS spectra, IR spectra, and 1D and 2D NMR spectra of **1**–**5**; Figures S43 and S44: DFT-optimized structures for low-energy conformers of **1a** and **1****b**; Figure S45: Correlation plots of experimental ^13^C NMR chemical shifts in **1** corresponding calculated ^13^C NMR chemical shifts in **1a** and **1b**. Figures S46–S50: DFT-optimized structures for low-energy conformers of **1**–**5**; Tables S1, S4, S10, S13, S16, S19, and S22: Harmonic frequencies (cm**^−1^) of **1a**, **1b**, and **1**–**5**; Tables S2, S5, S11, S14, S17, S20, and S23: Important thermodynamic parameters (a.u.) and Boltzmann distributions of the optimized **1a**, **1b,** and **1**–**5**; Tables S3, S6, S12, S15, S18, S21, and S24: Cartesian coordinates of the low-energy re-optimized conformers of **1a**, **1b**, and **1**–**5**; Tables S7 and S8: Calculated ^1^H and ^13^C NMR chemical shifts in **1a** and **1****b**; Table S9: DP4+ analysis using the calculated shielding tensors for **1a** and **1****b**.
