# Supplementary material for: Brasilterpenes A–E, Bergamotane Sesquiterpenoid Derivatives with Hypoglycemic Activity from the Deep Sea-Derived Fungus Paraconiothyrium brasiliense HDN15-135
Source: Mar Drugs. 2022 May 23;20(5):338. doi: 10.3390/md20050338 (PMC9143538; doi:10.3390/md20050338)
Supplement: Supplementary file 1 [file marinedrugs-20-00338-s001.zip › marinedrugs-1726405-supplementary.pdf]

# Supplementary Materials

Brasilterpenes A-E, bergamotane sesquiterpenoid derivatives with hypoglycemic activity from the deep sea-derived fungus *Paraconiothyrium brasiliense* HDN15-135

Wenxue Wang [1,‡], Yeqin Shi [2,‡], Yuanyuan Liu [1], Yundong Zhang [1], Jiajin Wu [1], Guojian Zhang [1,3], Qian Che [1], Tianjiao Zhu [1], Mingyu Li [2,*], and Dehai Li [1,3,*]

[1] Key Laboratory of Marine Drugs, Chinese Ministry of Education, School of Medicine and Pharmacy, Ocean University of China, Qingdao 266003, China; bx_wwx@163.com (W.W.); lyy19970310@163.com (Y.L.); zhangyundong0908@163.com (Y.Z.); wjj981123@163.com (J.W.); zhangguojian@ouc.edu.cn (G.Z.); cheqian064@ouc.edu.cn (Q.C.); zhutj@ouc.edu.cn (T.Z.)

[2] Fujian Provincial Key Laboratory of Innovative Drug Target Research, School of Pharmaceutical Science, Xiamen University, Xiamen 361102, China; syq001235@163.com (Y.S.)

[3] Laboratory for Marine Drugs and Bioproducts of Qingdao National Laboratory for Marine Science and Technology, Qingdao 266237, China

# Contents


**Figure S1** HPLC analysis of the crude extract of HDN15-135....................................1

**Figure S2** Structures of reported bergamotane sesquiterpenoids.................................1

**Figure S3** HRESIMS spectrum of brasilterpene A (**1**).................................................4

**Figure S4** IR spectrum (KBr) of brasilterpene A (**1**)....................................................4

**Figure S5** $^1$H NMR spectrum (DMSO-$d_6$, 400 MHz) of brasilterpene A (**1**)................5

**Figure S6** DEPTQ-NMR spectrum (DMSO-$d_6$, 100 MHz) of brasilterpene A (**1**).......5

**Figure S7** HSQC spectrum of brasilterpene A (**1**)........................................................6

**Figure S8** HMBC spectrum of brasilterpene A (**1**) .......................................................6

**Figure S9** $^1$H-$^1$H COSY spectrum of brasilterpene A (**1**).............................................7

**Figure S10** NOESY spectrum of brasilterpene A (**1**) ...................................................7

**Figure S11** HRESIMS spectrum of brasilterpene B (**2**)...............................................8

**Figure S12** IR spectrum (KBr) of brasilterpene B (**2**) .................................................8

**Figure S13** $^1$H NMR spectrum (DMSO-$d_6$, 400 MHz) of brasilterpene B (**2**).............9

**Figure S14** DEPTQ-NMR spectrum (DMSO-$d_6$, 125 MHz) of brasilterpene B (**2**).....9

**Figure S15** HSQC spectrum of brasilterpene B (**2**) ...................................................10

**Figure S16** HMBC spectrum of brasilterpene B (**2**) ..................................................10

**Figure S17** $^1$H-$^1$H COSY spectrum of brasilterpene B (**2**).........................................11

**Figure S18** NOESY spectrum of brasilterpene B (**2**)..................................................11

**Figure S19** HRESIMS spectrum of brasilterpene C (**3**).............................................12

**Figure S20** IR spectrum (KBr) of brasilterpene C (**3**) ...............................................12

**Figure S21** $^1$H NMR spectrum (DMSO-$d_6$, 500 MHz) of brasilterpene C (**3**)...........13

**Figure S22** $^{13}$C NMR spectrum (DMSO-$d_6$, 100 MHz) of brasilterpene C (**3**) ..........13

**Figure S23** HSQC spectrum of brasilterpene C (**3**) ...................................................14

**Figure S24** HMBC spectrum of brasilterpene C (**3**) ..................................................14

**Figure S25** $^1$H-$^1$H COSY spectrum of brasilterpene C (**3**).........................................14

**Figure S26** NOESY spectrum of brasilterpene C (**3**).................................................15

**Figure S27** HRESIMS spectrum of brasilterpene D (**4**) ............................................15

**Figure S28** IR spectrum (KBr) of brasilterpene D (**4**) ...............................................16

**Figure S29** $^1$H NMR spectrum (DMSO-$d_6$, 500 MHz) of brasilterpene D (4) ...........16

**Figure S30** $^{13}$C NMR spectrum (DMSO-$d_6$, 100 MHz) of brasilterpene D (**4**) ..........17

**Figure S31** HSQC spectrum of brasilterpene D (**4**)...................................................18

**Figure S32** HMBC spectrum of brasilterpene D (**4**)..................................................18

**Figure S33** $^1$H-$^1$H COSY spectrum of brasilterpene D (**4**).........................................18

**Figure S34** NOESY spectrum of brasilterpene D (**4**).................................................19

**Figure S35** HRESIMS spectrum of brasilterpene E (**5**).............................................19
**Figure S36** IR spectrum (KBr) of brasilterpene E (**5**)................................................20
**Figure S37** $^{1}$H NMR spectrum (DMSO-$d_6$, 600 MHz) of brasilterpene E (**5**)...........21
**Figure S38** DEPTQ-NMR spectrum (DMSO-$d_6$, 100 MHz) of brasilterpene E (**5**)...21
**Figure S39** HSQC spectrum of brasilterpene E (**5**)..................................................22
**Figure S40** HMBC spectrum of brasilterpene E (**5**) .................................................22
**Figure S41** $^{1}$H-$^{1}$H COSY spectrum of brasilterpene E (**5**).........................................23
**Figure S42** NOESY spectrum of brasilterpene E (**5**).................................................23
**Figure S43** DFT-optimized structures for low-energy conformers of **1a** (1*S*,3*R*,5*R*,6*S*,9*S*,14*S*-**1**) at B3LYP/6-31+G(d) level in DMSO (PCM). .....................24
**Table S1** Harmonic frequencies (cm**$^{-1}$) of compound **1a**. .....................................25
**Table S2** Important thermodynamic parameters (a.u.) and Boltzmann distributions of the optimized compound **1a** at B3LYP/6-31G+(d) level of theory with PCM solvent model for DMSO. .........................................................................................25
**Table S3** Cartesian coordinates of the low-energy re-optimized conformers of compound **1a** (1*S*,3*R*,5*R*,6*S*,9*S*,14*S*-**1**) calculated at B3LYP/6-31+G(d) level of theory with PCM solvent model for DMSO. .........................................................................25
**Figure S44** DFT-optimized structures for low-energy conformers of **1b** (1*S*,3*S*,5*R*,6*S*,9*S*,14*S*-**1**) at B3LYP/6-31+G(d) level in DMSO (PCM).......................35
**Table S4** Harmonic frequencies (cm**$^{-1}$) of compound **1b**. .....................................35
**Table S5** Important thermodynamic parameters (a.u.) and Boltzmann distributions of the optimized compound **1b** at B3LYP/6-31G+(d) level of theory with PCM solvent model for DMSO. .........................................................................................36
**Table S6** Cartesian coordinates of the low-energy re-optimized conformers of compound **1b** (1*S*,3*S*,5*R*,6*S*,9*S*,14*S*-**1**) calculated at B3LYP/6-31+G(d) level of theory with PCM solvent model for DMSO. .........................................................................36
**Table S7** Calculated $^{1}$H and $^{13}$C NMR chemical shifts of compound **1a**. ..................43
**Table S8** Calculated $^{1}$H and $^{13}$C NMR chemical shifts of compound **1b**. ..................45
**Figure S45** Correlation plots of experimental $^{13}$C NMR chemical shifts of brasilterpene A (**1**) corresponding calculated $^{13}$C NMR chemical shifts of **1a** and **1b**. ...................47
**Table S9** DP4+ analysis using the calculated shielding tensors for compound **1a** and **1b**. .........................................................................................................48
**Figure S46** DFT-optimized structures for low-energy conformers of **1** (1*S*,3*S*,5*R*,6*S*,9*S*,14*S*-**1**) at B3LYP/6-31+G(d) level in MeOH (PCM). ......................48
**Table S10** Harmonic frequencies (cm**$^{-1}$) of compound **1**. .....................................49
**Table S11** Important thermodynamic parameters (a.u.) and Boltzmann distributions of the optimized compound **1** at B3LYP/6-31G+(d) level of theory with PCM solvent model for MeOH. .........................................................................................49

**Table S12** Cartesian coordinates of the low-energy re-optimized conformers of compound **1** (1*S*,3*S*,5*R*,6*S*,9*S*,14*S*-**1**) calculated at B3LYP/6-31+G(d) level of theory with PCM solvent model for MeOH......49

**Figure S47** DFT-optimized structures for low-energy conformers of **2** (1*S*,3*S*,5*R*,6*S*,9*S*,14*R*-**2**) at B3LYP/6-31+G(d) level in MeOH (PCM)......55

**Table S13** Harmonic frequencies (cm**$^{-1}$) of compound **2**. ......55

**Table S14** Important thermodynamic parameters (a.u.) and Boltzmann distributions of the optimized compound **2** at B3LYP/6-31G+(d) level of theory with PCM solvent model for MeOH......56

**Table S15** Cartesian coordinates of the low-energy re-optimized conformers of compound **2** (1*S*,3*S*,5*R*,6*S*,9*S*,14*R*-**2**) calculated at B3LYP/6-31+G(d) level of theory with PCM solvent model for MeOH......56

**Figure S48** DFT-optimized structures for low-energy conformers of **3** (1*S*,5*S*,6*S*,9*S*,14*S*-**3**) at B3LYP/6-31+G(d) level in MeOH (PCM). ......59

**Table S16** Harmonic frequencies (cm**$^{-1}$) of compound **3**. ......60

**Table S17** Important thermodynamic parameters (a.u.) and Boltzmann distributions of the optimized compound **3** at B3LYP/6-31G+(d) level of theory with PCM solvent model for MeOH......61

**Table S18** Cartesian coordinates of the low-energy re-optimized conformers of compound **3** (1*S*,5*S*,6*S*,9*S*,14*S*-**3**) calculated at B3LYP/6-31+G(d) level of theory with PCM solvent model for MeOH......61

**Figure S49** DFT-optimized structures for low-energy conformers of **4** (1*S*,5*S*,6*S*,9*S*,14*R*-**4**) at B3LYP/6-31+G(d) level in MeOH (PCM). ......67

**Table S19** Harmonic frequencies (cm**$^{-1}$) of compound **4**. ......67

**Table S20** Important thermodynamic parameters (a.u.) and Boltzmann distributions of the optimized compound **4** at B3LYP/6-31G+(d) level of theory with PCM solvent model for MeOH......67

**Table S21** Cartesian coordinates of the low-energy re-optimized conformers of compound **4** (1*S*,5*S*,6*S*,9*S*,14*R*-**4**) calculated at B3LYP/6-31+G(d) level of theory with PCM solvent model for MeOH......68

**Figure S50** DFT-optimized structures for low-energy conformers of **5** (1*S*,5*S*,6*S*,9*S*,14*R*-**5**) at B3LYP/tzvp level in MeOH (PCM). ......71

**Table S22** Harmonic frequencies (cm**$^{-1}$) of compound **5**. ......72

**Table S23** Important thermodynamic parameters (a.u.) and Boltzmann distributions of the optimized compound **5** at B3LYP/tzvp level of theory with PCM solvent model for MeOH. ......72

**Table S24** Cartesian coordinates of the low-energy re-optimized conformers of compound **5** (1*S*,5*S*,6*S*,9*S*,14*R*-**5**) calculated at B3LYP/tzvp level of theory with PCM solvent model for MeOH. ......72

**Figure S1** HPLC analysis of the crude extract of HDN15-135

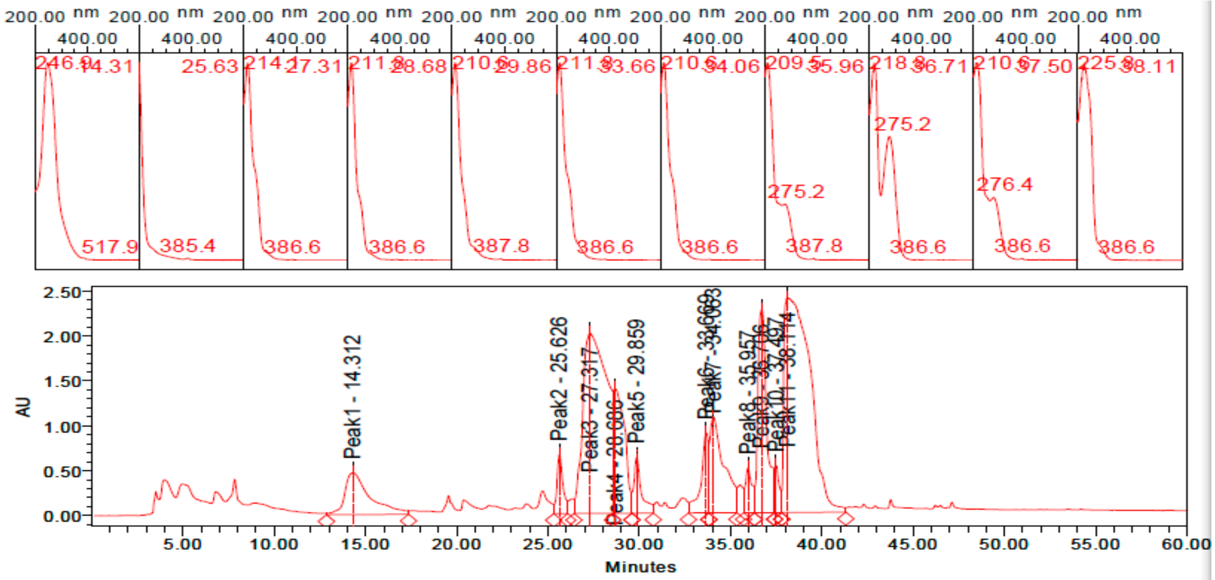


**Figure S2** Structures of reported bergamotane sesquiterpenoids

**Type I** bicyclo[3.1.1]heptan [1-9]

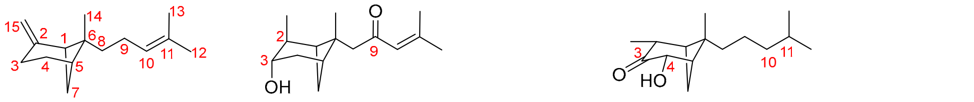

**1-1** beta-bergamotene [1] **1-2** tanavulgarol [2] **1-3** 3beta-hydroxy-2-oxo1,2,12,1 3-tetrahydro-bergamotene [3]

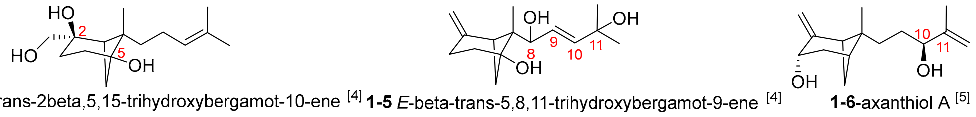

**1-4** beta-trans-2beta,5,15-trihydroxybergamot-10-ene [4] **1-5** *E*-beta-trans-5,8,11-trihydroxybergamot-9-ene [4] **1-6**-axanthiol A [5]

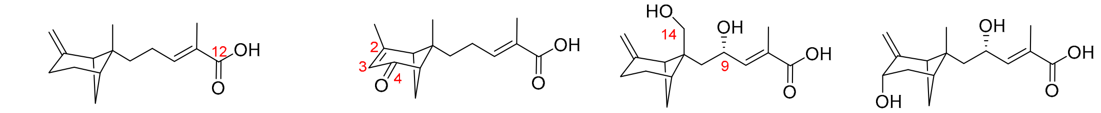

**1-7** (+)-(E)-endo-beta-bergamotenoic acid [6] **1-8** schisanpropinoic acid [7] **1-9** massarinolin C [8] **1-10** donacinoic acid B [9]

**Type II** tricyclo[4.3.0.0$^{4,7}$]nonane [9-14]

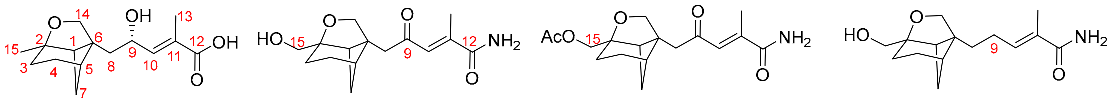

**2-1** massarinolin B [9] **2-2** brasilamide C [10] **2-3** brasilamide D [10] **2-4** brasilamide L [11]

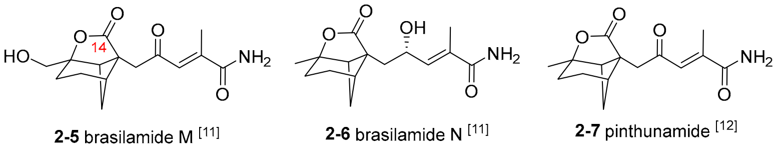

**2-5** brasilamide M [11] **2-6** brasilamide N [11] **2-7** pinthunamide [12]

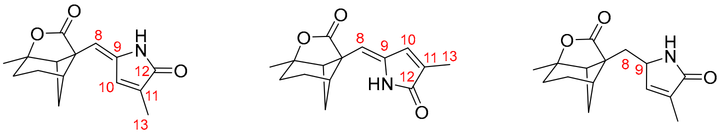

**2-8** ampullicin [13] **2-9** isoampullicin [13] **2-10** dihydroampullicin [14]

## **Type III** tricyclo[3.3.1.0$^{2,7}$]nonane [10, 11]

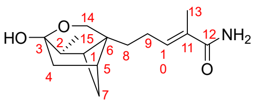

**3-1** brasilamide B [10]

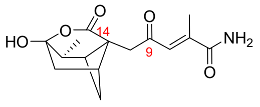

**3-2** brasilamide A [10]

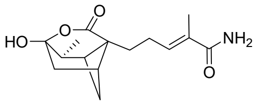

**3-3** brasilamide K [11]

## **Type IV** 6/4/5/5 tetracyclic skeleton [8, 9, 15-17]

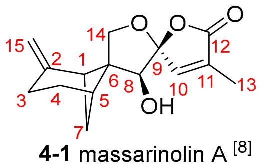

**4-1** massarinolin A [8]

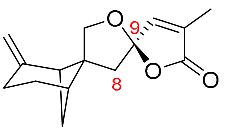

**4-2** donacinolide A [9]

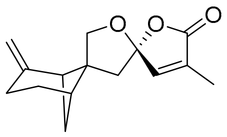

**4-3** donacinolide B [9]

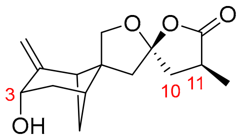

**4-4** expansolide C [15]

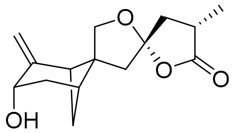

**4-5** expansolide D [15]

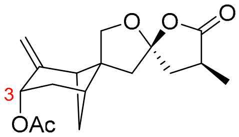

**4-6** expansolide A [16]

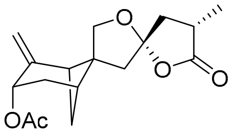

**4-7** expansolide B [16]

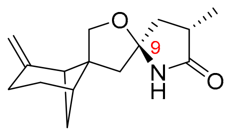

**4-8** sporulaminal A [17]

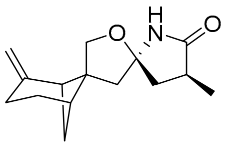

**4-9** sporulaminal B [17]

## **Type V** 5/6/4/5 tetracyclic skeleton [9]

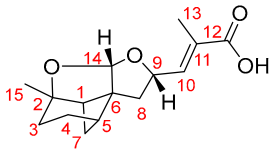

**5-1** donacinoic acid A [9]

## References

[1] Kulkarni, K. S.; Paknikar, S. K.; Vaidya, A. S.; Kelkar, G. R.; Bates, R. B.; Bhattacharyya, S. C. Structure of *β*-bergamotene. *Tetrahedron Lett.* **1963**, *4*, 505-511. https://doi.org/10.1016/S0040-4039(01)90662-8.

[2] Chandra, A.; Misra, L. N.; Thakur, R. S. Tanavulgarol, an oxygenated sesquiterpene with an uncommon skeleton from *Tanacetum vulgare*. *Phytochemistry* **1987**, *26*, 3077-3078. https://doi.org/10.1016/S0031-9422(00)84600-2.

[3] Misra, L. N.; Ahmad, A. An oxygenated tetrahydrobergamotene from the essential oil of *Dracocephalum nutans*. *Planta Med.* **1992**, *58*, 478-479. https://doi.org/10.1055/s-2006-961524.

[4] Wang, Y.; Li, D. H.; Li, Z. L.; Sun, Y. J.; Hua, H. M.; Liu, T.; Bai, J. Terpenoids from the marine-derived fungus *Aspergillus fumigatus* YK-7. *Molecules* **2016**, *21*, 31. https://doi.org/10.3390/molecules21010031.

[5] Yin, H.; Jiang, N.; Guo, C.; Gao, J.-M. A new bergamotane sesquiterpenoid from the rhizomes of *Amomum villosum* var. *xanthioides*. *Nat. Prod. Res.* **2021**, *35*, 377-383. https://doi.org/10.1080/14786419.2019.1633649.

[6] Breeden, D. C.; Young, T. E.; Coates, R. M.; Juvik, J. A. Identification and bioassay of kairomones for *Helicoverpa zea*. *J. Chem. Ecol.* **1996**, *22*, 513-539. https://doi.org/10.1007/BF02033653.
[7] Liu, M.; Hu, Z. X.; Luo, Y. Q.; Zhou, M.; Wang, W. G.; Li, X. N.; Du, X.; Pu, J. X.; Sun, H. D. Two new compounds from *Schisandra propinqua* var. *propinqua*. *Nat. Prod. Bioprospect.* **2017**, *7*, 257-262. https://doi.org/10.1007/s13659-017-0129-7.
[8] Oh, H.; Gloer, J. B.; Shearer, C. A. Massarinolins A-C: new bioactive sesquiterpenoids from the aquatic fungus *Massarina tunicata*. *J. Nat. Prod.* **1999**, *62*, 497-501. https://doi.org/10.1021/np980447+.
[9] Zhao, Z. Z.; Zhao, K.; Chen, H. P.; Bai, X.; Zhang, L.; Liu, J. K. Terpenoids from the mushroom-associated fungus *Montagnula donacina*. *Phytochemistry* **2018**, *147*, 21-29. https://doi.org/10.1016/j.phytochem.2017.12.015.
[10] Liu, L.; Gao, H.; Chen, X.; Cai, X.; Yang, L.; Guo, L.; Yao, X.; Che, Y. Brasilamides A-D: sesquiterpenoids from the plant endophytic fungus *Paraconiothyrium brasiliense*. *Eur. J. Org. Chem.* **2010**, *2010*, 3302-3306. https://doi.org/10.1002/ejoc.201000284.
[11] Guo, Z.; Ren, F.; Che, Y.; Liu, G.; Liu, L. New bergamotane sesquiterpenoids from the plant endophytic fungus *Paraconiothyrium brasiliense*. *Molecules* **2015**, *20*, 14611-14620. https://doi.org/10.3390/molecules200814611.
[12] Kimura, Y.; Nakajima, H.; Hamasaki, T. Pinthunamide, a new tricyclic sesquiterpene amide produced by a fungus, *Ampullifernia* sp. *Tetrahedron Lett.* **1989**, *30*, 1267-1270. https://doi.org/10.1016/S0040-4039(00)72732-8.
[13] Kimura, Y.; Nakajima, H.; Hamasaki, T.; Matsumoto, T.; Matsuda, Y.; Tsuneda, A. Ampullicin and isoampullicin, new metabolites from an *Ampulliferina*-like fungus sp. No. 27. *Agric. Biol. Chem.* **1990**, *54*, 813-814. https://doi.org/10.1271/bbb1961.54.813.
[14] Kimura, Y.; Matsumoto, T.; Nakajima, H.; Hamasaki, T.; Matsuda, Y. Dihydroampullicin, a new plant growth regulators produced by the *Ampulliferina*-like fungus sp. No. 27. *Biosci. Biotechnol. Biochem.* **1993**, *57*, 687-688. https://doi.org/10.1271/bbb.57.687.
[15] Ying, Y. M.; Fang, C. A.; Yao, F. Q.; Yu, Y.; Shen, Y.; Hou, Z. N.; Wang, Z.; Zhang, W.; Shan, W. G.; Zhan, Z. J. Bergamotane sesquiterpenes with alpha-glucosidase inhibitory activity from the plant pathogenic fungus *Penicillium expansum*. *Chem. Biodivers.* **2017**, *14*, e1600184. https://doi.org/10.1002/cbdv.201600184.
[16] Massias, M.; Rebuffat, S.; Molho, L.; Chiaroni, A.; Riche, C.; Bodo, B. Expansolides A and B: tetracyclic sesquiterpene lactones from *Penicillium expansum*. *J. Am. Chem. Soc.* **1990**, *112*, 8112-8115. https://doi.org/10.1021/ja00178a039.
[17] Zhang, L. H.; Feng, B. M.; Chen, G.; Li, S. G.; Sun, Y.; Wu, H. H.; Bai, J.; Hua, H. M.; Wang, H. F.; Pei, Y. H. Sporulaminals A and B: a pair of unusual epimeric spiroaminal derivatives from a marine-derived fungus *Paraconiothyrium sporulosum* YK-03. *RSC Adv.* **2016**, *6*, 42361-42366. https://doi.org/10.1039/c6ra01401a.

**Figure S3** HRESIMS spectrum of brasilterpene A (**1**)

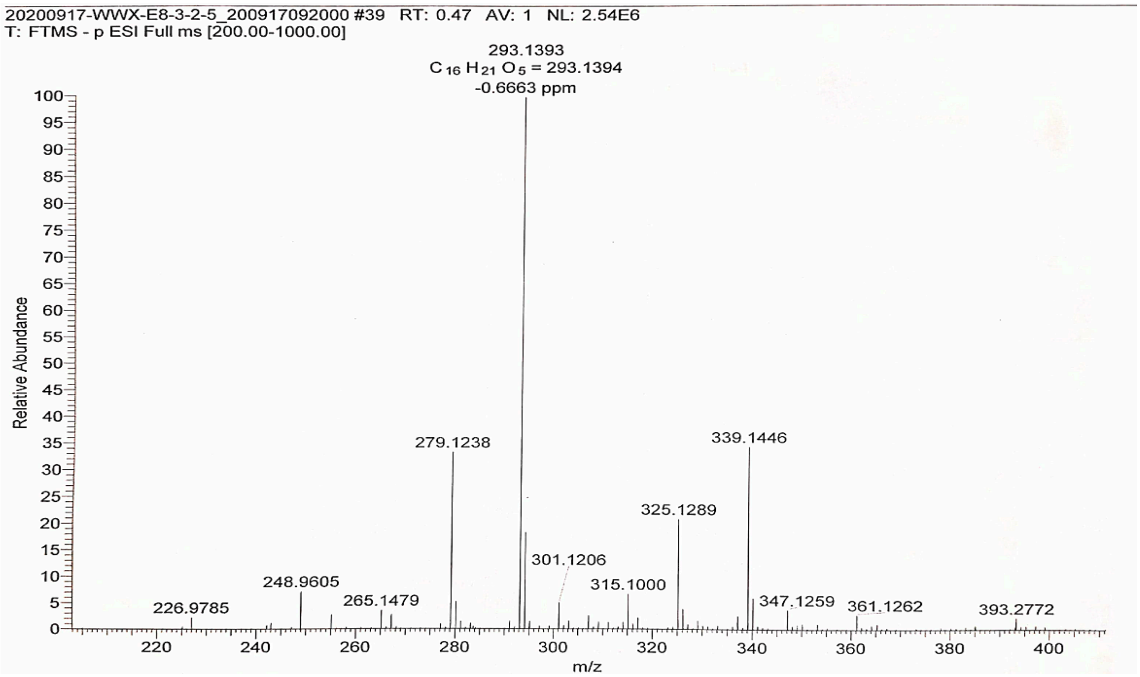


**Figure S4** IR spectrum (KBr) of brasilterpene A (**1**)

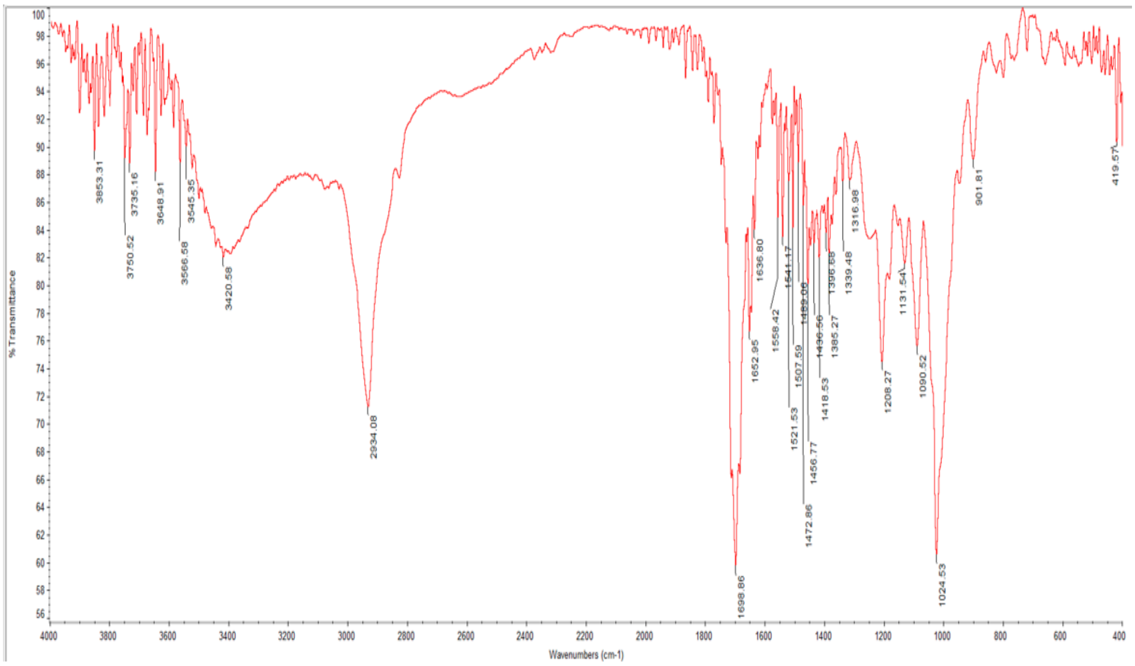

**Figure S5** $^{1}$H NMR spectrum (DMSO-$d_6$, 400 MHz) of brasilterpene A (**1**)

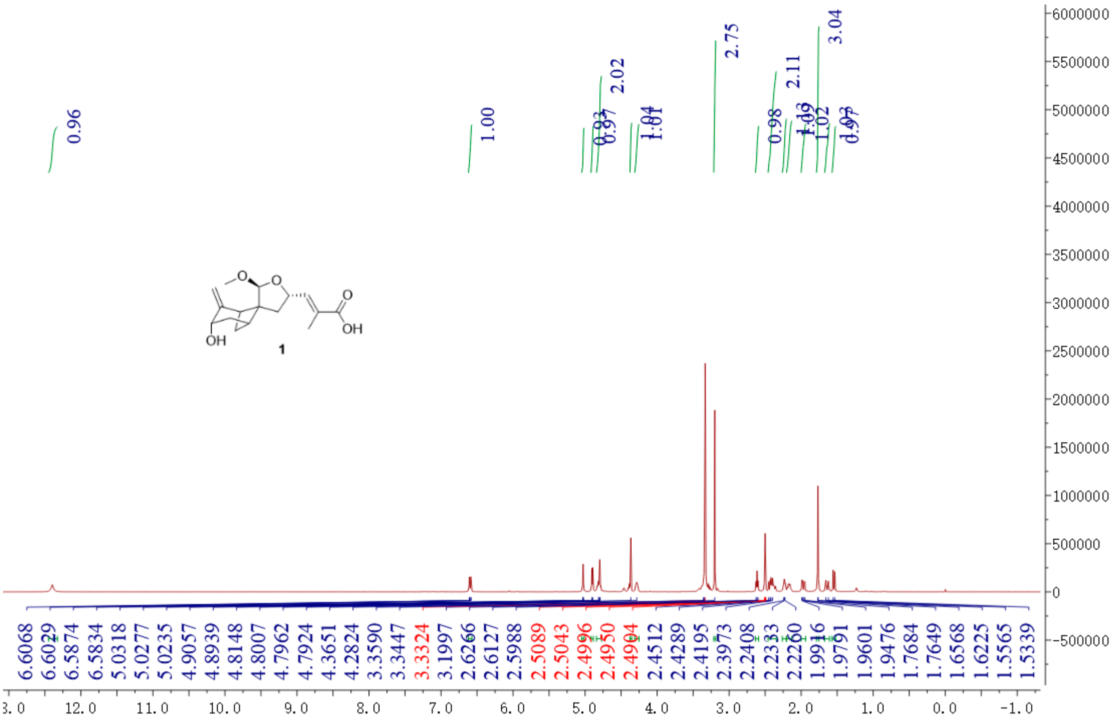

**Figure S6** DEPTQ-NMR spectrum (DMSO-$d_6$, 100 MHz) of brasilterpene A (**1**)

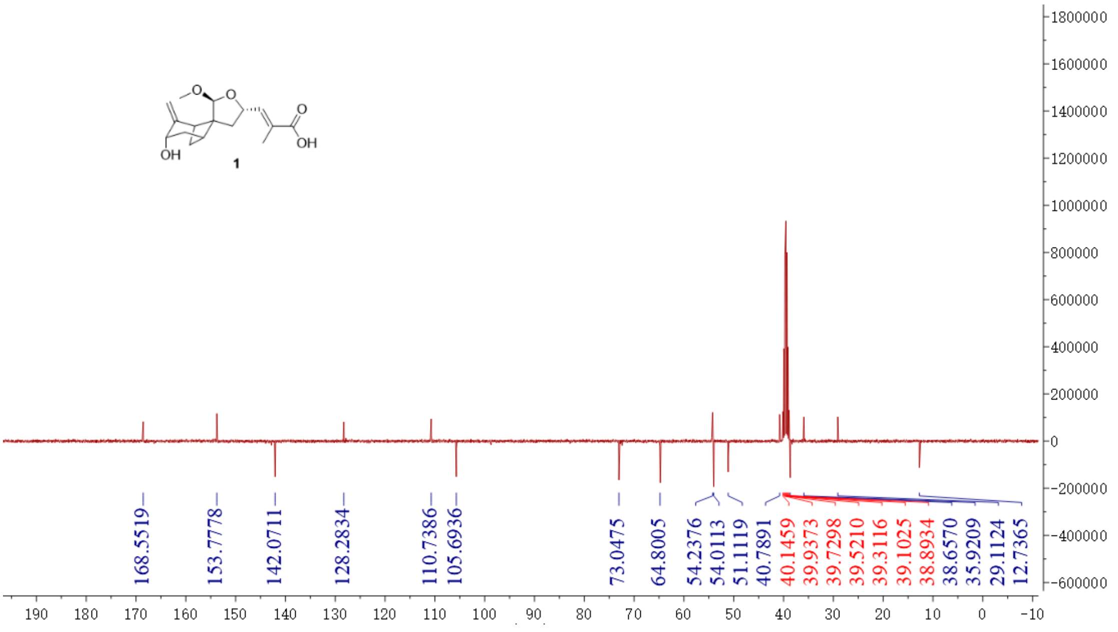

**Figure S7** HSQC spectrum of brasilterpene A (**1**)

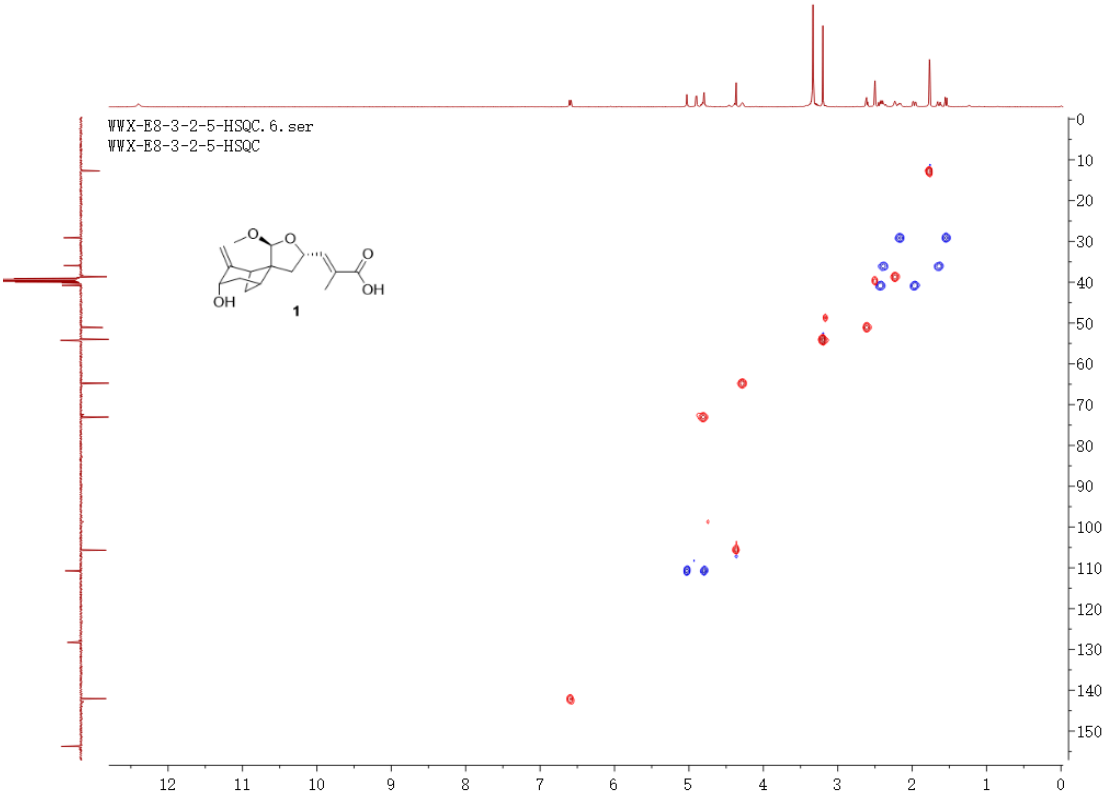


**Figure S8** HMBC spectrum of brasilterpene A (**1**)

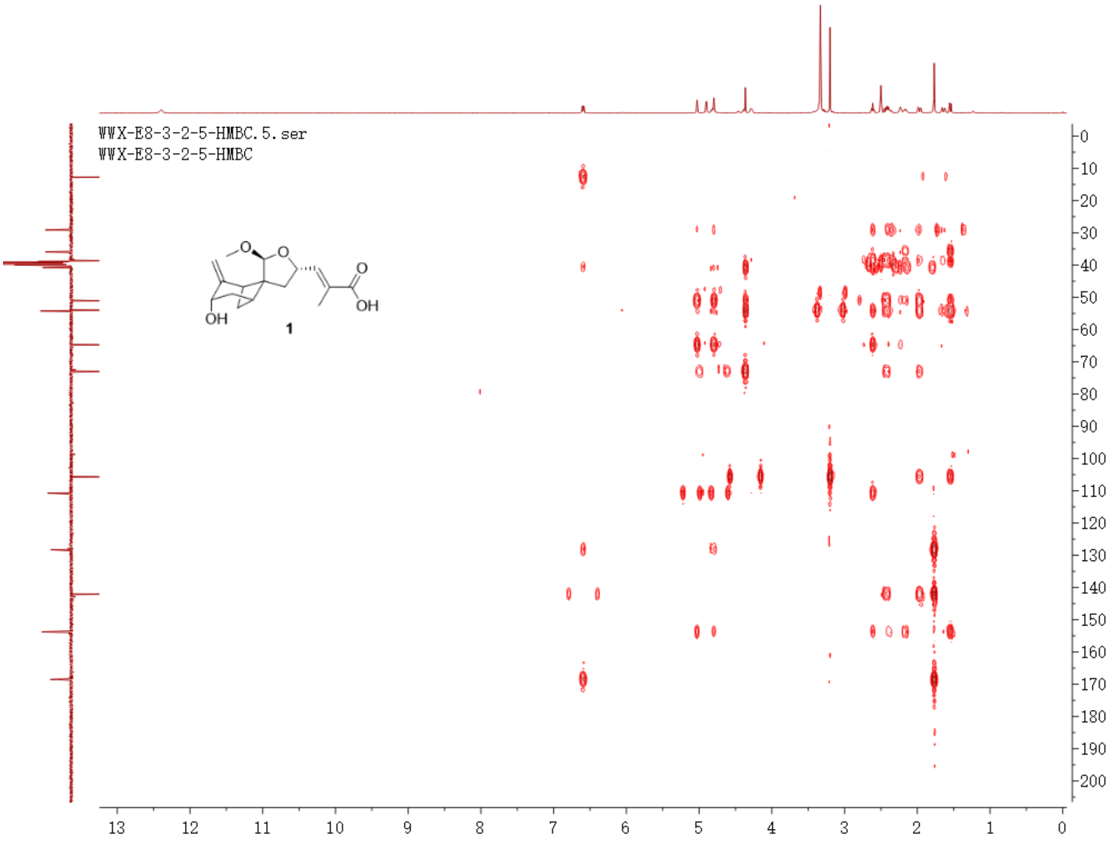

**Figure S9** $^{1}H$-$^{1}H$ COSY spectrum of brasilterpene A (**1**)

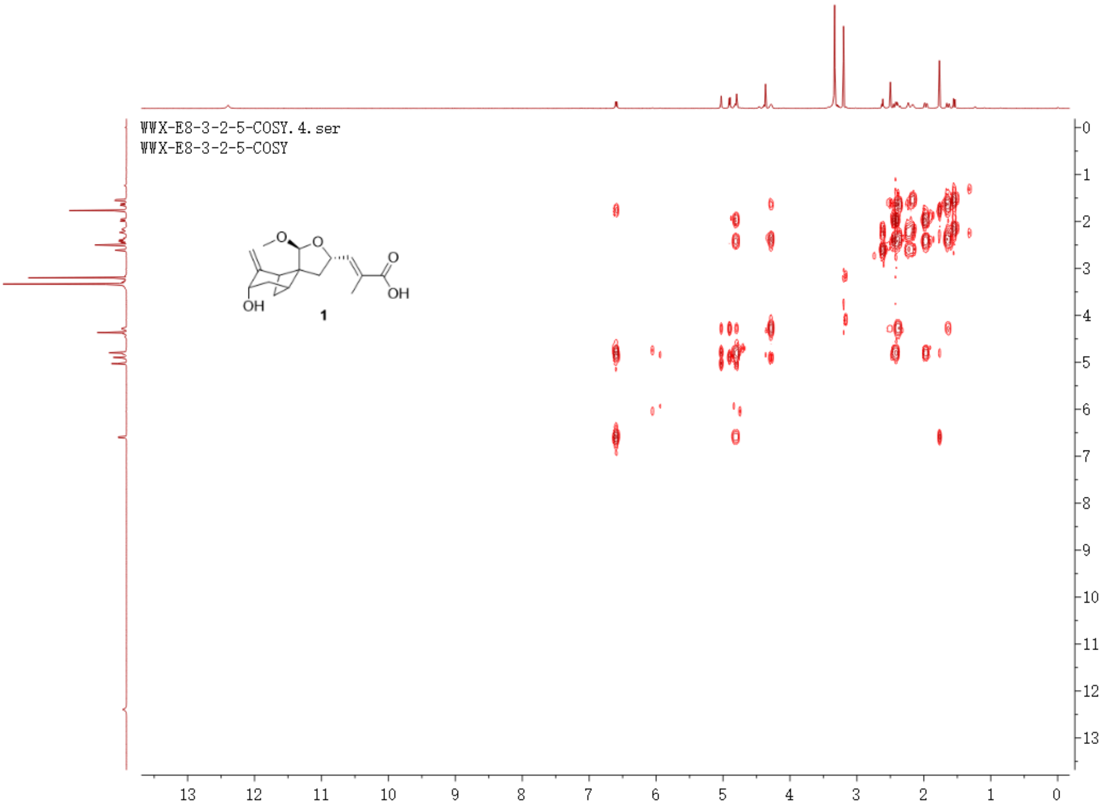


**Figure S10** NOESY spectrum of brasilterpene A (**1**)

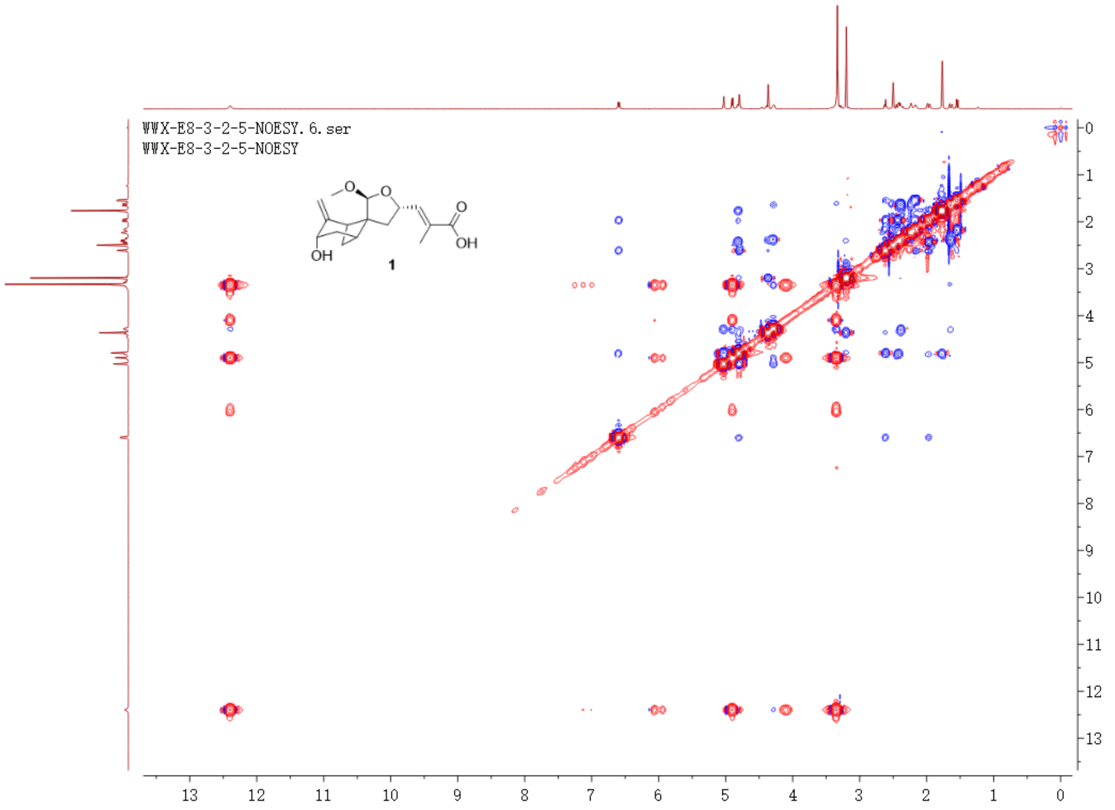

**Figure S11** HRESIMS spectrum of brasilterpene B (**2**)

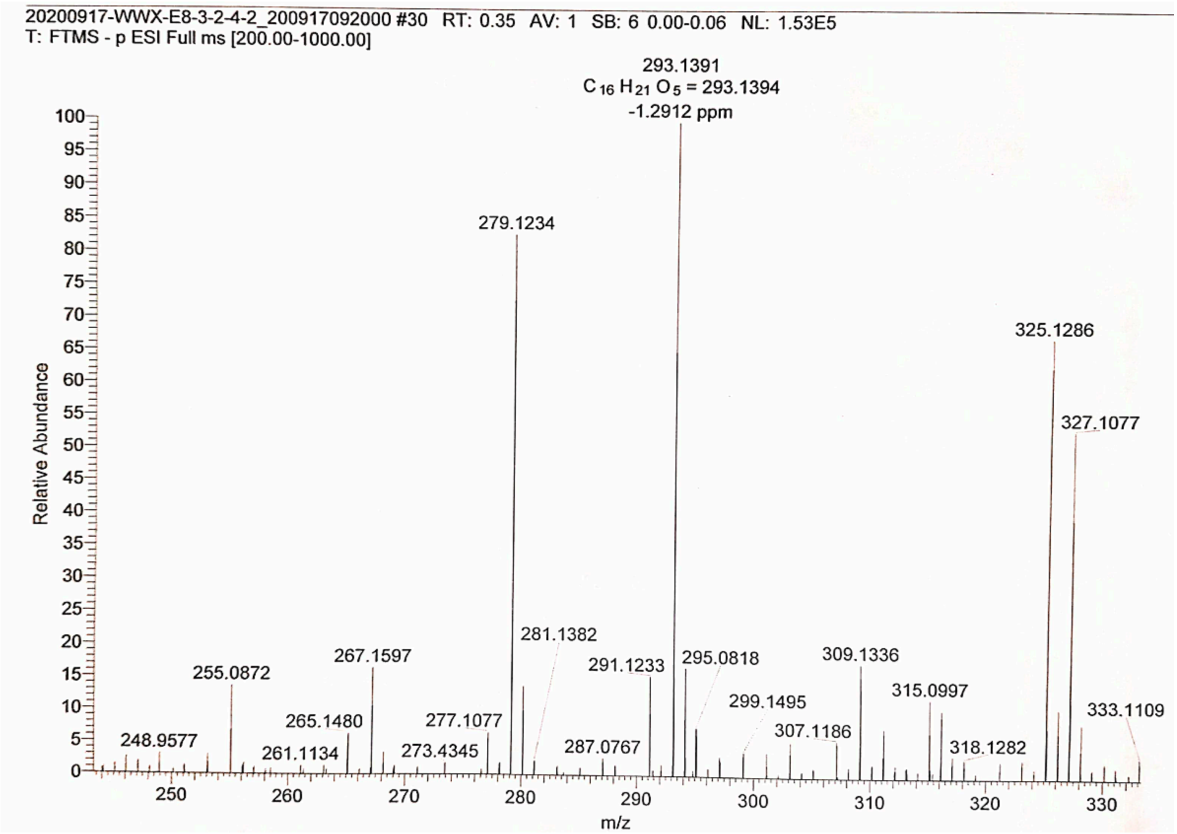


**Figure S12** IR spectrum (KBr) of brasilterpene B (**2**)

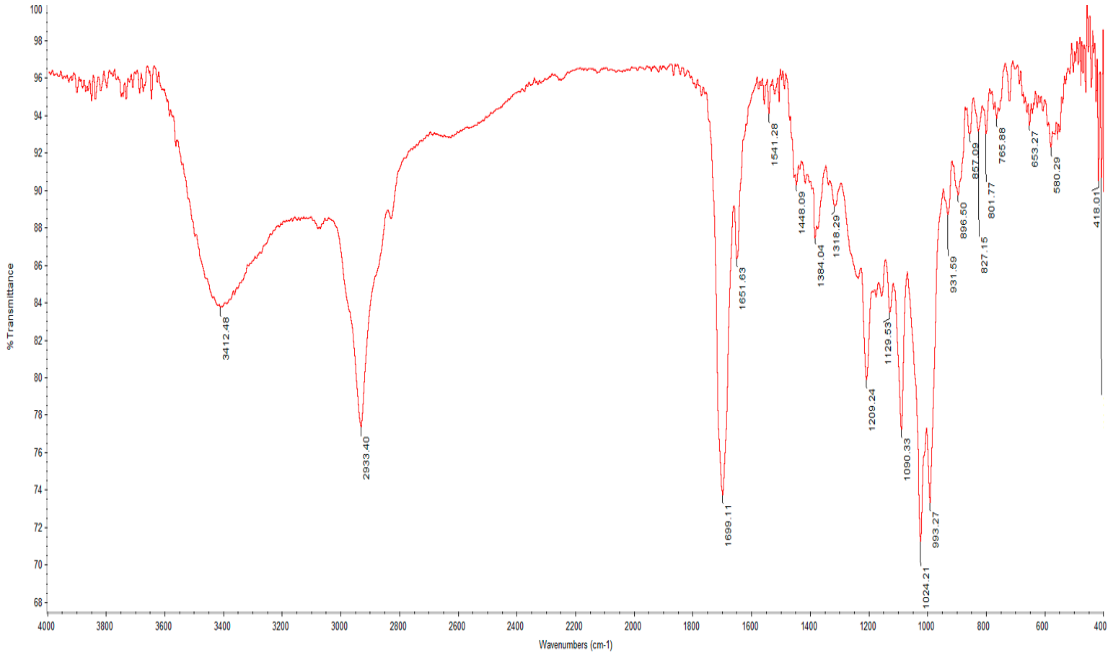

**Figure S13** $^{1}$H NMR spectrum (DMSO-$d_6$, 400 MHz) of brasilterpene B (**2**)

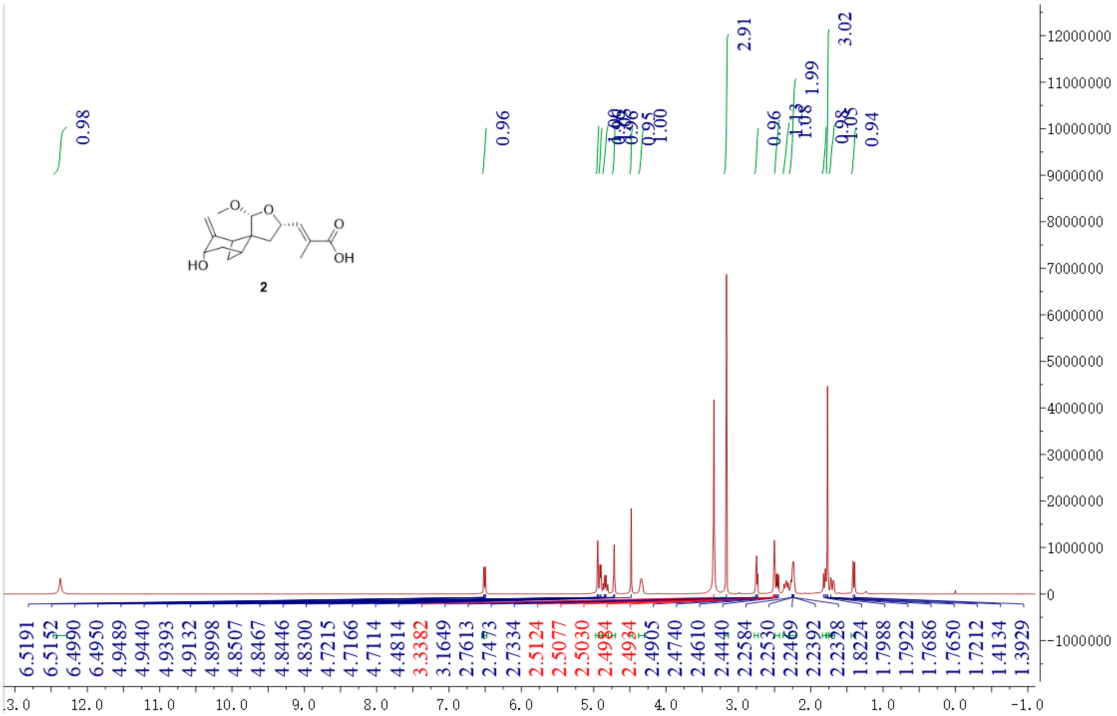

**Figure S14** DEPTQ-NMR spectrum (DMSO-$d_6$, 125 MHz) of brasilterpene B (**2**)

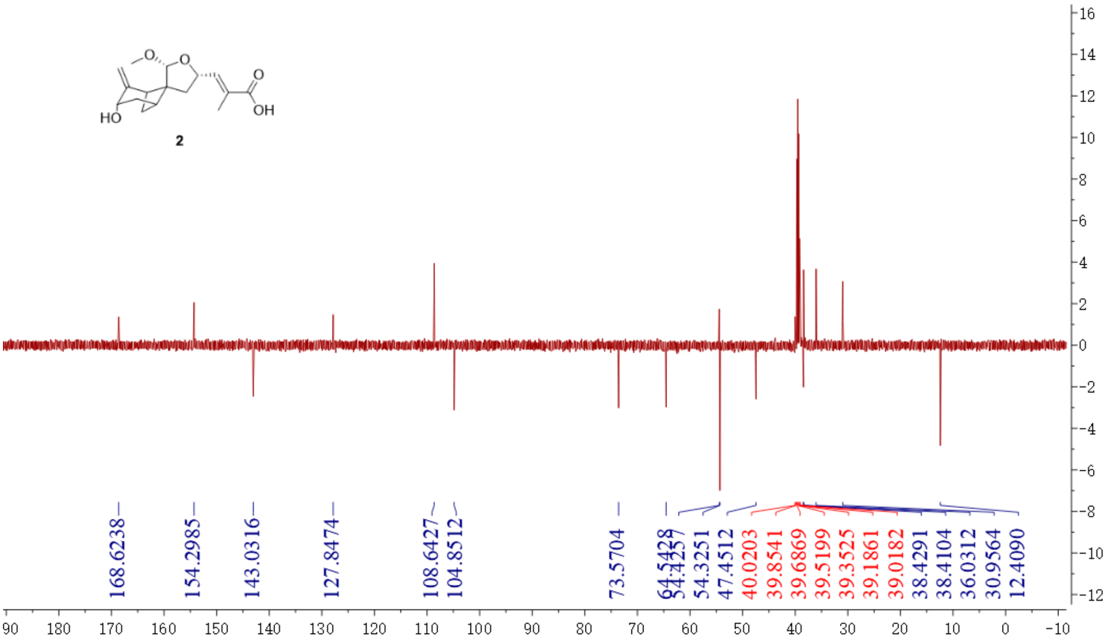

**Figure S15** HSQC spectrum of brasilterpene B (**2**)

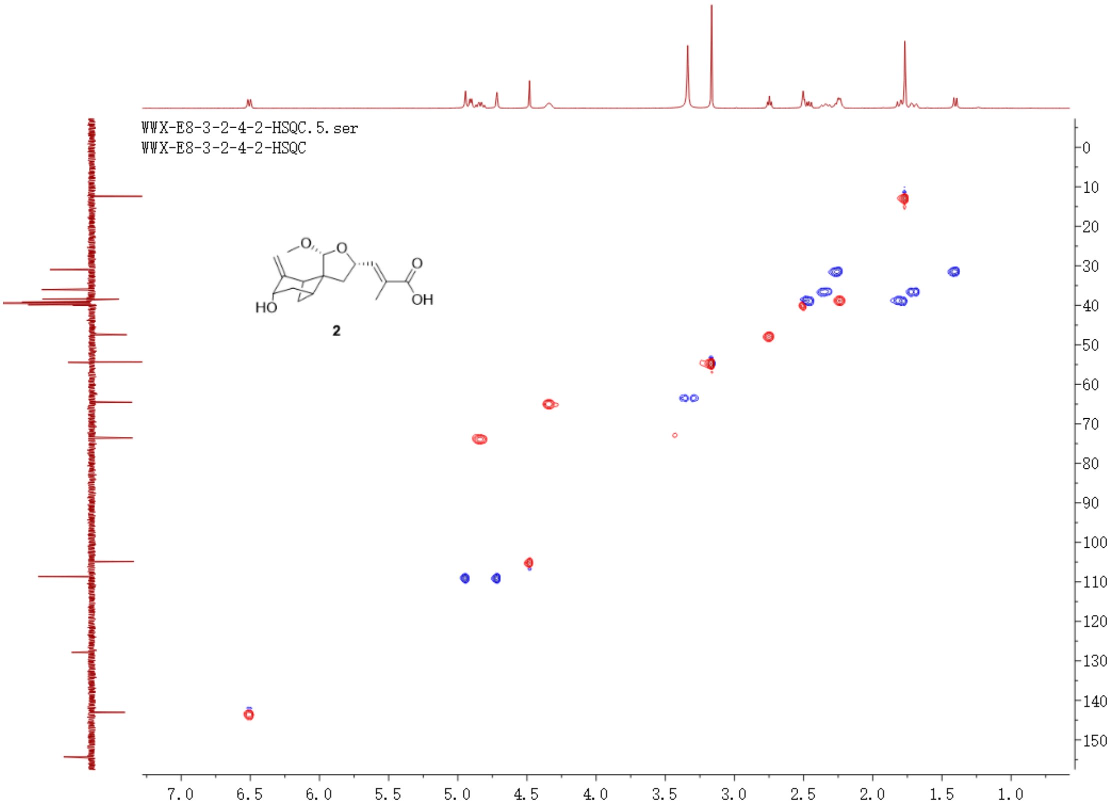


**Figure S16** HMBC spectrum of brasilterpene B (**2**)

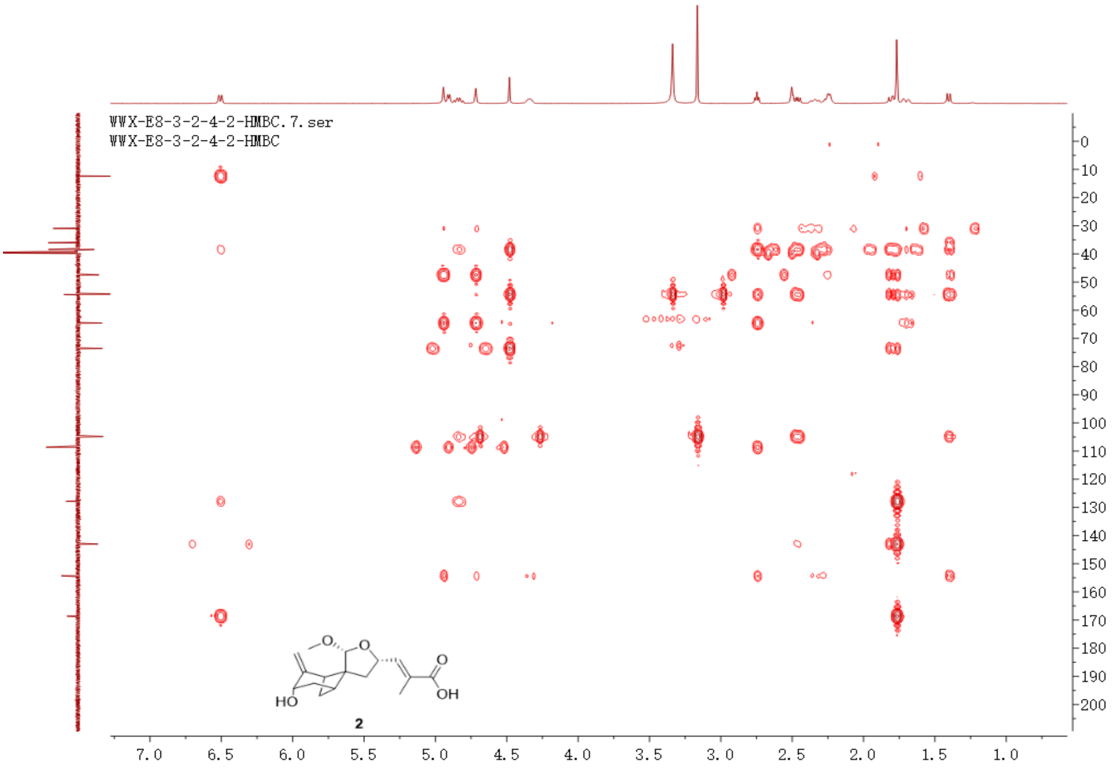

**Figure S17** $^{1}$H-$^{1}$H COSY spectrum of brasilterpene B (**2**)

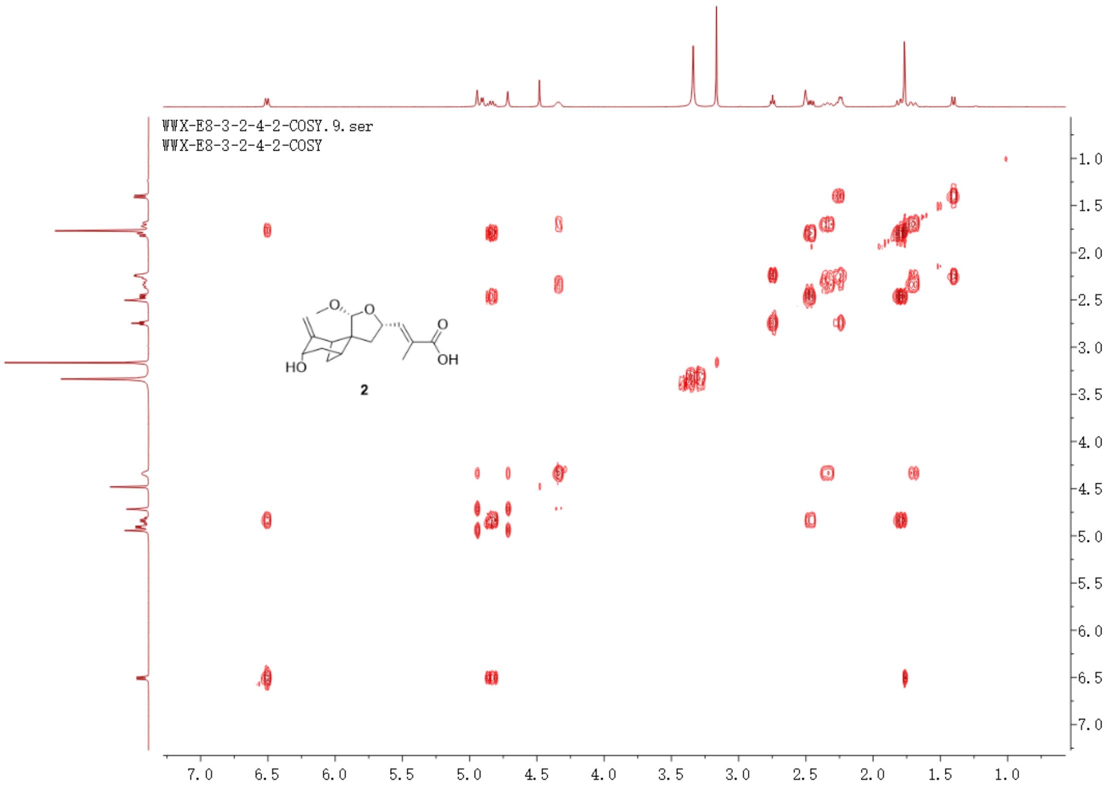


**Figure S18** NOESY spectrum of brasilterpene B (**2**)

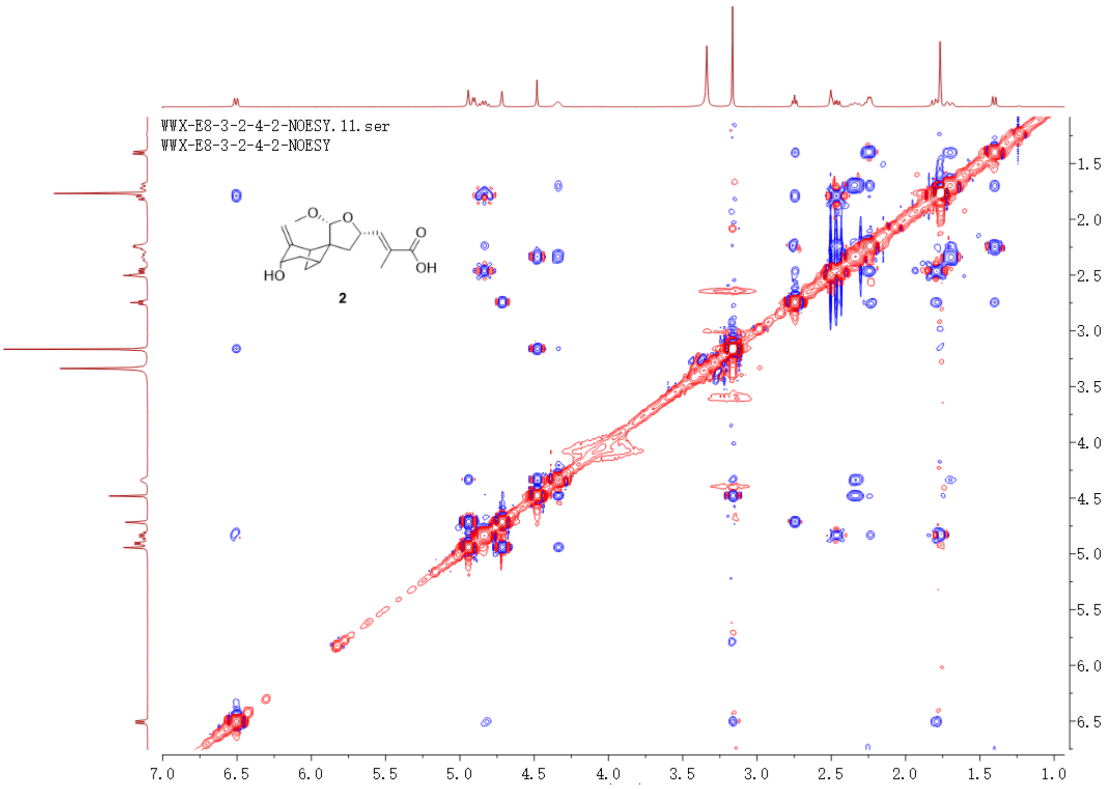

**Figure S19** HRESIMS spectrum of brasilterpene C (**3**)

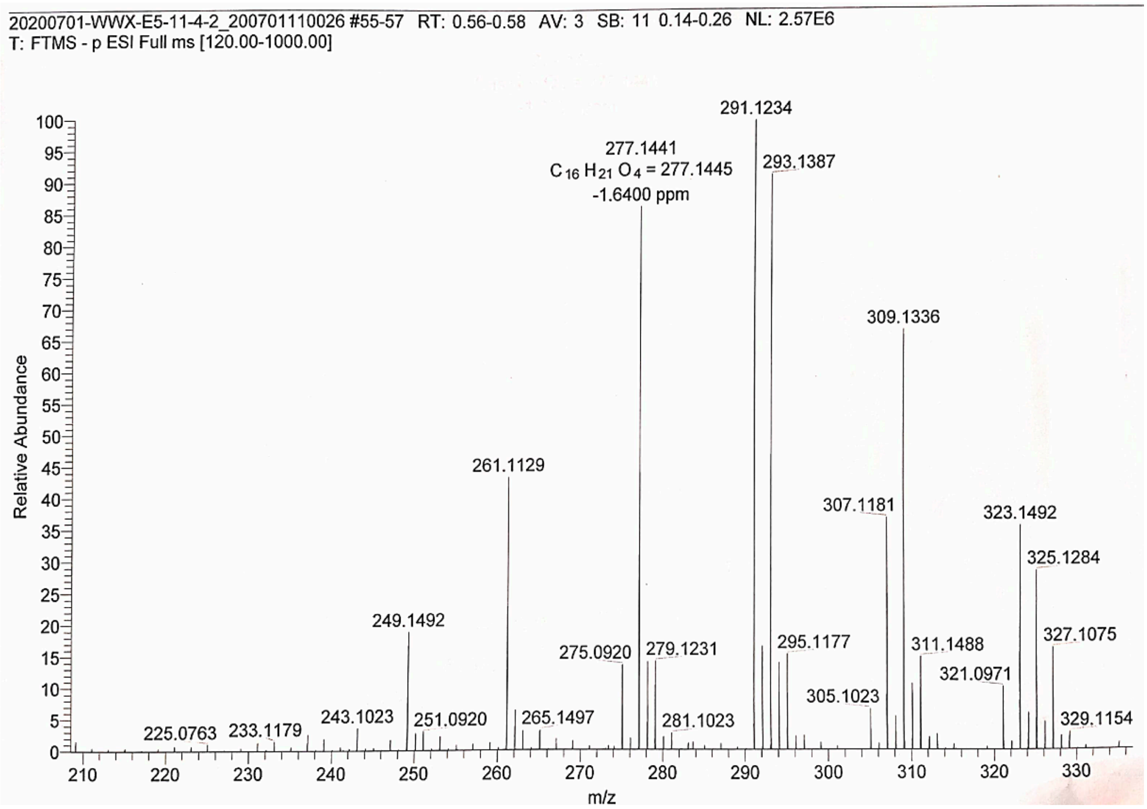


**Figure S20** IR spectrum (KBr) of brasilterpene C (**3**)

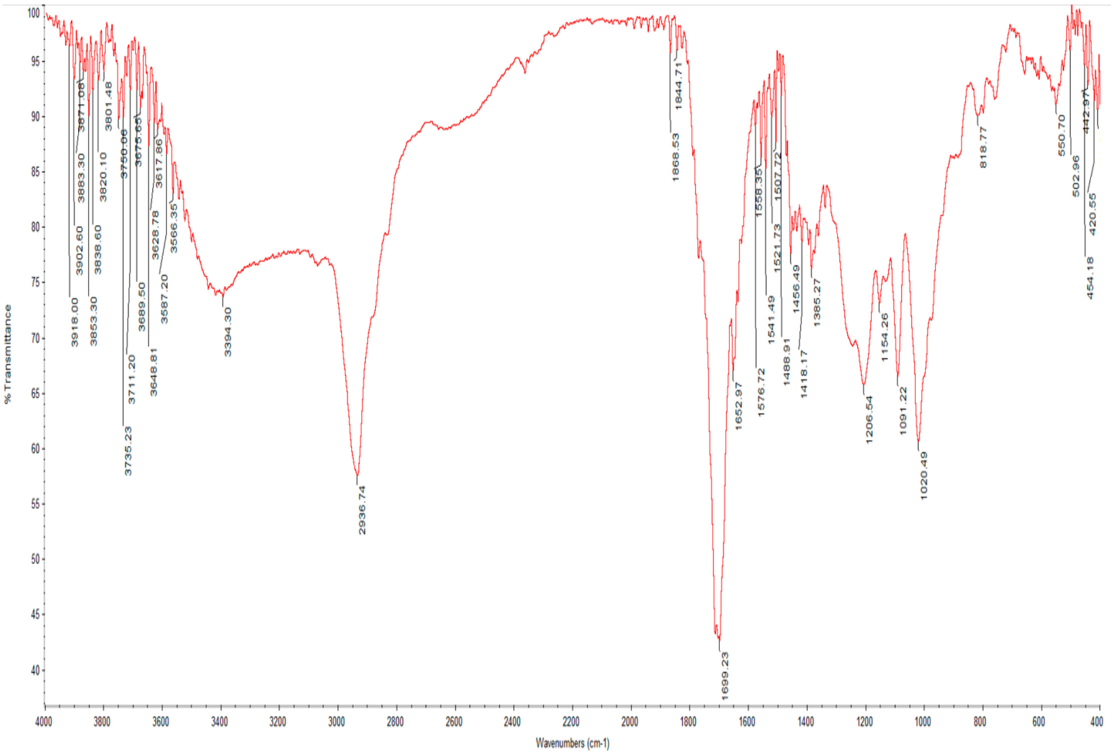

**Figure S21** $^{1}$H NMR spectrum (DMSO-$d_6$, 500 MHz) of brasilterpene C (**3**)

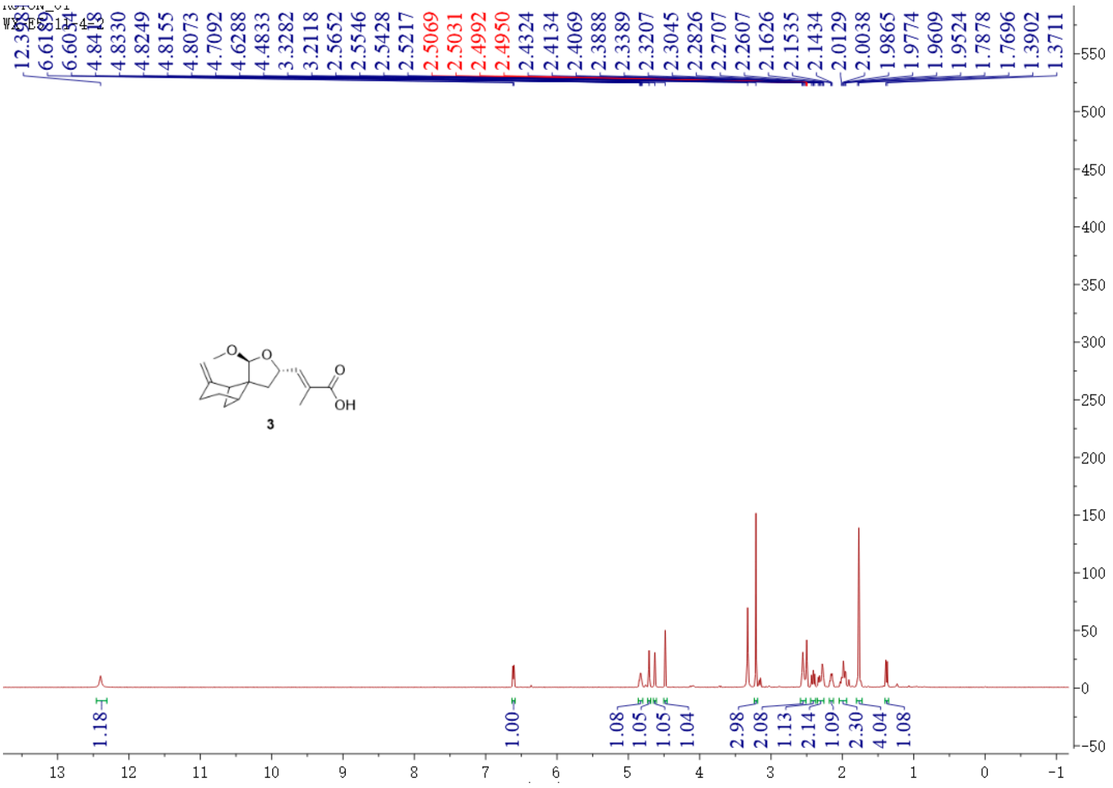


**Figure S22** $^{13}$C NMR spectrum (DMSO-$d_6$, 100 MHz) of brasilterpene C (**3**)

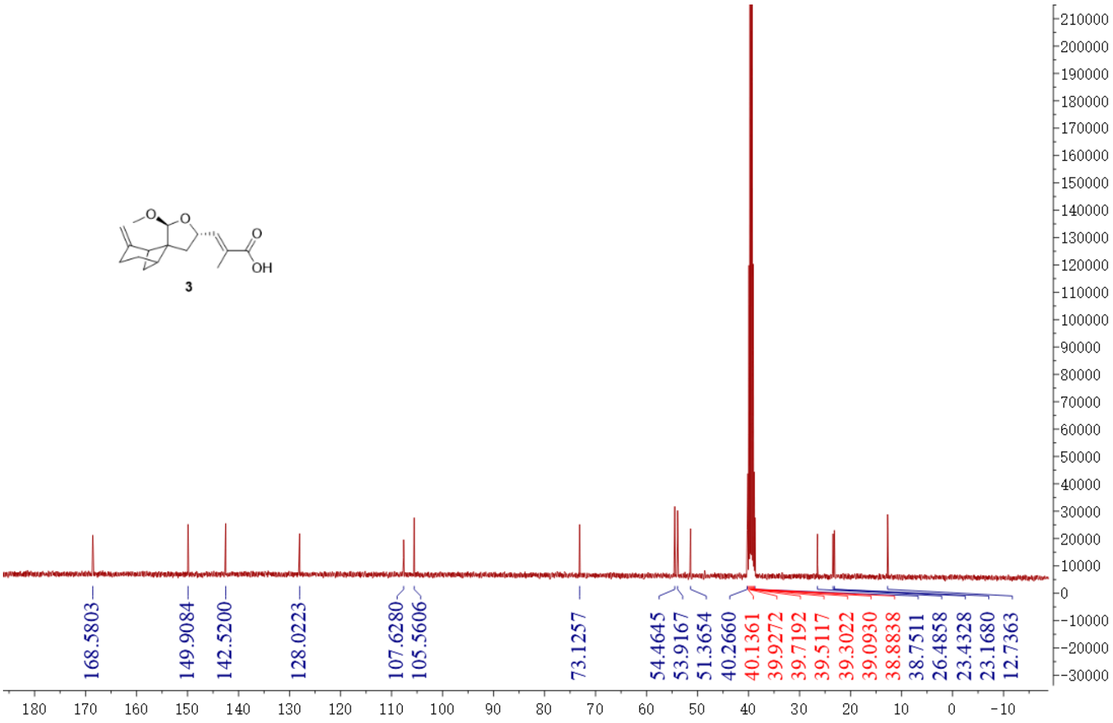

**Figure S23** HSQC spectrum of brasilterpene C (**3**)

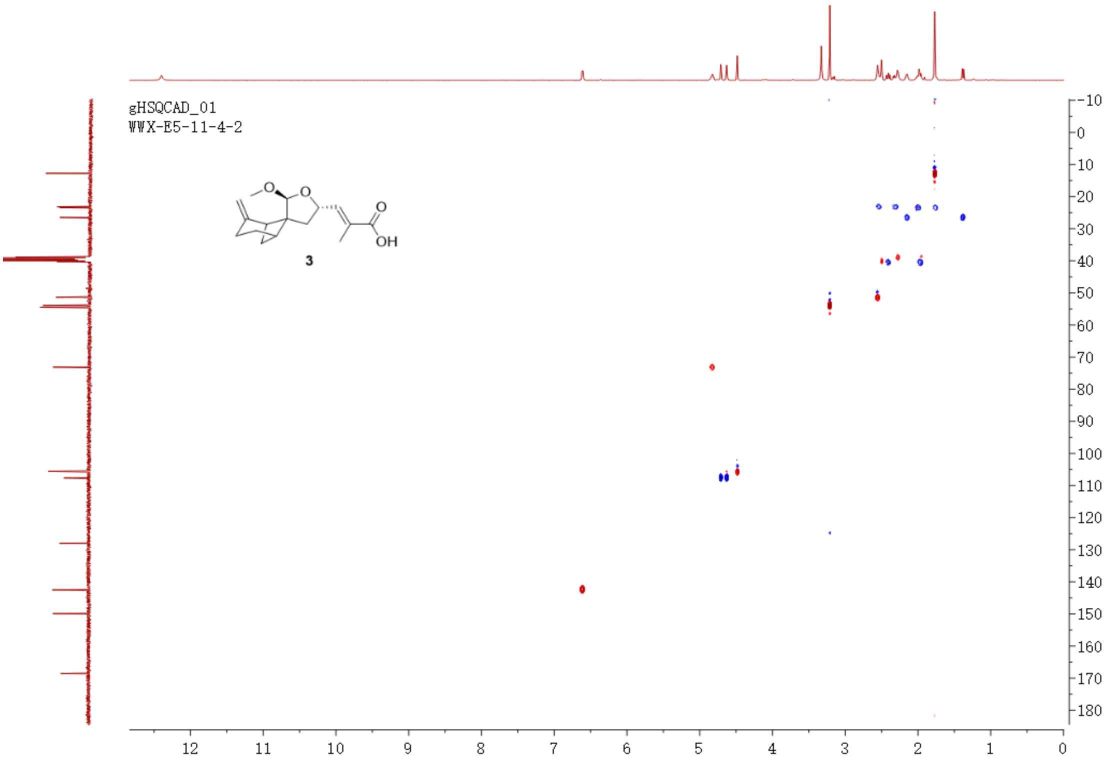


**Figure S24** HMBC spectrum of brasilterpene C (**3**)

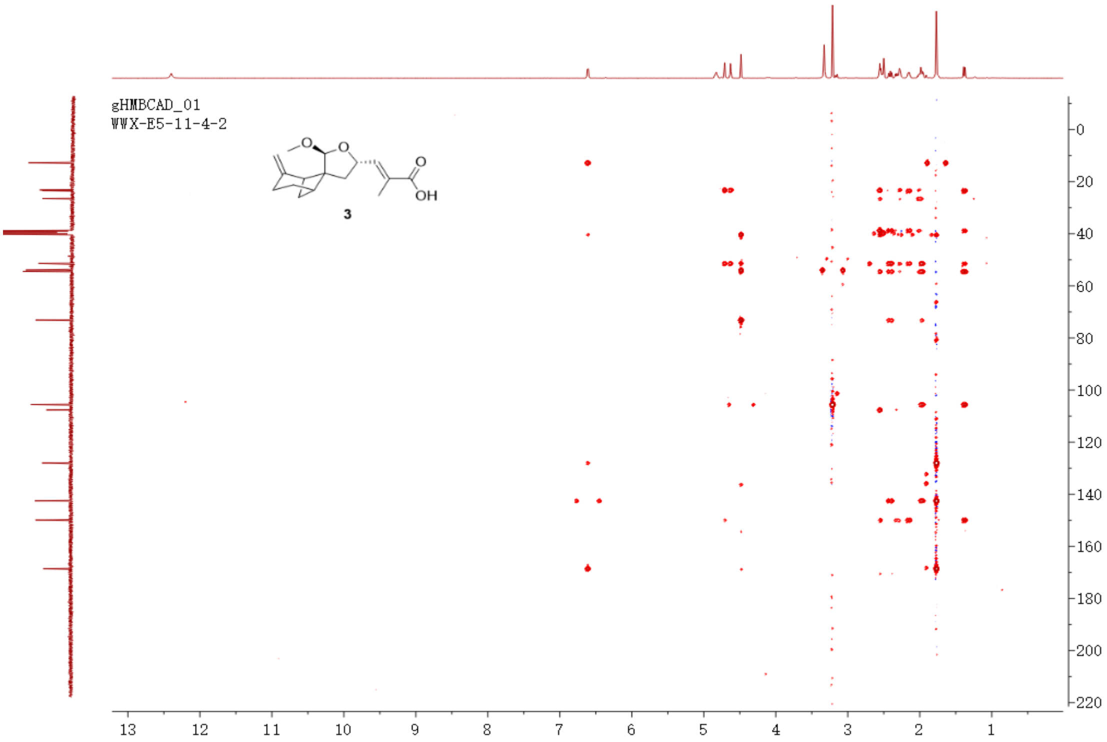


**Figure S25** $^{1}H$-$^{1}H$ COSY spectrum of brasilterpene C (**3**)

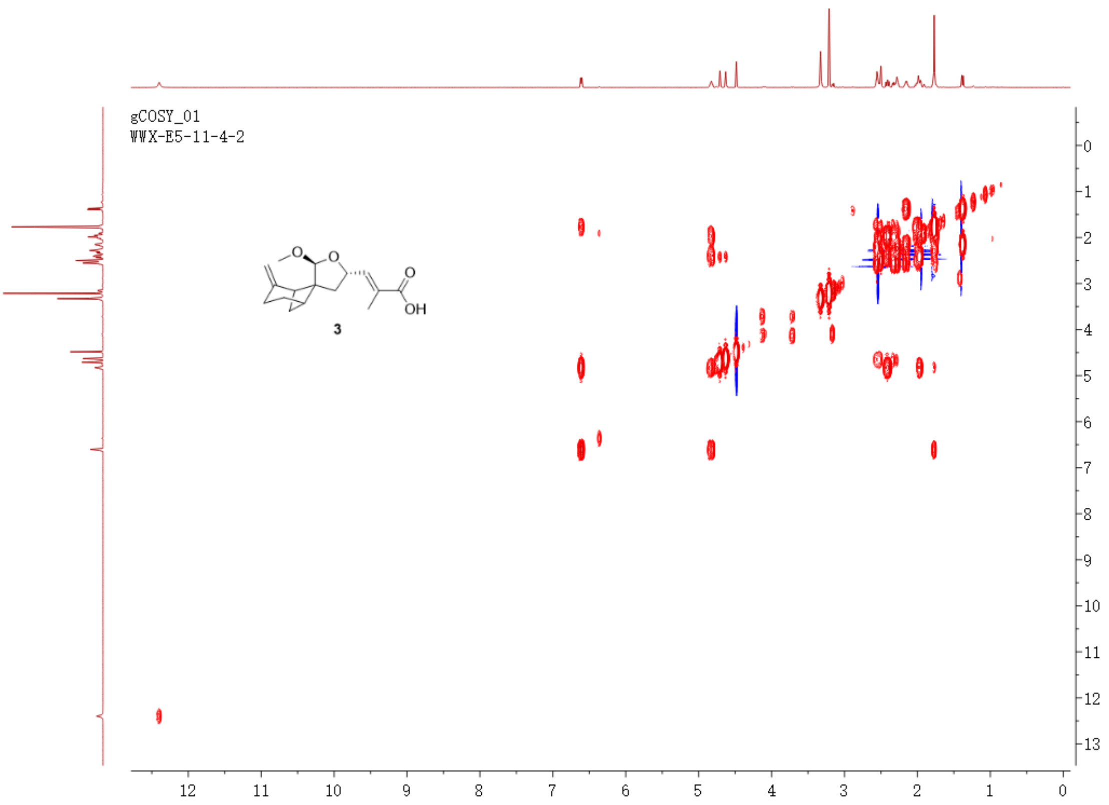


**Figure S26** NOESY spectrum of brasilterpene C (**3**)

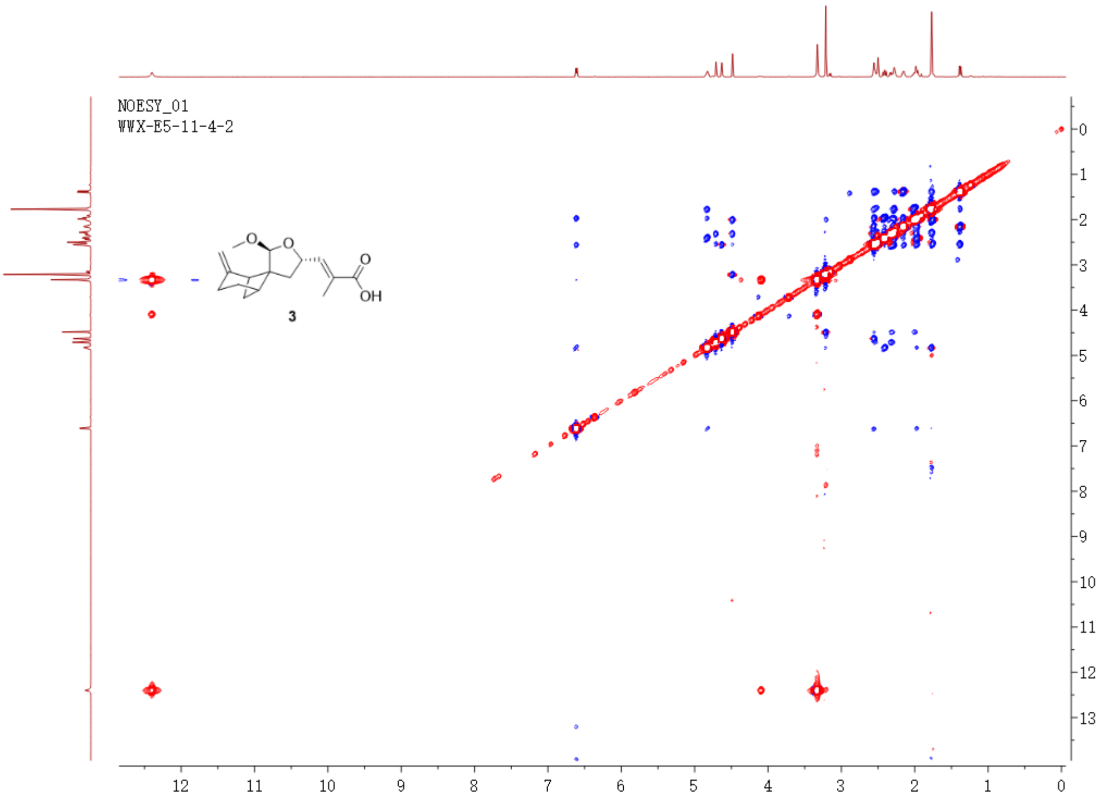


**Figure S27** HRESIMS spectrum of brasilterpene D (**4**)

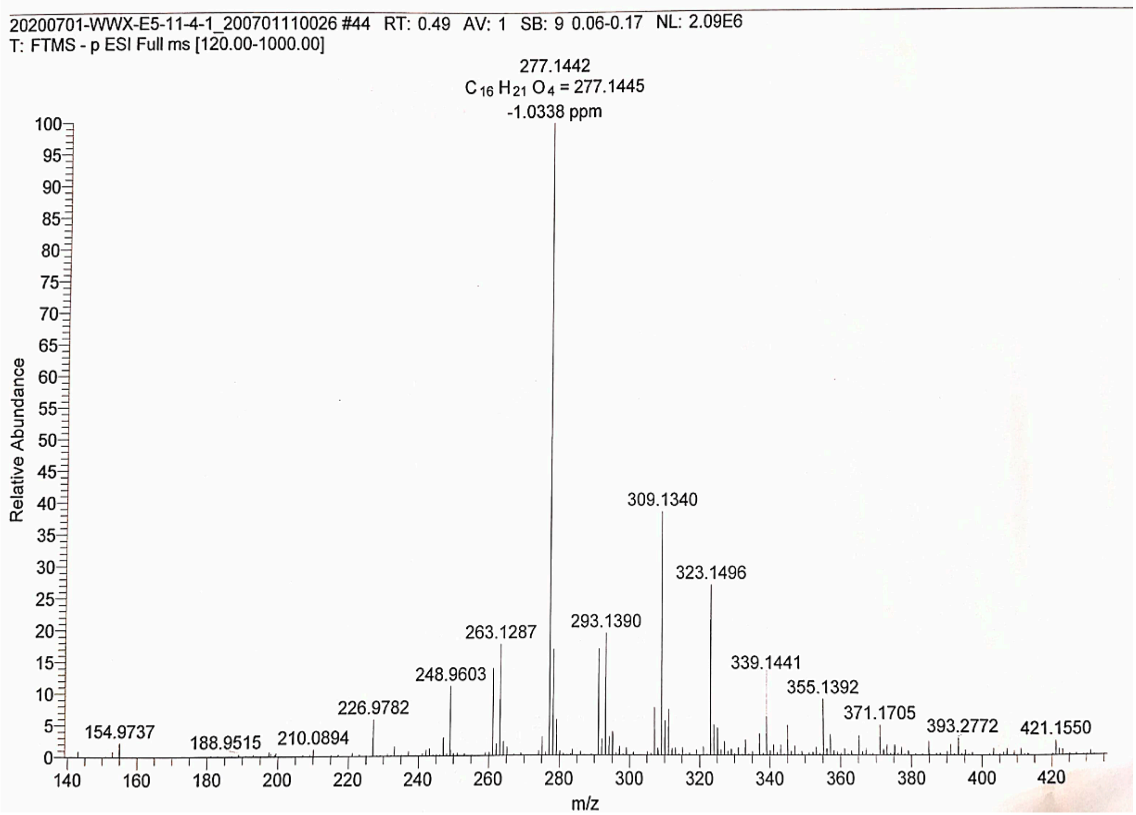


**Figure S28** IR spectrum (KBr) of brasilterpene D (**4**)

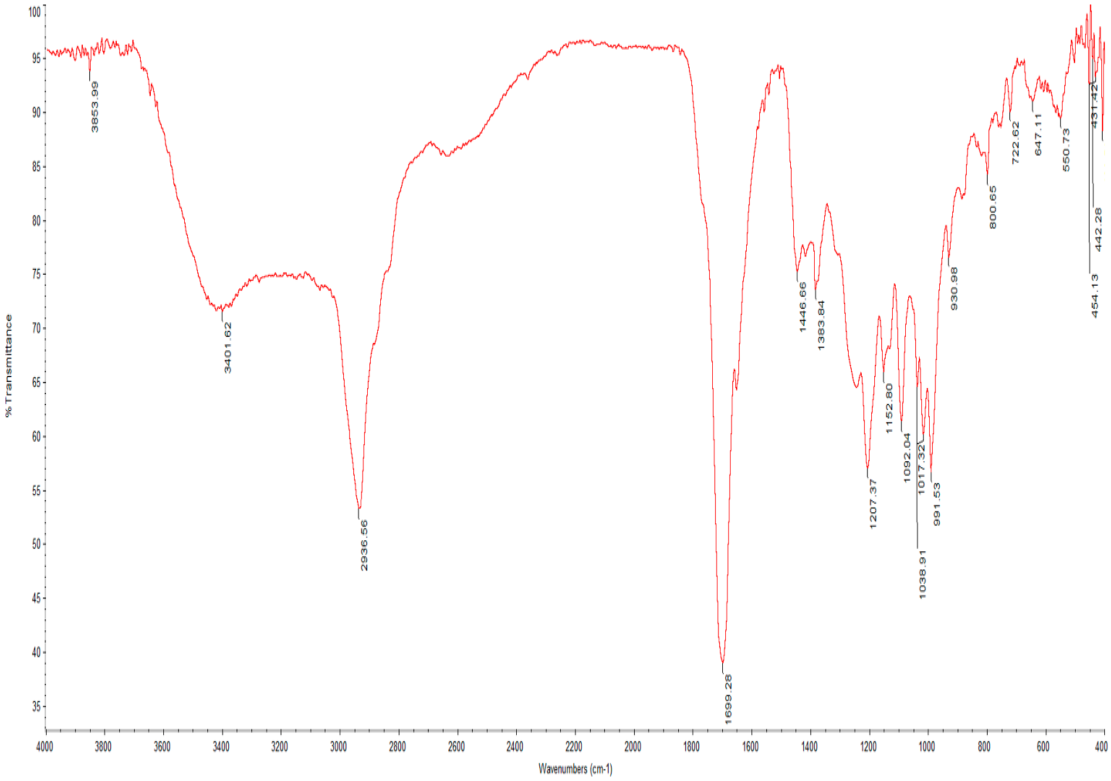


**Figure S29** $^{1}$H NMR spectrum (DMSO-$d_6$, 500 MHz) of brasilterpene D (4)

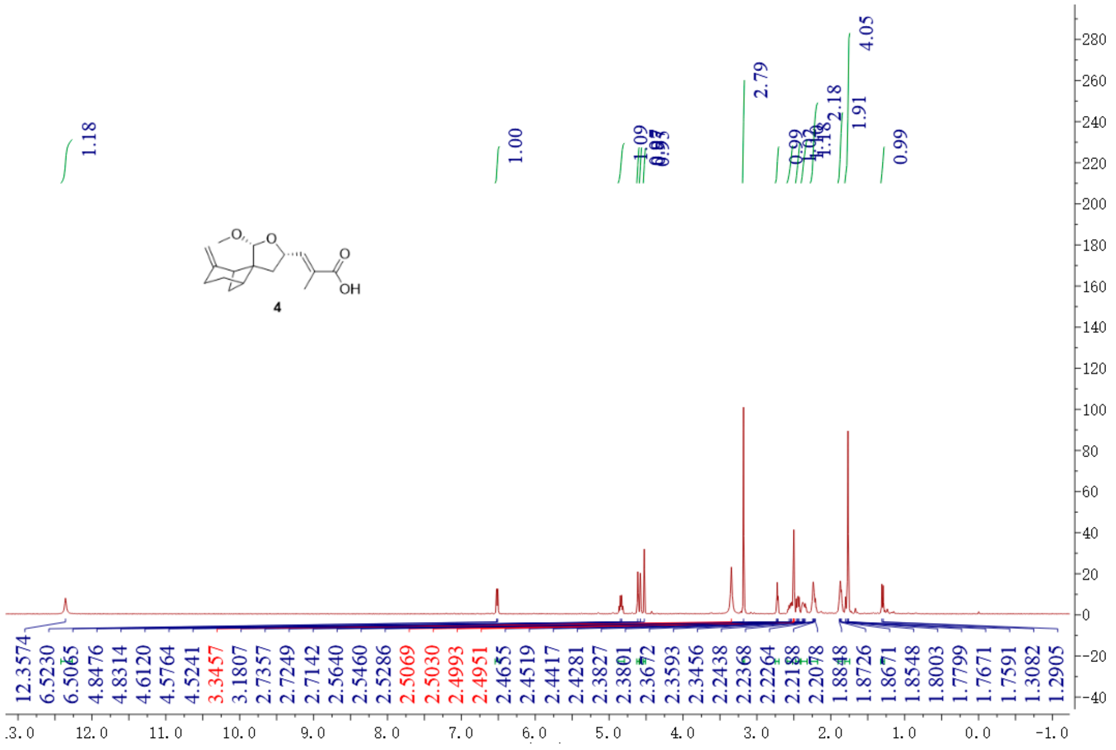

**Figure S30** $^{13}$C NMR spectrum (DMSO-$d_6$, 100 MHz) of brasilterpene D (**4**)

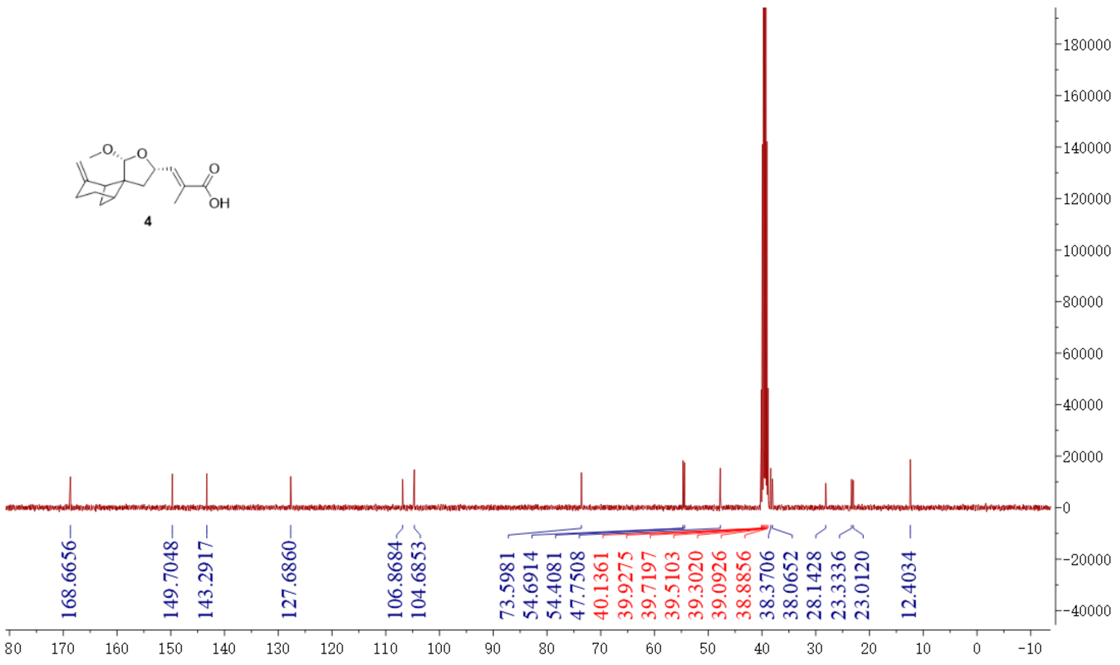

**Figure S31** HSQC spectrum of brasilterpene D (**4**)

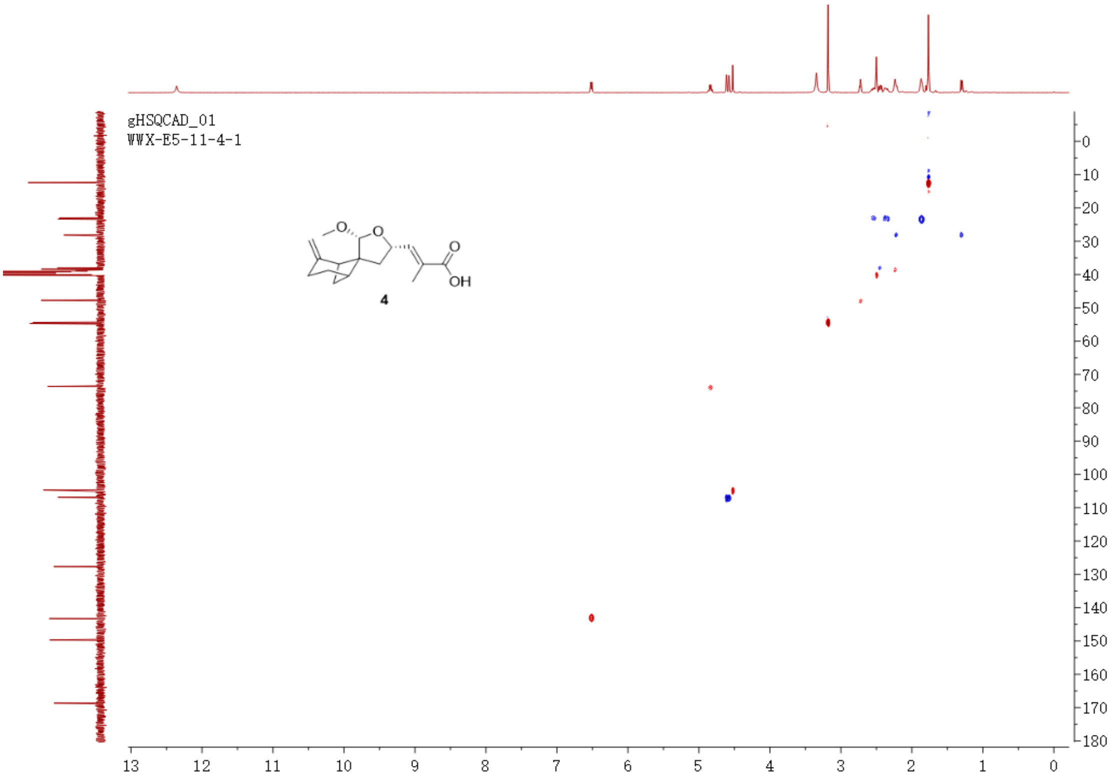


**Figure S32** HMBC spectrum of brasilterpene D (**4**)

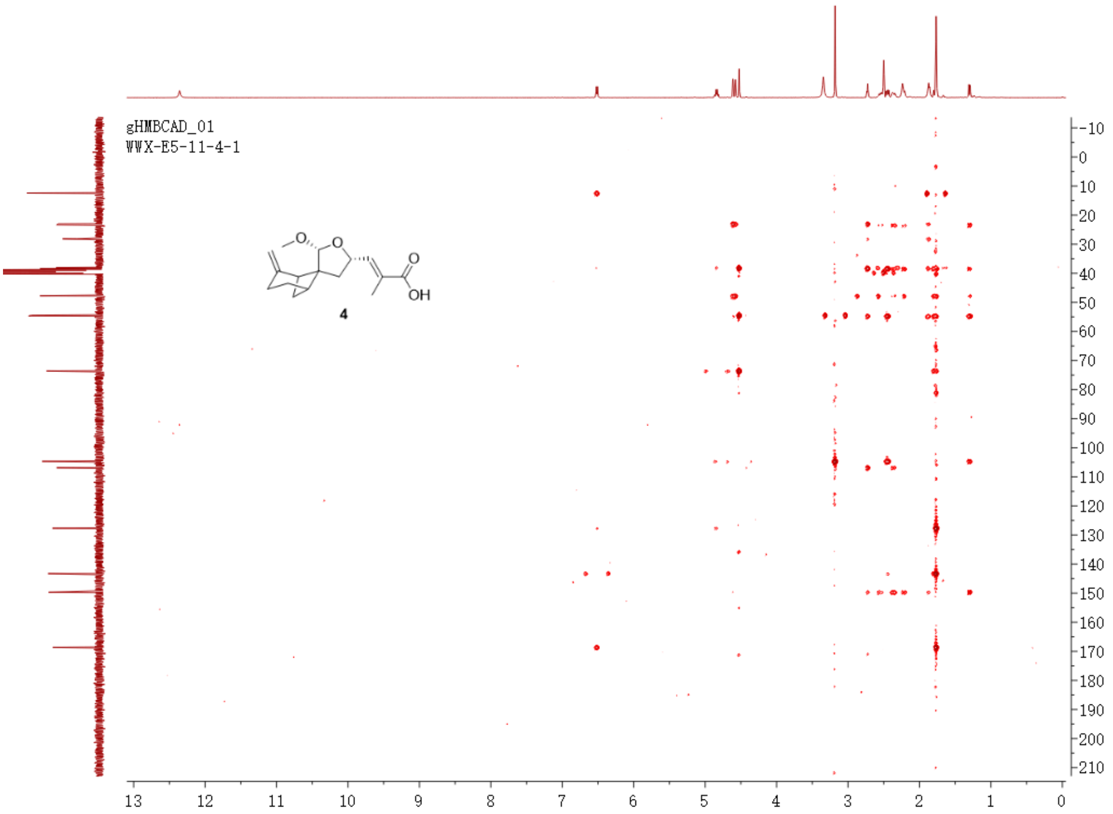


**Figure S33** $^{1}$H-$^{1}$H COSY spectrum of brasilterpene D (**4**)

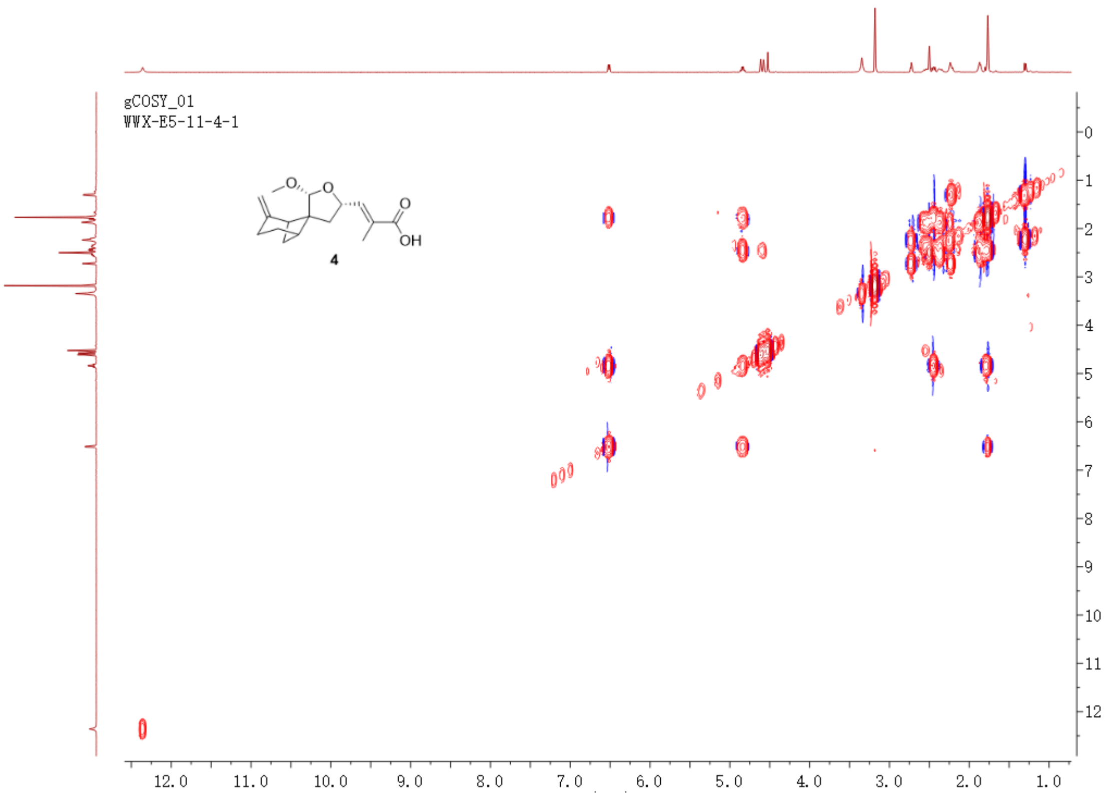


**Figure S34** NOESY spectrum of brasilterpene D (**4**)

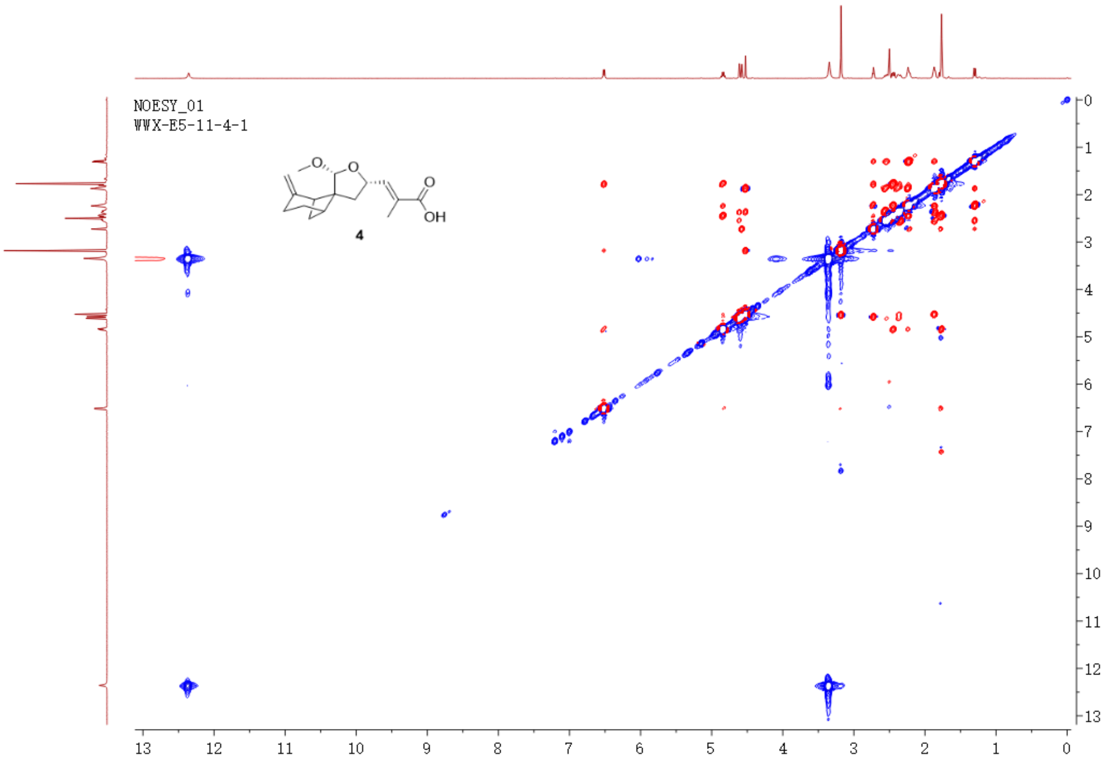


**Figure S35** HRESIMS spectrum of brasilterpene E (**5**)

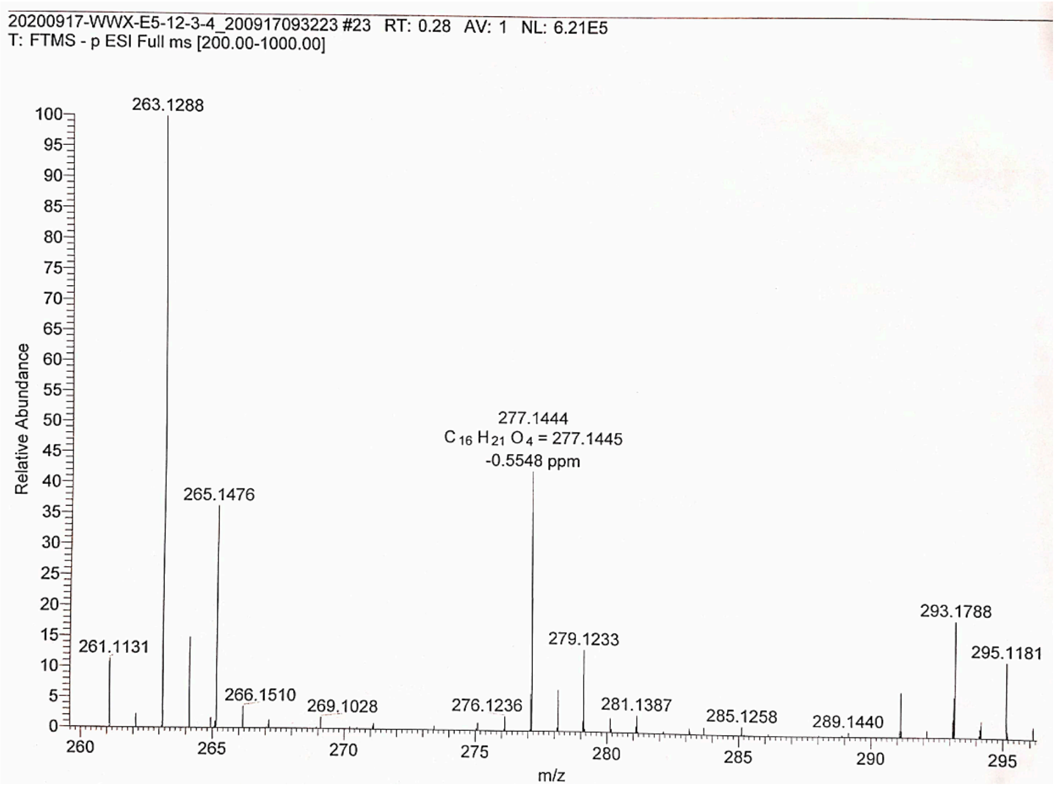


**Figure S36** IR spectrum (KBr) of brasilterpene E (**5**)

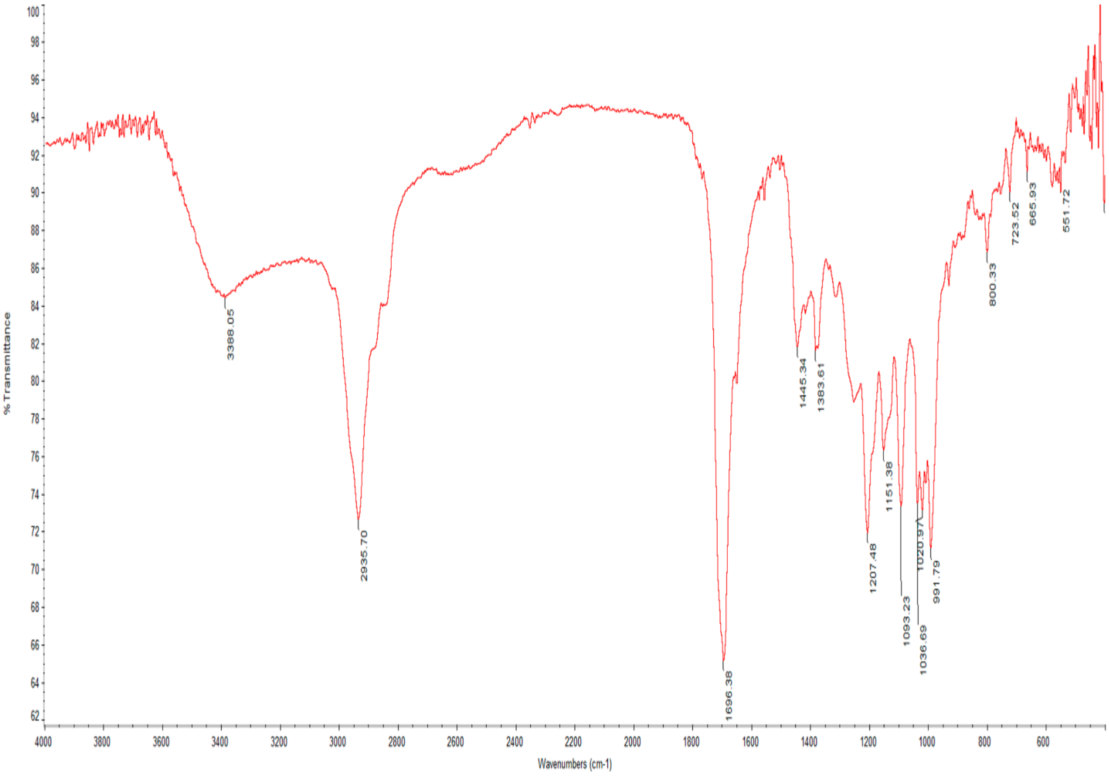

**Figure S37** $^{1}$H NMR spectrum (DMSO-$d_6$, 600 MHz) of brasilterpene E (**5**)

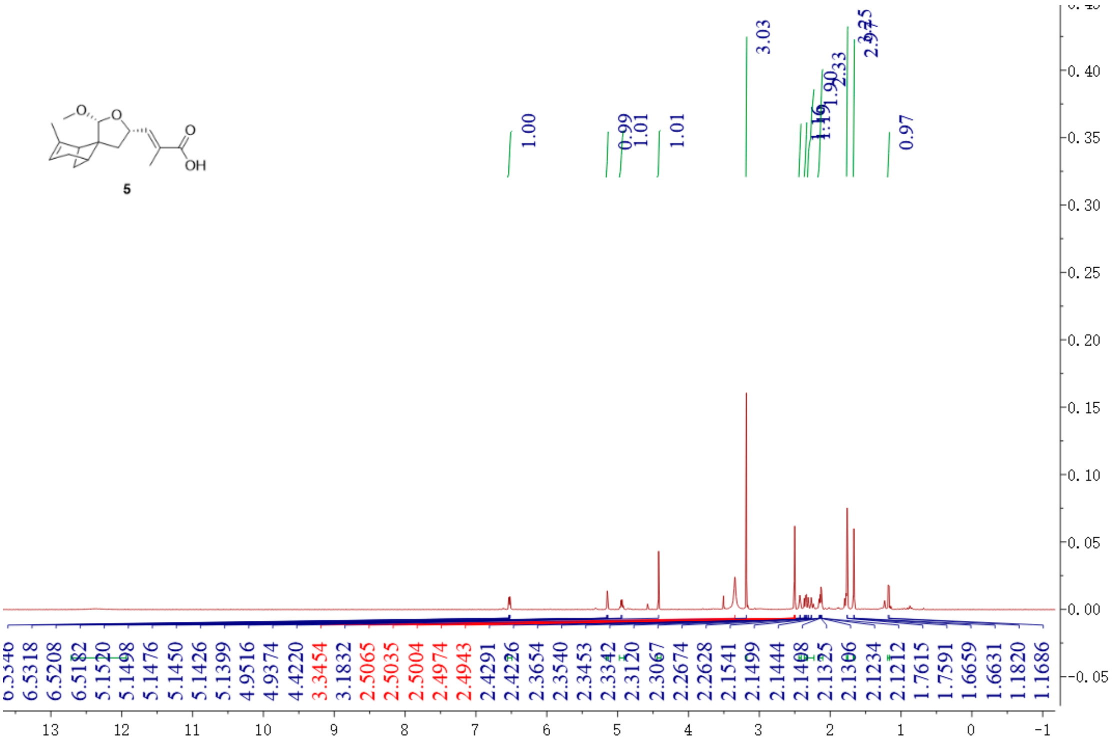

**Figure S38** DEPTQ-NMR spectrum (DMSO-$d_6$, 100 MHz) of brasilterpene E (**5**)

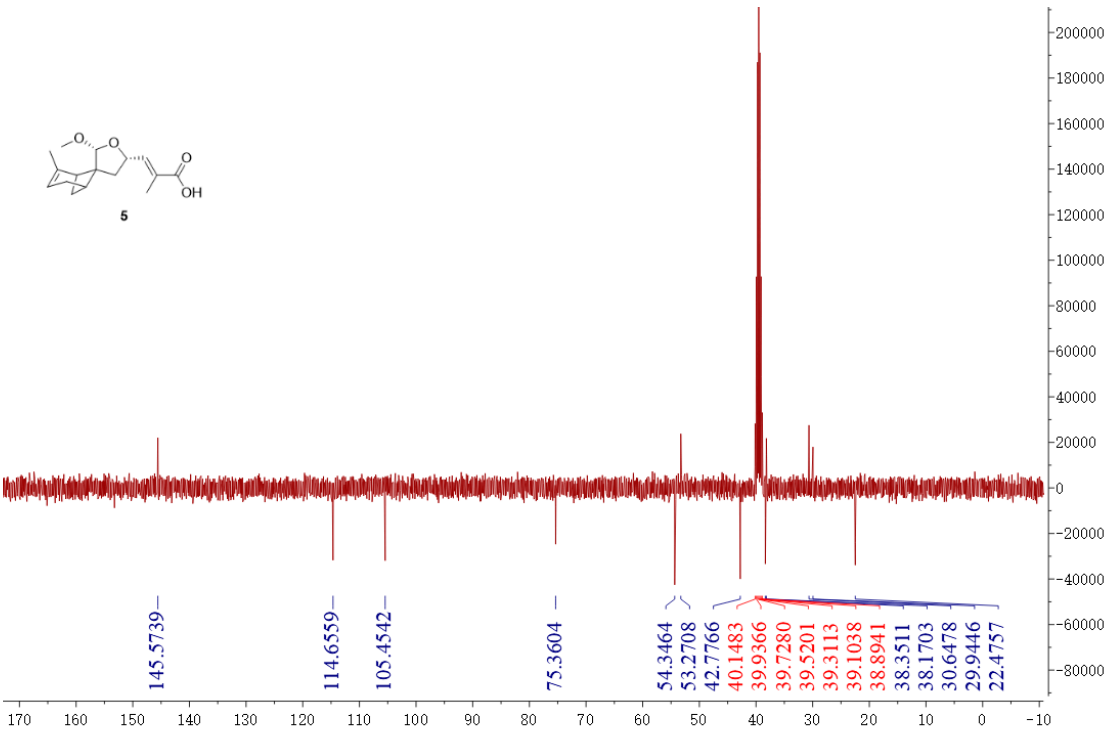

**Figure S39** HSQC spectrum of brasilterpene E (**5**)

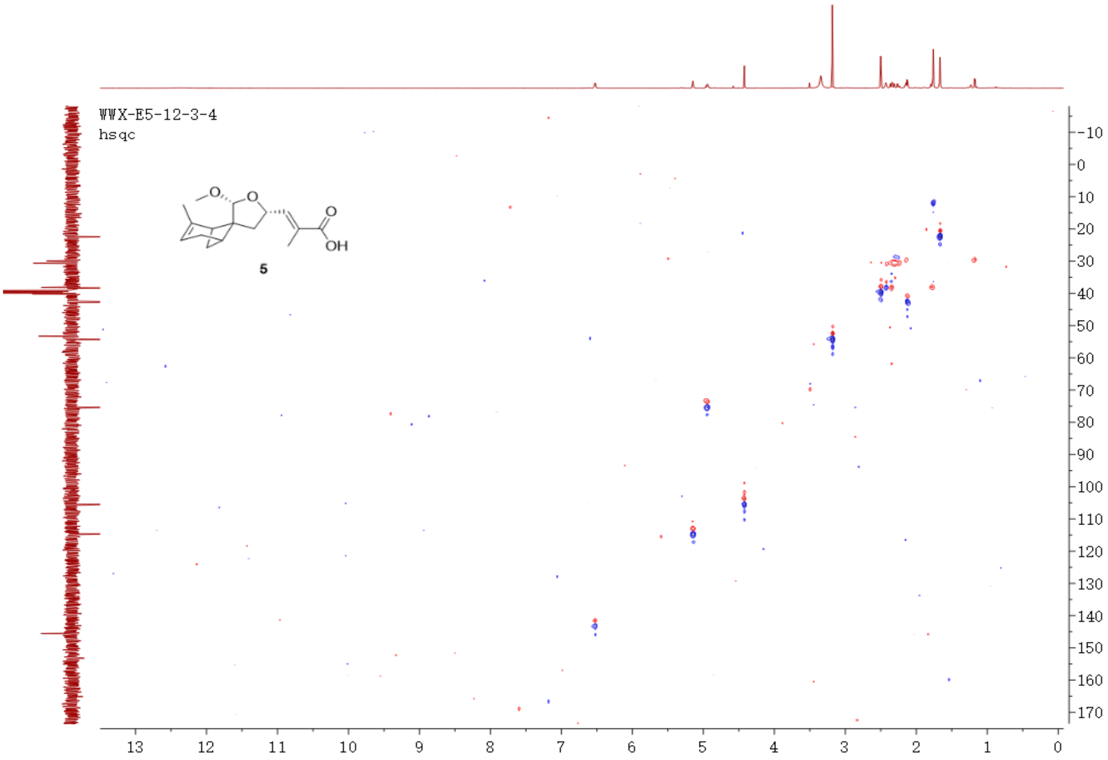


**Figure S40** HMBC spectrum of brasilterpene E (**5**)

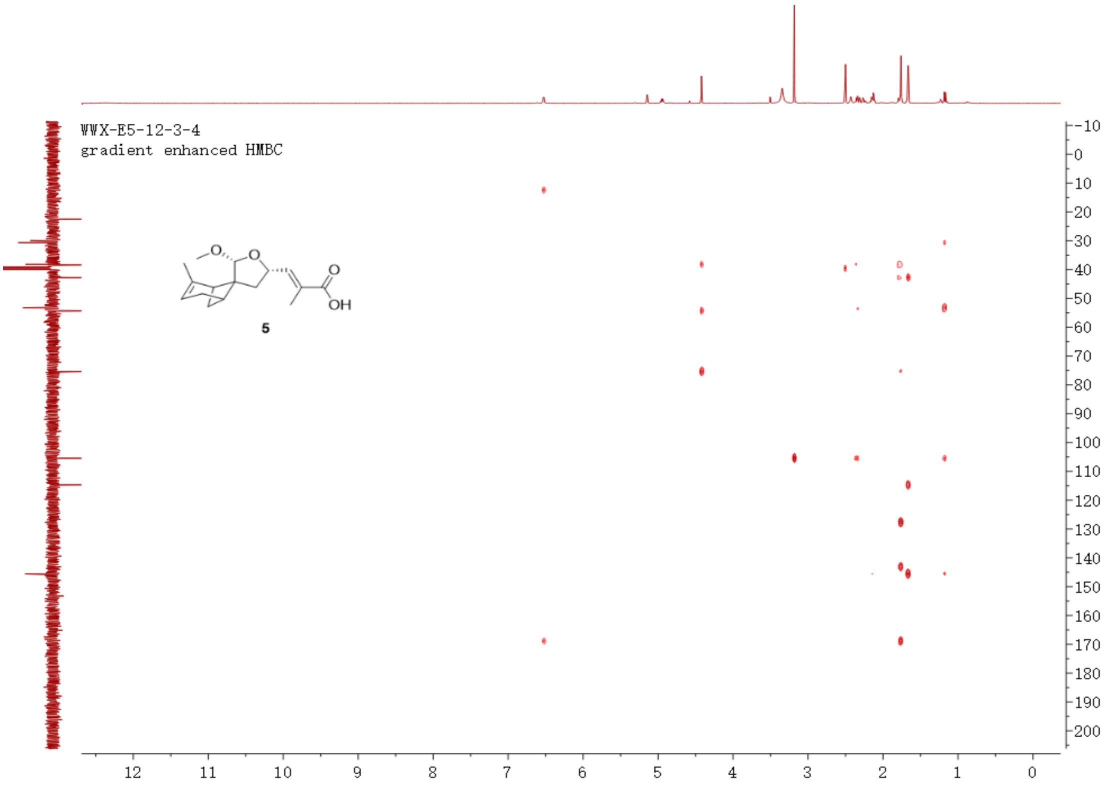

**Figure S41** $^{1}$H-$^{1}$H COSY spectrum of brasilterpene E (**5**)

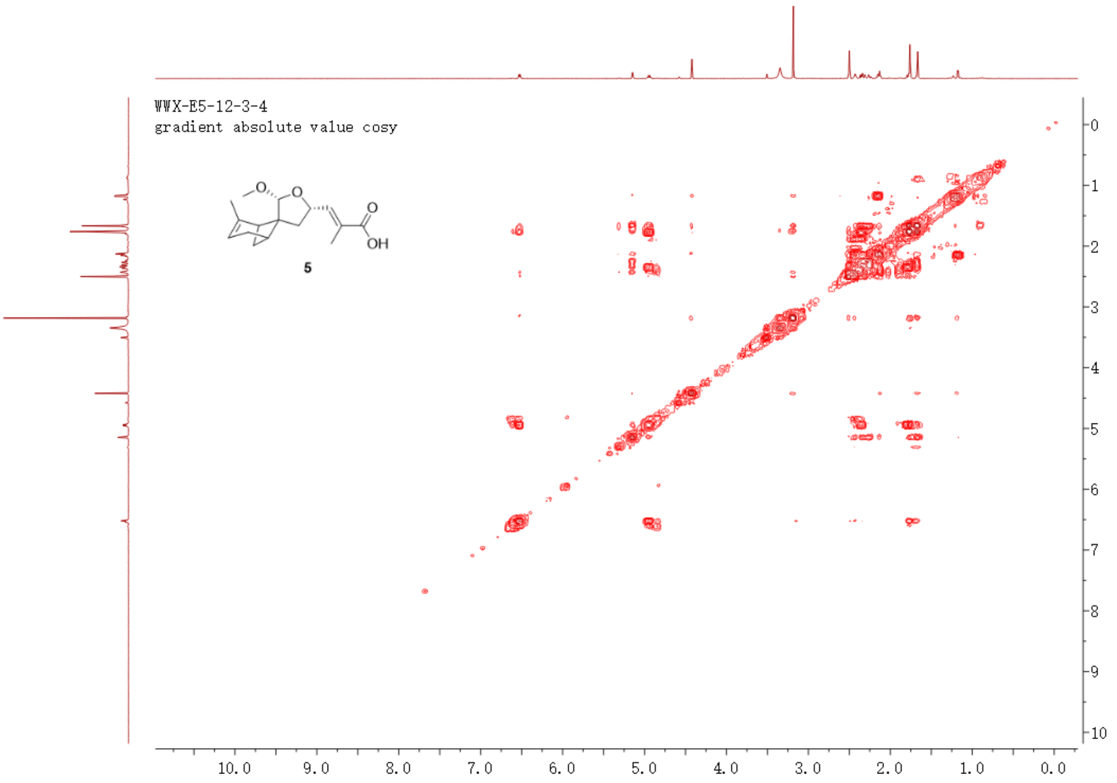


**Figure S42** NOESY spectrum of brasilterpene E (**5**)

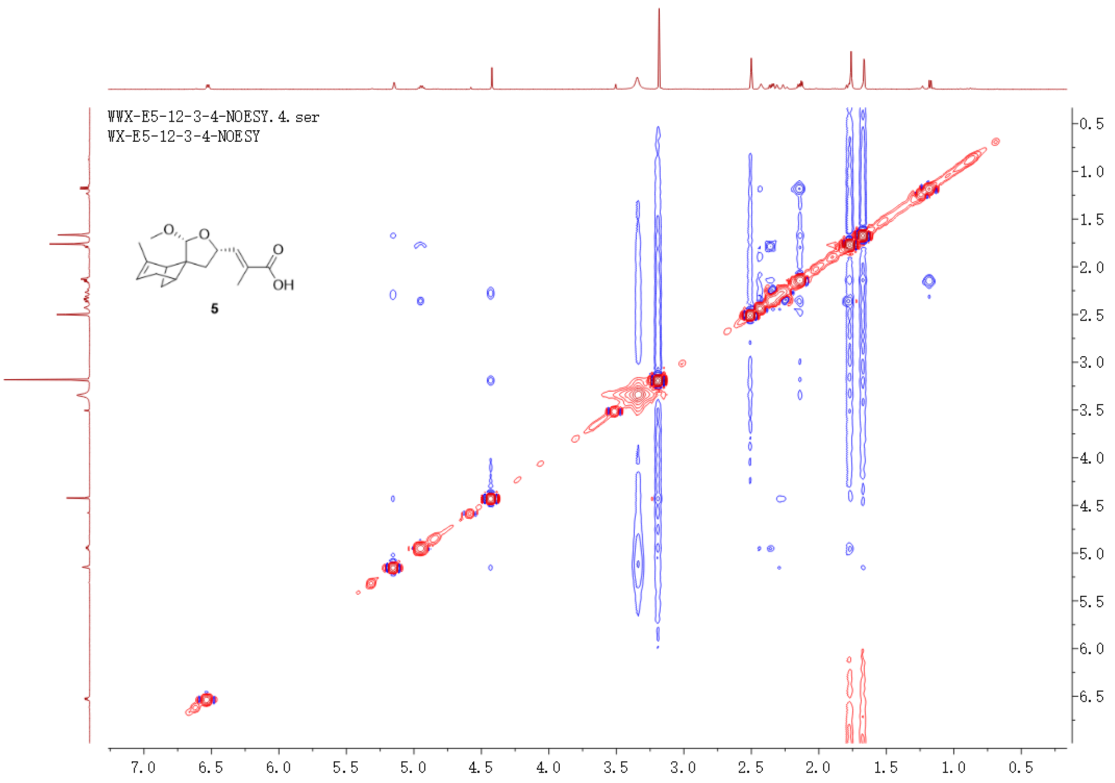

**Figure S43** DFT-optimized structures for low-energy conformers of **1a** (1*S*,3*R*,5*R*,6*S*,9*S*,14*S*-**1**) at B3LYP/6-31+G(d) level in DMSO (PCM).

Conformer populations were calculated using the Gibbs free energy and Boltzmann population at 298 K estimated thereof.

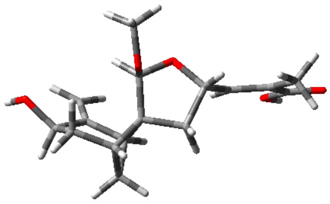

Conf. 1a-1 18.2%

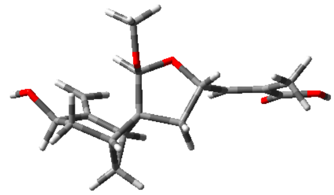

Conf. 1a-2 7.6%

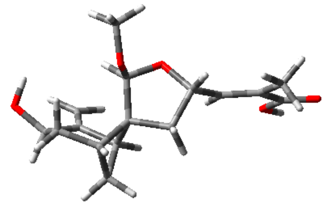

Conf. 1a-3 32.3%

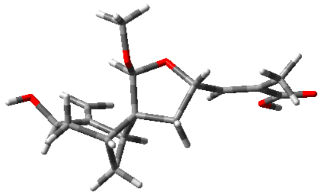

Conf. 1a-4 18.3%

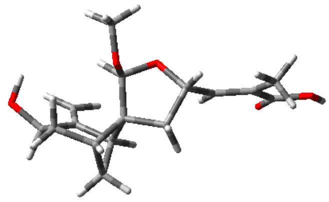

Conf. 1a-5 14.7%

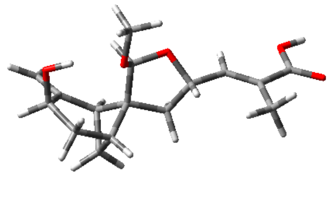

Conf. 1a-6 0.2%

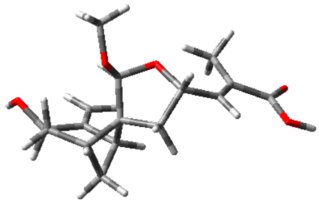

Conf. 1a-7 0.1%

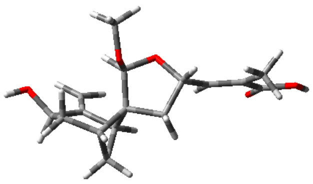

Conf. 1a-8 8.6%

**Table S1** Harmonic frequencies (cm**$^{-1}$) of compound **1a**.

| **Conformations** | **Frequencies** | | |
|---|---|---|---|
| | 1 | 2 | 3 |
| 1a-1 | 19.0735 | 20.4381 | 46.8963 |
| 1a-2 | 19.4948 | 25.5870 | 48.7451 |
| 1a-3 | 18.9596 | 20.4157 | 50.4323 |
| 1a-4 | 19.3234 | 21.1288 | 51.8031 |
| 1a-5 | 18.8909 | 25.8729 | 51.0282 |
| 1a-6 | 20.7770 | 26.2576 | 48.7462 |
| 1a-7 | 17.3998 | 22.5128 | 55.2759 |
| 1a-8 | 20.1087 | 25.8532 | 52.0789 |

**Table S2** Important thermodynamic parameters (a.u.) and Boltzmann distributions of the optimized compound **1a** at B3LYP/6-31G+(d) level of theory with PCM solvent model for DMSO.

| Conformations | E+ZPE | G | % |
|---|---|---|---|
| 1a-1 | -998.681195 | -998.732499 | 18.2% |
| 1a-2 | -998.680769 | -998.731668 | 7.6% |
| 1a-3 | -998.681877 | -998.733039 | 32.3% |
| 1a-4 | -998.681398 | -998.732500 | 18.3% |
| 1a-5 | -998.681464 | -998.732294 | 14.7% |
| 1a-6 | -998.677933 | -998.728364 | 0.2% |
| 1a-7 | -998.676975 | -998.727925 | 0.1% |
| 1a-8 | -998.681003 | -998.731790 | 8.6% |

E+ZPE, G: total energy with zero point energy (ZPE) and Gibbs free energy at B3LYP/6-31G+(d) level of theory with PCM solvent model for DMSO. %: Boltzmann distributions, using the relative Gibbs free energies as weighting factors.

**Table S3** Cartesian coordinates of the low-energy re-optimized conformers of compound **1a** (1*S*,3*R*,5*R*,6*S*,9*S*,14*S*-**1**) calculated at B3LYP/6-31+G(d) level of theory with PCM solvent model for DMSO.

| **Conformer 1a-1** | | Standard Orientation (Ångstroms) | | |
|---|---|---|---|---|
| I | Atom | X | Y | Z |

| | | | | |
|---|---|---|---|---|
| 1 | C | -1.18068 | -1.25636 | 1.99685 |
| 2 | C | -2.55339 | -0.64705 | 2.19793 |
| 3 | C | -2.58844 | 0.88969 | 2.04016 |
| 4 | C | -1.23468 | 1.45984 | 1.58704 |
| 5 | C | -0.50818 | 0.69 | 0.41353 |
| 6 | C | -0.09203 | -0.31358 | 1.5682 |
| 7 | C | -0.87564 | -2.54167 | 2.23415 |
| 8 | C | -0.08366 | 0.9451 | 2.49944 |
| 9 | C | 0.6513 | 1.41724 | -0.26873 |
| 10 | C | 0.88581 | 0.58166 | -1.52444 |
| 11 | O | -0.31821 | -0.15124 | -1.7888 |
| 12 | C | -1.29084 | 0.18441 | -0.79233 |
| 13 | O | -2.11544 | 1.25058 | -1.26498 |
| 14 | C | -3.07397 | 0.78761 | -2.20575 |
| 15 | C | 1.98861 | -0.42539 | -1.36078 |
| 16 | C | 3.26016 | -0.31453 | -1.79705 |
| 17 | C | 4.23381 | -1.41746 | -1.58571 |
| 18 | O | 3.76483 | -2.42782 | -0.83462 |
| 19 | C | 3.83604 | 0.87653 | -2.50538 |
| 20 | O | 5.3726 | -1.44172 | -2.01942 |
| 21 | O | -3.44418 | -1.20966 | 1.23664 |
| 22 | H | -2.94147 | -0.91116 | 3.18869 |
| 23 | H | -3.37232 | 1.1948 | 1.33685 |
| 24 | H | -2.84635 | 1.33999 | 3.00714 |
| 25 | H | -1.27234 | 2.54888 | 1.47586 |
| 26 | H | 0.88732 | -0.78892 | 1.45211 |
| 27 | H | 0.13096 | -2.92687 | 2.09608 |
| 28 | H | -1.62345 | -3.24758 | 2.58237 |
| 29 | H | 0.83525 | 1.54148 | 2.46575 |
| 30 | H | -0.32834 | 0.78456 | 3.55481 |
| 31 | H | 1.55081 | 1.53065 | 0.34423 |
| 32 | H | 0.33025 | 2.42466 | -0.56322 |
| 33 | H | 1.06893 | 1.22782 | -2.388 |
| 34 | H | -1.89124 | -0.7031 | -0.58739 |
| 35 | H | -3.66209 | 1.64462 | -2.54495 |
| 36 | H | -2.58257 | 0.3387 | -3.07415 |
| 37 | H | -3.7519 | 0.06603 | -1.73955 |
| 38 | H | 1.68199 | -1.33873 | -0.85302 |
| 39 | H | 4.51717 | -3.05377 | -0.80192 |
| 40 | H | 4.76717 | 1.19585 | -2.02415 |
| 41 | H | 4.05358 | 0.62861 | -3.54946 |

| | | | | |
|---|---|---|---|---|
| 42 | H | 3.16967 | 1.74282 | -2.49695 |
| 43 | H | -3.35488 | -2.1755 | 1.29293 |

| **Conformer 1a-2** | | Standard Orientation (Ångstroms) | | |
|---|---|---|---|---|
| I | Atom | X | Y | Z |
| 1 | C | -1.31627 | -1.58881 | 1.70311 |
| 2 | C | -2.63939 | -0.93698 | 2.0496 |
| 3 | C | -2.55465 | 0.59396 | 2.24198 |
| 4 | C | -1.1576 | 1.14591 | 1.91477 |
| 5 | C | -0.483 | 0.60556 | 0.59138 |
| 6 | C | -0.15485 | -0.6604 | 1.4879 |
| 7 | C | -1.11272 | -2.91395 | 1.64002 |
| 8 | C | -0.05723 | 0.35277 | 2.6775 |
| 9 | C | 0.735 | 1.37463 | 0.07794 |
| 10 | C | 0.91332 | 0.82493 | -1.33479 |
| 11 | O | -0.34503 | 0.27631 | -1.74833 |
| 12 | C | -1.29432 | 0.44706 | -0.68912 |
| 13 | O | -2.03382 | 1.65126 | -0.8976 |
| 14 | C | -3.02273 | 1.4851 | -1.9039 |
| 15 | C | 1.92281 | -0.28452 | -1.40865 |
| 16 | C | 3.1948 | -0.20138 | -1.84843 |
| 17 | C | 3.97861 | -1.46204 | -1.83552 |
| 18 | O | 5.15449 | -1.33623 | -2.47213 |
| 19 | C | 3.8826 | 1.04464 | -2.31611 |
| 20 | O | 3.65308 | -2.51905 | -1.32501 |
| 21 | O | -3.56405 | -1.19839 | 0.9955 |
| 22 | H | -3.05409 | -1.38686 | 2.95931 |
| 23 | H | -3.30758 | 1.10879 | 1.63351 |
| 24 | H | -2.78408 | 0.83349 | 3.28808 |
| 25 | H | -1.11049 | 2.23171 | 2.05111 |
| 26 | H | 0.78623 | -1.17138 | 1.2606 |
| 27 | H | -0.1374 | -3.3338 | 1.40792 |
| 28 | H | -1.91471 | -3.62157 | 1.82647 |
| 29 | H | 0.90511 | 0.86913 | 2.7697 |
| 30 | H | -0.32197 | -0.02122 | 3.67223 |
| 31 | H | 1.63586 | 1.27766 | 0.69173 |
| 32 | H | 0.4954 | 2.44412 | 0.01917 |
| 33 | H | 1.16248 | 1.6306 | -2.0315 |
| 34 | H | -1.96061 | -0.41681 | -0.68412 |
| 35 | H | -3.54231 | 2.4383 | -2.03372 |

| | | | | |
|---|---|---|---|---|
| 36 | H | -2.56512 | 1.20681 | -2.85798 |
| 37 | H | -3.75536 | 0.73061 | -1.60123 |
| 38 | H | 1.54485 | -1.25527 | -1.08603 |
| 39 | H | 5.55137 | -2.22858 | -2.39602 |
| 40 | H | 4.07301 | 0.99461 | -3.39312 |
| 41 | H | 3.30157 | 1.94986 | -2.12163 |
| 42 | H | 4.83916 | 1.17384 | -1.79793 |
| 43 | H | -3.54038 | -2.1544 | 0.82333 |

| **Conformer 1a-3** | | Standard Orientation (Ångstroms) | | |
|---|---|---|---|---|
| I | Atom | X | Y | Z |
| 1 | C | -1.00053 | 0.97991 | 2.23099 |
| 2 | C | -0.39119 | 2.28381 | 2.70961 |
| 3 | C | 0.93417 | 2.61054 | 1.9856 |
| 4 | C | 1.58852 | 1.34483 | 1.40402 |
| 5 | C | 0.70361 | 0.56235 | 0.3492 |
| 6 | C | -0.02451 | -0.00033 | 1.64066 |
| 7 | C | -2.30628 | 0.67247 | 2.24882 |
| 8 | C | 1.3815 | 0.10984 | 2.33015 |
| 9 | C | 1.39284 | -0.48971 | -0.51763 |
| 10 | C | 0.35203 | -0.76809 | -1.60141 |
| 11 | O | -0.54223 | 0.35504 | -1.6438 |
| 12 | C | -0.09941 | 1.32865 | -0.69642 |
| 13 | O | 0.77225 | 2.26913 | -1.32614 |
| 14 | C | 0.07503 | 3.10613 | -2.23882 |
| 15 | C | -0.4824 | -1.98344 | -1.3148 |
| 16 | C | -0.33315 | -3.21454 | -1.84547 |
| 17 | C | -1.2653 | -4.31466 | -1.48383 |
| 18 | O | -2.15234 | -3.99017 | -0.52852 |
| 19 | C | 0.74215 | -3.62232 | -2.80957 |
| 20 | O | -1.25525 | -5.43192 | -1.97058 |
| 21 | O | -1.30204 | 3.36009 | 2.5283 |
| 22 | H | -0.19412 | 2.19675 | 3.78475 |
| 23 | H | 0.78141 | 3.35546 | 1.19584 |
| 24 | H | 1.6184 | 3.07851 | 2.70449 |
| 25 | H | 2.62253 | 1.52468 | 1.09186 |
| 26 | H | -0.4075 | -1.02381 | 1.57812 |
| 27 | H | -2.68218 | -0.27364 | 1.87058 |
| 28 | H | -3.03749 | 1.36229 | 2.66239 |
| 29 | H | 2.0588 | -0.72997 | 2.13586 |

| | | | | |
|---|---|---|---|---|
| 30 | H | 1.39712 | 0.29228 | 3.40949 |
| 31 | H | 1.70969 | -1.39185 | 0.01477 |
| 32 | H | 2.28765 | -0.05542 | -0.9815 |
| 33 | H | 0.82913 | -0.83994 | -2.58325 |
| 34 | H | -0.96649 | 1.8526 | -0.2846 |
| 35 | H | 0.79017 | 3.81232 | -2.66932 |
| 36 | H | -0.36169 | 2.51849 | -3.05167 |
| 37 | H | -0.70409 | 3.67436 | -1.7215 |
| 38 | H | -1.30005 | -1.80524 | -0.61791 |
| 39 | H | -2.6768 | -4.80973 | -0.41701 |
| 40 | H | 0.31105 | -3.82467 | -3.79553 |
| 41 | H | 1.51828 | -2.86249 | -2.93126 |
| 42 | H | 1.2472 | -4.52955 | -2.45971 |
| 43 | H | -1.62848 | 3.31093 | 1.61474 |

| **Conformer 1a-4** | | Standard Orientation (Ångstroms) | | |
|---|---|---|---|---|
| I | Atom | X | Y | Z |
| 1 | C | -2.19149 | -0.12839 | 1.38068 |
| 2 | C | -2.419 | 1.06845 | 2.28135 |
| 3 | C | -1.12574 | 1.86597 | 2.56724 |
| 4 | C | 0.13454 | 1.0521 | 2.23315 |
| 5 | C | 0.20952 | 0.52305 | 0.74451 |
| 6 | C | -0.77076 | -0.6014 | 1.28055 |
| 7 | C | -3.13254 | -0.72893 | 0.63637 |
| 8 | C | -0.01995 | -0.43977 | 2.6465 |
| 9 | C | 1.5591 | 0.02121 | 0.2373 |
| 10 | C | 1.33715 | -0.0724 | -1.27129 |
| 11 | O | 0.23409 | 0.78409 | -1.60313 |
| 12 | C | -0.23028 | 1.4205 | -0.40868 |
| 13 | O | 0.41323 | 2.68859 | -0.25417 |
| 14 | C | -0.08039 | 3.63867 | -1.18893 |
| 15 | C | 0.97272 | -1.45692 | -1.72597 |
| 16 | C | 1.79247 | -2.36933 | -2.28714 |
| 17 | C | 1.27265 | -3.69408 | -2.71648 |
| 18 | O | -0.0097 | -3.91469 | -2.38286 |
| 19 | C | 3.26191 | -2.18582 | -2.52976 |
| 20 | O | 1.91789 | -4.544 | -3.30548 |
| 21 | O | -3.37615 | 1.93249 | 1.67946 |
| 22 | H | -2.83693 | 0.7083 | 3.22953 |
| 23 | H | -1.1168 | 2.81531 | 2.01885 |

| | | | | |
|---|---|---|---|---|
| 24 | H | -1.11034 | 2.13685 | 3.63046 |
| 25 | H | 1.04531 | 1.54105 | 2.59443 |
| 26 | H | -0.66317 | -1.59435 | 0.83252 |
| 27 | H | -2.90755 | -1.56324 | -0.02025 |
| 28 | H | -4.16179 | -0.38116 | 0.65972 |
| 29 | H | 0.92189 | -0.99538 | 2.7237 |
| 30 | H | -0.59208 | -0.64266 | 3.55767 |
| 31 | H | 1.89816 | -0.91987 | 0.68135 |
| 32 | H | 2.33162 | 0.77366 | 0.44098 |
| 33 | H | 2.21167 | 0.30107 | -1.81222 |
| 34 | H | -1.31337 | 1.55924 | -0.46961 |
| 35 | H | 0.44245 | 4.58447 | -1.02285 |
| 36 | H | 0.11215 | 3.31297 | -2.21545 |
| 37 | H | -1.15159 | 3.80485 | -1.03888 |
| 38 | H | -0.08024 | -1.70261 | -1.59457 |
| 39 | H | -0.18738 | -4.81012 | -2.73695 |
| 40 | H | 3.46379 | -2.1186 | -3.60383 |
| 41 | H | 3.66506 | -1.28777 | -2.0548 |
| 42 | H | 3.82645 | -3.03255 | -2.1237 |
| 43 | H | -3.54657 | 2.65114 | 2.3107 |

| **Conformer 1a-5** | | Standard Orientation (Ångstroms) | | |
|---|---|---|---|---|
| I | Atom | X | Y | Z |
| 1 | C | -2.26785 | -0.02308 | 1.42345 |
| 2 | C | -2.50518 | 1.18209 | 2.31312 |
| 3 | C | -1.22875 | 2.03269 | 2.49946 |
| 4 | C | 0.04427 | 1.21032 | 2.23198 |
| 5 | C | 0.14518 | 0.59358 | 0.77679 |
| 6 | C | -0.8428 | -0.49979 | 1.36288 |
| 7 | C | -3.19556 | -0.63986 | 0.67594 |
| 8 | C | -0.10297 | -0.25912 | 2.72627 |
| 9 | C | 1.50312 | 0.06337 | 0.32054 |
| 10 | C | 1.30643 | -0.11709 | -1.18412 |
| 11 | O | 0.20092 | 0.70916 | -1.58017 |
| 12 | C | -0.27363 | 1.41959 | -0.43483 |
| 13 | O | 0.36541 | 2.69442 | -0.34717 |
| 14 | C | -0.06603 | 3.57269 | -1.37778 |
| 15 | C | 0.95829 | -1.52678 | -1.56512 |
| 16 | C | 1.77947 | -2.45385 | -2.09868 |
| 17 | C | 1.17014 | -3.77256 | -2.40571 |

| | | | | |
|---|---|---|---|---|
| 18 | O | 2.00383 | -4.57108 | -3.09184 |
| 19 | C | 3.24031 | -2.27622 | -2.37965 |
| 20 | O | 0.05262 | -4.14864 | -2.10008 |
| 21 | O | -3.53991 | 2.0014 | 1.78462 |
| 22 | H | -2.83739 | 0.82447 | 3.29501 |
| 23 | H | -1.24769 | 2.92163 | 1.85806 |
| 24 | H | -1.20925 | 2.41188 | 3.52894 |
| 25 | H | 0.94613 | 1.72851 | 2.57418 |
| 26 | H | -0.73195 | -1.51677 | 0.97366 |
| 27 | H | -2.95607 | -1.48978 | 0.04311 |
| 28 | H | -4.23329 | -0.31697 | 0.6927 |
| 29 | H | 0.84194 | -0.8028 | 2.84177 |
| 30 | H | -0.67924 | -0.41745 | 3.64353 |
| 31 | H | 1.8347 | -0.8509 | 0.82243 |
| 32 | H | 2.27278 | 0.82635 | 0.4936 |
| 33 | H | 2.18677 | 0.23373 | -1.73048 |
| 34 | H | -1.35539 | 1.55716 | -0.51749 |
| 35 | H | 0.44258 | 4.53137 | -1.24469 |
| 36 | H | 0.19632 | 3.17663 | -2.36322 |
| 37 | H | -1.14531 | 3.74135 | -1.31261 |
| 38 | H | -0.08734 | -1.78965 | -1.40125 |
| 39 | H | 1.47729 | -5.38562 | -3.23007 |
| 40 | H | 3.42364 | -2.26946 | -3.45905 |
| 41 | H | 3.64318 | -1.34753 | -1.96734 |
| 42 | H | 3.82016 | -3.09075 | -1.93188 |
| 43 | H | -3.34989 | 2.13334 | 0.84119 |

| **Conformer 1a-6** | | Standard Orientation (Ångstroms) | | |
|---|---|---|---|---|
| I | Atom | X | Y | Z |
| 1 | C | -2.95208 | -0.28602 | 1.57667 |
| 2 | C | -2.72501 | 1.03133 | 2.28879 |
| 3 | C | -1.25562 | 1.28137 | 2.6946 |
| 4 | C | -0.31581 | 0.21328 | 2.1133 |
| 5 | C | -0.55789 | -0.30862 | 0.62373 |
| 6 | C | -1.73482 | -1.12141 | 1.30394 |
| 7 | C | -4.1565 | -0.72684 | 1.18349 |
| 8 | C | -0.8154 | -1.19599 | 2.56257 |
| 9 | C | 0.60105 | -1.15378 | 0.07396 |
| 10 | C | 1.18468 | -0.23375 | -0.98156 |
| 11 | O | 0.02307 | 0.31152 | -1.61145 |

| | | | | |
|---|---|---|---|---|
| 12 | C | -0.91496 | 0.60444 | -0.56319 |
| 13 | O | -0.82127 | 1.9742 | -0.17351 |
| 14 | C | -1.06811 | 2.85294 | -1.2693 |
| 15 | C | 1.9803 | -0.96269 | -2.01689 |
| 16 | C | 3.31764 | -0.93116 | -2.18978 |
| 17 | C | 3.95818 | -1.69345 | -3.29423 |
| 18 | O | 3.10914 | -2.46273 | -3.99496 |
| 19 | C | 4.29091 | -0.17284 | -1.33533 |
| 20 | O | 5.14533 | -1.666 | -3.56917 |
| 21 | O | -3.16946 | 2.07894 | 1.4305 |
| 22 | H | -3.35196 | 1.07457 | 3.18683 |
| 23 | H | -0.92045 | 2.28194 | 2.39914 |
| 24 | H | -1.17919 | 1.24964 | 3.78934 |
| 25 | H | 0.72906 | 0.42427 | 2.36658 |
| 26 | H | -1.95516 | -2.09295 | 0.84837 |
| 27 | H | -4.29169 | -1.67408 | 0.67195 |
| 28 | H | -5.04637 | -0.12834 | 1.36199 |
| 29 | H | -0.06219 | -1.99202 | 2.53356 |
| 30 | H | -1.31348 | -1.25859 | 3.53602 |
| 31 | H | 0.20474 | -2.07073 | -0.38101 |
| 32 | H | 1.35086 | -1.43565 | 0.81977 |
| 33 | H | 1.73441 | 0.59823 | -0.52923 |
| 34 | H | -1.91894 | 0.3972 | -0.94963 |
| 35 | H | -1.08145 | 3.87662 | -0.8853 |
| 36 | H | -0.27149 | 2.77746 | -2.015 |
| 37 | H | -2.03978 | 2.63975 | -1.72524 |
| 38 | H | 1.37116 | -1.5563 | -2.69812 |
| 39 | H | 3.68209 | -2.88206 | -4.66921 |
| 40 | H | 4.72754 | 0.65606 | -1.90213 |
| 41 | H | 3.83736 | 0.24068 | -0.43133 |
| 42 | H | 5.10227 | -0.8299 | -1.00281 |
| 43 | H | -2.4307 | 2.27145 | 0.8233 |

| **Conformer 1a-7** | | Standard Orientation (Ångstroms) | | |
|---|---|---|---|---|
| I | Atom | X | Y | Z |
| 1 | C | -1.71226 | -0.07967 | 1.38357 |
| 2 | C | -2.2423 | 1.33115 | 1.53836 |
| 3 | C | -1.15172 | 2.38298 | 1.84269 |
| 4 | C | 0.26406 | 1.78531 | 1.79526 |
| 5 | C | 0.58106 | 0.83277 | 0.57504 |

| | | | | |
|---|---|---|---|---|
| 6 | C | -0.21885 | -0.23614 | 1.43113 |
| 7 | C | -2.48033 | -1.17442 | 1.26929 |
| 8 | C | 0.35454 | 0.50985 | 2.68227 |
| 9 | C | 2.05092 | 0.49983 | 0.31921 |
| 10 | C | 1.99664 | -0.17941 | -1.04945 |
| 11 | O | 0.85604 | 0.34506 | -1.72799 |
| 12 | C | 0.1163 | 1.18514 | -0.8329 |
| 13 | O | 0.4474 | 2.55222 | -1.07988 |
| 14 | C | -0.1959 | 3.03968 | -2.24878 |
| 15 | C | 1.94379 | -1.68475 | -0.93222 |
| 16 | C | 1.05848 | -2.57368 | -1.43398 |
| 17 | C | 1.20181 | -4.02284 | -1.12849 |
| 18 | O | 2.34038 | -4.33722 | -0.48628 |
| 19 | C | -0.10954 | -2.22992 | -2.31187 |
| 20 | O | 0.39504 | -4.89194 | -1.40934 |
| 21 | O | -2.89053 | 1.70541 | 0.32423 |
| 22 | H | -2.99921 | 1.36527 | 2.33076 |
| 23 | H | -1.21918 | 3.23048 | 1.15038 |
| 24 | H | -1.3211 | 2.78912 | 2.84804 |
| 25 | H | 1.029 | 2.5401 | 2.00704 |
| 26 | H | 0.12161 | -1.27216 | 1.33725 |
| 27 | H | -2.05189 | -2.16922 | 1.18074 |
| 28 | H | -3.56414 | -1.11028 | 1.2681 |
| 29 | H | 1.37077 | 0.21823 | 2.97102 |
| 30 | H | -0.25692 | 0.49931 | 3.59082 |
| 31 | H | 2.52662 | -0.11362 | 1.09022 |
| 32 | H | 2.62815 | 1.42995 | 0.23871 |
| 33 | H | 2.89112 | 0.07551 | -1.62909 |
| 34 | H | -0.94749 | 1.0107 | -1.00163 |
| 35 | H | 0.10724 | 4.07953 | -2.39788 |
| 36 | H | 0.10326 | 2.46168 | -3.12829 |
| 37 | H | -1.28327 | 3.01052 | -2.12897 |
| 38 | H | 2.77091 | -2.06937 | -0.33453 |
| 39 | H | 2.27676 | -5.30863 | -0.37858 |
| 40 | H | -0.91254 | -1.77752 | -1.72169 |
| 41 | H | 0.18412 | -1.54633 | -3.11448 |
| 42 | H | -0.52592 | -3.10957 | -2.81355 |
| 43 | H | -3.53293 | 1.0069 | 0.11574 |

| **Conformer 1a-8** | Standard Orientation (Ångstroms) |
|---|---|

| I | Atom | X | Y | Z |
|---|---|---|---|---|
| 1 | C | -2.23393 | -0.45057 | 1.33805 |
| 2 | C | -3.16276 | 0.74415 | 1.40681 |
| 3 | C | -2.44569 | 2.08646 | 1.1341 |
| 4 | C | -0.91957 | 1.95712 | 1.26219 |
| 5 | C | -0.26436 | 0.85617 | 0.33459 |
| 6 | C | -0.7706 | -0.14044 | 1.45858 |
| 7 | C | -2.61514 | -1.71738 | 1.11418 |
| 8 | C | -0.52021 | 1.04233 | 2.45537 |
| 9 | C | 1.24909 | 0.90301 | 0.13984 |
| 10 | C | 1.46592 | -0.04263 | -1.04033 |
| 11 | O | 0.2216 | -0.15046 | -1.74687 |
| 12 | C | -0.74775 | 0.68155 | -1.10227 |
| 13 | O | -0.76792 | 1.97013 | -1.72326 |
| 14 | C | -1.37951 | 1.91858 | -3.00522 |
| 15 | C | 1.85314 | -1.43 | -0.61633 |
| 16 | C | 3.08583 | -1.97608 | -0.64186 |
| 17 | C | 3.19074 | -3.38473 | -0.18521 |
| 18 | O | 4.40152 | -3.91076 | -0.43287 |
| 19 | C | 4.3456 | -1.28194 | -1.06078 |
| 20 | O | 2.3218 | -4.04333 | 0.35765 |
| 21 | O | -4.2124 | 0.57659 | 0.46053 |
| 22 | H | -3.61056 | 0.77204 | 2.40788 |
| 23 | H | -2.70796 | 2.48159 | 0.14559 |
| 24 | H | -2.80766 | 2.82887 | 1.8565 |
| 25 | H | -0.42294 | 2.93282 | 1.24016 |
| 26 | H | -0.14418 | -1.01768 | 1.6489 |
| 27 | H | -1.90266 | -2.53316 | 1.04091 |
| 28 | H | -3.66511 | -1.96501 | 0.98416 |
| 29 | H | 0.51782 | 1.151 | 2.79065 |
| 30 | H | -1.15548 | 1.0875 | 3.34595 |
| 31 | H | 1.83655 | 0.63102 | 1.0223 |
| 32 | H | 1.55118 | 1.91695 | -0.15192 |
| 33 | H | 2.1992 | 0.37579 | -1.73589 |
| 34 | H | -1.73429 | 0.21617 | -1.1809 |
| 35 | H | -1.36846 | 2.92621 | -3.42953 |
| 36 | H | -0.82551 | 1.25506 | -3.67585 |
| 37 | H | -2.42005 | 1.59006 | -2.92178 |
| 38 | H | 1.01712 | -2.04412 | -0.27941 |
| 39 | H | 4.32326 | -4.82753 | -0.09653 |
| 40 | H | 4.71712 | -1.69978 | -2.00211 |

| | | | | |
|---|---|---|---|---|
| 41 | H | 4.21182 | -0.20643 | -1.20277 |
| 42 | H | 5.12175 | -1.3986 | -0.29644 |
| 43 | H | -4.82634 | 1.31946 | 0.58324 |

**Figure S44** DFT-optimized structures for low-energy conformers of **1b** (1*S*,3*S*,5*R*,6*S*,9*S*,14*S*-**1**) at B3LYP/6-31+G(d) level in DMSO (PCM).

Conformer populations were calculated using the Gibbs free energy and Boltzmann population at 298 K estimated thereof.

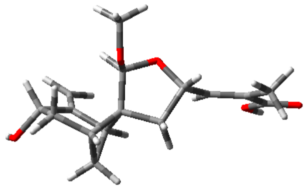

Conf. 1b-1 55.7%

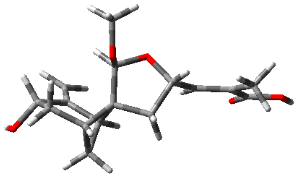

Conf. 1b-2 31.6%

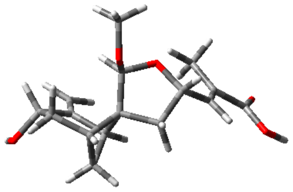

Conf. 1b-3 1.55%

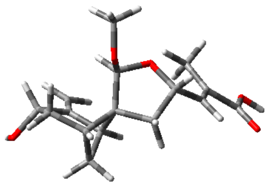

Conf. 1b-4 0.7%

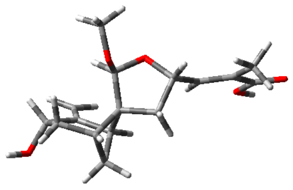

Conf. 1b-5 10.4%

**Table S4** Harmonic frequencies (cm**$^{-1}$) of compound **1b**.

| **Conformations** | **Frequencies** | | |
|---|---|---|---|
| | 1 | 2 | 3 |
| 1b-1 | 21.7274 | 26.1484 | 51.5239 |
| 1b-2 | 22.0726 | 26.3038 | 51.6332 |
| 1b-3 | 11.3854 | 25.0126 | 56.9492 |

| | | | |
|---|---|---|---|
| 1b-4 | 12.5742 | 26.1711 | 57.0078 |
| 1b-5 | 20.2057 | 25.3751 | 51.0603 |

**Table S5** Important thermodynamic parameters (a.u.) and Boltzmann distributions of the optimized compound **1b** at B3LYP/6-31G+(d) level of theory with PCM solvent model for DMSO.

| Conformations | E+ZPE | G | % |
|---|---|---|---|
| 1b-1 | -998.681927 | -998.732670 | 55.7% |
| 1b-2 | -998.681532 | -998.732136 | 31.6% |
| 1b-3 | -998.677949 | -998.729289 | 1.5% |
| 1b-4 | -998.677605 | -998.728595 | 0.7% |
| 1b-5 | -998.680205 | -998.731088 | 10.4% |

E+ZPE, G: total energy with zero point energy (ZPE) and Gibbs free energy at B3LYP/6-31G+(d) level of theory with PCM solvent model for DMSO. %: Boltzmann distributions, using the relative Gibbs free energies as weighting factors

**Table S6** Cartesian coordinates of the low-energy re-optimized conformers of compound **1b** (1*S*,3*S*,5*R*,6*S*,9*S*,14*S*-**1**) calculated at B3LYP/6-31+G(d) level of theory with PCM solvent model for DMSO.

| **Conformer 1b-1** | | Standard Orientation (Ångstroms) | | |
|---|---|---|---|---|
| I | Atom | X | Y | Z |
| 1 | C | 2.54326 | -0.28775 | -0.45061 |
| 2 | C | 3.47355 | 0.39018 | 0.5294 |
| 3 | C | 2.74563 | 1.42362 | 1.41713 |
| 4 | C | 1.41948 | 1.88673 | 0.77813 |
| 5 | C | 0.37884 | 0.72555 | 0.49251 |
| 6 | C | 1.28748 | 0.46869 | -0.78289 |
| 7 | C | 2.72638 | -1.51813 | -0.95159 |
| 8 | C | 1.52448 | 2.0202 | -0.77012 |
| 9 | C | -1.06647 | 1.11706 | 0.19013 |
| 10 | C | -1.80834 | -0.21403 | 0.30836 |
| 11 | O | -0.98943 | -1.10142 | 1.08646 |
| 12 | C | 0.17552 | -0.39095 | 1.51157 |
| 13 | O | -0.05196 | 0.20649 | 2.78973 |
| 14 | C | -0.13403 | -0.76891 | 3.81987 |
| 15 | C | -2.0365 | -0.87835 | -1.01915 |
| 16 | C | -3.17689 | -0.8834 | -1.73951 |
| 17 | C | -3.25631 | -1.61807 | -3.0293 |
| 18 | O | -2.09009 | -2.15101 | -3.43042 |

| | | | | |
|---|---|---|---|---|
| 19 | C | -4.44626 | -0.1762 | -1.3645 |
| 20 | O | -4.26212 | -1.74137 | -3.70648 |
| 21 | O | 4.48256 | 1.06743 | -0.21679 |
| 22 | H | 3.98435 | -0.34038 | 1.16644 |
| 23 | H | 3.38812 | 2.29541 | 1.58981 |
| 24 | H | 2.53364 | 0.9836 | 2.39812 |
| 25 | H | 1.00239 | 2.75855 | 1.29305 |
| 26 | H | 0.77729 | 0.10625 | -1.68086 |
| 27 | H | 2.00641 | -1.97572 | -1.62471 |
| 28 | H | 3.60408 | -2.10577 | -0.69836 |
| 29 | H | 0.72753 | 2.61271 | -1.23495 |
| 30 | H | 2.47026 | 2.39039 | -1.17627 |
| 31 | H | -1.22028 | 1.60888 | -0.77533 |
| 32 | H | -1.43185 | 1.80279 | 0.96518 |
| 33 | H | -2.7493 | -0.0824 | 0.85063 |
| 34 | H | 1.01919 | -1.08403 | 1.5765 |
| 35 | H | -0.97919 | -1.44308 | 3.65258 |
| 36 | H | 0.79776 | -1.339 | 3.88522 |
| 37 | H | -0.28943 | -0.25021 | 4.76975 |
| 38 | H | -1.17242 | -1.42388 | -1.39535 |
| 39 | H | -2.31975 | -2.58808 | -4.27618 |
| 40 | H | -4.805 | 0.43656 | -2.19899 |
| 41 | H | -5.22445 | -0.90301 | -1.10939 |
| 42 | H | -4.32464 | 0.49906 | -0.51375 |
| 43 | H | 4.76649 | 0.46501 | -0.92504 |

| **Conformer 1b-2** | | Standard Orientation (Ångstroms) | | |
|---|---|---|---|---|
| I | Atom | X | Y | Z |
| 1 | C | 2.48443 | 0.72767 | -0.48921 |
| 2 | C | 3.24833 | 1.3309 | 0.66733 |
| 3 | C | 2.32509 | 1.74018 | 1.83619 |
| 4 | C | 0.86463 | 1.91889 | 1.37037 |
| 5 | C | 0.21513 | 0.63144 | 0.71149 |
| 6 | C | 1.02956 | 1.0963 | -0.56888 |
| 7 | C | 2.99175 | -0.1535 | -1.36357 |
| 8 | C | 0.77107 | 2.55425 | -0.04841 |
| 9 | C | -1.30223 | 0.60214 | 0.53781 |
| 10 | C | -1.575 | -0.86497 | 0.20797 |
| 11 | O | -0.4543 | -1.63176 | 0.67517 |
| 12 | C | 0.4706 | -0.74742 | 1.31207 |

| | | | | |
|---|---|---|---|---|
| 13 | O | 0.19922 | -0.68134 | 2.7138 |
| 14 | C | 0.53209 | -1.89591 | 3.37208 |
| 15 | C | -1.69695 | -1.12255 | -1.26606 |
| 16 | C | -2.83247 | -1.29542 | -1.97253 |
| 17 | C | -2.66927 | -1.56202 | -3.42402 |
| 18 | O | -3.83085 | -1.88421 | -4.01609 |
| 19 | C | -4.22593 | -1.21655 | -1.42793 |
| 20 | O | -1.63362 | -1.51188 | -4.06337 |
| 21 | O | 3.91938 | 2.49659 | 0.1933 |
| 22 | H | 4.02037 | 0.64664 | 1.03635 |
| 23 | H | 2.67742 | 2.6747 | 2.28916 |
| 24 | H | 2.35818 | 0.97369 | 2.61865 |
| 25 | H | 0.24899 | 2.40044 | 2.13735 |
| 26 | H | 0.5729 | 0.88694 | -1.54128 |
| 27 | H | 2.38797 | -0.58584 | -2.15731 |
| 28 | H | 4.02921 | -0.47028 | -1.30878 |
| 29 | H | -0.21231 | 2.96744 | -0.30242 |
| 30 | H | 1.5099 | 3.3199 | -0.30221 |
| 31 | H | -1.69523 | 1.29078 | -0.21649 |
| 32 | H | -1.78627 | 0.85309 | 1.49026 |
| 33 | H | -2.45909 | -1.22076 | 0.74508 |
| 34 | H | 1.48935 | -1.11245 | 1.15297 |
| 35 | H | -0.07147 | -2.72477 | 2.99064 |
| 36 | H | 1.59721 | -2.11789 | 3.25474 |
| 37 | H | 0.31922 | -1.7762 | 4.43784 |
| 38 | H | -0.74227 | -1.19479 | -1.78806 |
| 39 | H | -3.57604 | -2.03821 | -4.9494 |
| 40 | H | -4.7007 | -2.20291 | -1.44904 |
| 41 | H | -4.26039 | -0.85228 | -0.39792 |
| 42 | H | -4.83255 | -0.524 | -2.0217 |
| 43 | H | 4.29493 | 2.27593 | -0.67596 |

| **Conformer 1b-3** | | Standard Orientation (Ångstroms) | | |
|---|---|---|---|---|
| I | Atom | X | Y | Z |
| 1 | C | 1.62992 | -0.0037 | -1.43887 |
| 2 | C | 3.01196 | 0.32763 | -0.92357 |
| 3 | C | 2.97556 | 1.17759 | 0.36561 |
| 4 | C | 1.62111 | 1.8979 | 0.53313 |
| 5 | C | 0.36051 | 0.9387 | 0.58786 |
| 6 | C | 0.53143 | 0.91906 | -0.99031 |

| | | | | |
|---|---|---|---|---|
| 7 | C | 1.32829 | -1.08169 | -2.17735 |
| 8 | C | 1.0445 | 2.39318 | -0.82636 |
| 9 | C | -0.96021 | 1.51363 | 1.09425 |
| 10 | C | -1.82215 | 0.25733 | 1.24683 |
| 11 | O | -0.92618 | -0.83189 | 1.48324 |
| 12 | C | 0.42208 | -0.36904 | 1.37022 |
| 13 | O | 0.94931 | -0.08781 | 2.6684 |
| 14 | C | 1.15458 | -1.27433 | 3.42271 |
| 15 | C | -2.68695 | 0.00826 | 0.03354 |
| 16 | C | -2.90051 | -1.12908 | -0.66367 |
| 17 | C | -3.7555 | -1.11183 | -1.88117 |
| 18 | O | -4.37374 | 0.06144 | -2.10019 |
| 19 | C | -2.35293 | -2.48117 | -0.30931 |
| 20 | O | -3.91627 | -2.04565 | -2.64752 |
| 21 | O | 3.69232 | 1.06998 | -1.93352 |
| 22 | H | 3.59948 | -0.57964 | -0.74427 |
| 23 | H | 3.78163 | 1.92108 | 0.35321 |
| 24 | H | 3.14792 | 0.53487 | 1.23617 |
| 25 | H | 1.6565 | 2.65242 | 1.32598 |
| 26 | H | -0.38566 | 0.83364 | -1.58139 |
| 27 | H | 0.31073 | -1.29255 | -2.49523 |
| 28 | H | 2.09435 | -1.78735 | -2.48555 |
| 29 | H | 0.2555 | 3.14987 | -0.74259 |
| 30 | H | 1.75973 | 2.76439 | -1.56612 |
| 31 | H | -1.41017 | 2.27518 | 0.45056 |
| 32 | H | -0.80688 | 1.96956 | 2.08083 |
| 33 | H | -2.48418 | 0.36271 | 2.1137 |
| 34 | H | 1.02967 | -1.13984 | 0.88741 |
| 35 | H | 0.20874 | -1.79667 | 3.59407 |
| 36 | H | 1.86496 | -1.93455 | 2.91579 |
| 37 | H | 1.57441 | -0.99416 | 4.39262 |
| 38 | H | -3.19375 | 0.91406 | -0.30058 |
| 39 | H | -4.88695 | -0.10296 | -2.91812 |
| 40 | H | -2.3496 | -2.64573 | 0.77236 |
| 41 | H | -2.9639 | -3.29052 | -0.72326 |
| 42 | H | -1.33623 | -2.59608 | -0.69729 |
| 43 | H | 3.50657 | 0.63374 | -2.78228 |

| **Conformer 1b-4** | | Standard Orientation (Ångstroms) | | |
|---|---|---|---|---|
| I | Atom | X | Y | Z |

| | | | | |
|---|---|---|---|---|
| 1 | C | 1.7022 | 0.08531 | -1.31632 |
| 2 | C | 3.03942 | 0.45281 | -0.71455 |
| 3 | C | 2.89815 | 1.29888 | 0.57008 |
| 4 | C | 1.51705 | 1.98283 | 0.65099 |
| 5 | C | 0.2812 | 0.99069 | 0.624 |
| 6 | C | 0.55379 | 0.97908 | -0.93989 |
| 7 | C | 1.47524 | -1.00167 | -2.06826 |
| 8 | C | 1.0163 | 2.46569 | -0.7425 |
| 9 | C | -1.08497 | 1.52786 | 1.04396 |
| 10 | C | -1.92043 | 0.24791 | 1.13686 |
| 11 | O | -1.01193 | -0.81686 | 1.43136 |
| 12 | C | 0.32743 | -0.31683 | 1.40771 |
| 13 | O | 0.76077 | -0.02466 | 2.73784 |
| 14 | C | 0.94624 | -1.20673 | 3.50405 |
| 15 | C | -2.69021 | -0.02371 | -0.13305 |
| 16 | C | -2.83525 | -1.16749 | -0.83565 |
| 17 | C | -3.6197 | -1.05979 | -2.09096 |
| 18 | O | -3.39133 | -2.08779 | -2.9246 |
| 19 | C | -2.32314 | -2.52148 | -0.45013 |
| 20 | O | -4.37512 | -0.15654 | -2.40297 |
| 21 | O | 3.76279 | 1.21467 | -1.67898 |
| 22 | H | 3.63841 | -0.43878 | -0.49837 |
| 23 | H | 3.68337 | 2.06344 | 0.60951 |
| 24 | H | 3.03141 | 0.65911 | 1.44964 |
| 25 | H | 1.48149 | 2.73621 | 1.44489 |
| 26 | H | -0.32044 | 0.87131 | -1.58934 |
| 27 | H | 0.4849 | -1.23935 | -2.44733 |
| 28 | H | 2.27707 | -1.68786 | -2.325 |
| 29 | H | 0.20383 | 3.20117 | -0.70923 |
| 30 | H | 1.76753 | 2.85719 | -1.43455 |
| 31 | H | -1.51238 | 2.2795 | 0.3738 |
| 32 | H | -1.009 | 1.98383 | 2.03946 |
| 33 | H | -2.64292 | 0.33241 | 1.95637 |
| 34 | H | 0.98643 | -1.06951 | 0.96549 |
| 35 | H | 0.00472 | -1.75297 | 3.61341 |
| 36 | H | 1.70461 | -1.84811 | 3.04469 |
| 37 | H | 1.29468 | -0.91756 | 4.49927 |
| 38 | H | -3.1879 | 0.86905 | -0.51797 |
| 39 | H | -3.95575 | -1.88303 | -3.69862 |
| 40 | H | -2.28712 | -2.65564 | 0.63438 |
| 41 | H | -2.98257 | -3.31706 | -0.81417 |

| | | | | |
|---|---|---|---|---|
| 42 | H | -1.32447 | -2.6865 | -0.86565 |
| 43 | H | 3.63559 | 0.78095 | -2.53967 |

| **Conformer 1b-5** | | Standard Orientation (Ångstroms) | | |
|---|---|---|---|---|
| I | Atom | X | Y | Z |
| 1 | C | 1.97589 | -0.42074 | -1.62305 |
| 2 | C | 3.34627 | -0.1341 | -1.05833 |
| 3 | C | 3.28775 | 0.70223 | 0.2406 |
| 4 | C | 1.94385 | 1.44734 | 0.37737 |
| 5 | C | 0.66209 | 0.51448 | 0.37583 |
| 6 | C | 0.88343 | 0.51739 | -1.19508 |
| 7 | C | 1.67666 | -1.47295 | -2.39871 |
| 8 | C | 1.42214 | 1.97714 | -0.9908 |
| 9 | C | -0.65849 | 1.11864 | 0.8491 |
| 10 | C | -1.54627 | -0.11131 | 1.03105 |
| 11 | O | -0.68539 | -1.25106 | 1.17881 |
| 12 | C | 0.67301 | -0.80827 | 1.13561 |
| 13 | O | 1.15117 | -0.55803 | 2.45957 |
| 14 | C | 1.29012 | -1.7604 | 3.20445 |
| 15 | C | -2.43062 | -0.37843 | -0.15313 |
| 16 | C | -3.73969 | -0.0764 | -0.27408 |
| 17 | C | -4.49089 | -0.43605 | -1.50551 |
| 18 | O | -3.73078 | -0.9707 | -2.47541 |
| 19 | C | -4.56694 | 0.63695 | 0.75488 |
| 20 | O | -5.68641 | -0.26772 | -1.67272 |
| 21 | O | 4.06344 | 0.59034 | -2.05469 |
| 22 | H | 3.8975 | -1.06331 | -0.87386 |
| 23 | H | 4.10769 | 1.42988 | 0.26424 |
| 24 | H | 3.42041 | 0.04486 | 1.10726 |
| 25 | H | 1.9699 | 2.18815 | 1.18344 |
| 26 | H | -0.01719 | 0.46093 | -1.81453 |
| 27 | H | 0.66898 | -1.65225 | -2.76114 |
| 28 | H | 2.44591 | -2.17365 | -2.70935 |
| 29 | H | 0.64717 | 2.74946 | -0.91913 |
| 30 | H | 2.16902 | 2.34407 | -1.70086 |
| 31 | H | -1.09322 | 1.8649 | 0.17701 |
| 32 | H | -0.51253 | 1.604 | 1.82253 |
| 33 | H | -2.13368 | -0.02802 | 1.95016 |
| 34 | H | 1.28998 | -1.57901 | 0.66481 |
| 35 | H | 0.32266 | -2.255 | 3.33154 |

| | | | | |
|---|---|---|---|---|
| 36 | H | 1.99958 | -2.43583 | 2.71663 |
| 37 | H | 1.67951 | -1.50582 | 4.19393 |
| 38 | H | -1.9301 | -0.89329 | -0.972 |
| 39 | H | -4.36914 | -1.14348 | -3.19754 |
| 40 | H | -3.97054 | 1.04943 | 1.5726 |
| 41 | H | -5.09896 | 1.48084 | 0.30172 |
| 42 | H | -5.3057 | -0.04564 | 1.18757 |
| 43 | H | 4.97243 | 0.70045 | -1.73083 |

**Table S7** Calculated $^{1}H$ and $^{13}C$ NMR chemical shifts of compound **1a**.

| Position | Type | Shielding tensors (calculated) [a] | | | | | | | | | Chemical shifts (ppm) (calculated) | Chemical shifts (ppm) (experimental) [b] |
|---|---|---|---|---|---|---|---|---|---|---|---|---|
| | | **1a-1** | **1a-2** | **1a-3** | **1a-4** | **1a-5** | **1a-6** | **1a-7** | **1a-8** | **1a** (averaged) | **1a** (unscaled) | Brasilterpene A (**1**) |
| 1 | C | 125.4239 | 125.5415 | 125.7861 | 125.5595 | 125.8964 | 123.1487 | 126.0792 | 125.6667 | 125.66054 | 58.30626 | 51.1 |
| 2 | C | 15.2609 | 15.2814 | 15.1319 | 18.3104 | 15.1342 | 12.082 | 14.6294 | 18.2888 | 16.011035 | 167.9558 | 153.8 |
| 3 | C | 109.7372 | 109.7596 | 108.7418 | 110.5339 | 108.7659 | 112.1642 | 110.4392 | 110.604 | 109.50122 | 74.46558 | 64.8 |
| 4 | C | 145.6046 | 145.6318 | 142.5551 | 142.7832 | 142.5691 | 140.4954 | 146.2924 | 142.8086 | 143.40976 | 40.55704 | 35.9 |
| 5 | C | 137.157 | 137.1378 | 136.7552 | 137.0139 | 136.749 | 134.0384 | 137.4473 | 136.9886 | 136.91856 | 47.04824 | 38.7 |
| 6 | C | 119.4322 | 119.3673 | 119.4518 | 119.4431 | 119.3804 | 122.1254 | 121.4862 | 119.4026 | 119.43455 | 64.53225 | 54.2 |
| 7 | C | 154.4205 | 154.4274 | 154.5381 | 154.2583 | 154.537 | 145.2376 | 154.4048 | 154.2429 | 154.41029 | 29.55651 | 29.1 |
| 8 | C | 138.3557 | 138.5902 | 138.2513 | 138.3947 | 138.4876 | 131.9963 | 138.9393 | 138.6483 | 138.37767 | 45.58913 | 40.8 |
| 9 | C | 103.8199 | 104.1436 | 103.8039 | 103.796 | 104.1304 | 103.6212 | 99.5351 | 104.1424 | 103.90154 | 80.06526 | 73.0 |
| 10 | C | 26.3189 | 25.7979 | 26.4523 | 26.3278 | 25.9359 | 28.7055 | 27.2426 | 25.8343 | 26.233146 | 157.7337 | 142.1 |
| 11 | C | 48.8347 | 48.6309 | 48.7763 | 48.8337 | 48.5797 | 46.4075 | 52.4568 | 48.6089 | 48.743062 | 135.2237 | 128.3 |
| 12 | C | 5.5818 | 5.8329 | 5.6114 | 5.5623 | 5.8495 | 6.1544 | 4.1953 | 5.816 | 5.6655228 | 178.3013 | 168.6 |
| 13 | C | 168.98 | 168.7373 | 168.9734 | 168.9844 | 168.7321 | 168.2854 | 169.2291 | 168.7463 | 168.90263 | 15.06417 | 12.7 |
| 14 | C | 70.6988 | 70.7011 | 70.7616 | 70.5488 | 70.7708 | 68.6866 | 70.7416 | 70.553 | 70.68539 | 113.2814 | 105.7 |
| 15 | C | 71.9598 | 71.965 | 70.4512 | 69.989 | 70.4418 | 63.3071 | 71.6801 | 69.943 | 70.696694 | 113.2701 | 110.7 |
| 16 | C | 126.7779 | 126.8001 | 126.8118 | 126.8016 | 126.8332 | 127.8321 | 126.8028 | 126.8315 | 126.81002 | 57.15678 | 54.0 |
| 1 | H | 28.9229 | 28.9162 | 28.9171 | 26.2931 | 28.9097 | 28.9603 | 29.1244 | 28.9338 | 28.439902 | 3.428998 | 2.61 |
| 3 | H | 26.9561 | 26.952 | 27.1632 | 26.856 | 27.1618 | 27.5008 | 26.969 | 26.852 | 27.02693 | 4.84197 | 4.28 |

| | | | | | | | | | | | | |
|---|---|---|---|---|---|---|---|---|---|---|---|---|
| 4a | H | 29.9663 | 29.9094 | 30.1471 | 29.9801 | 30.1341 | 29.49573 | 29.9375 | 29.9431 | 30.044455 | 1.824445 | 1.64 |
| 4b | H | 29.48105 | 29.48675 | 29.3968 | 28.9394 | 29.4043 | 29.49573 | 29.4321 | 29.5039 | 29.346061 | 2.522839 | 2.38 |
| 5 | H | 29.48105 | 29.48675 | 29.4741 | 26.6039 | 29.4772 | 29.1733 | 29.5075 | 29.5039 | 28.954834 | 2.914066 | 2.23 |
| 7a | H | 30.3533 | 30.3655 | 30.3432 | 27.1796 | 30.3555 | 30.975 | 30.3209 | 30.3762 | 29.775356 | 2.093544 | 1.55 |
| 7b | H | 29.8698 | 29.9094 | 29.87597 | 26.6814 | 29.8655 | 29.5882 | 29.799 | 29.8327 | 29.288273 | 2.580627 | 2.17 |
| 8a | H | 29.8698 | 29.9094 | 29.87597 | 28.3984 | 29.9312 | 29.89515 | 29.7054 | 29.9431 | 29.62136 | 2.24754 | 1.97 |
| 8b | H | 29.2577 | 29.2827 | 29.2703 | 28.161 | 29.2956 | 29.49573 | 29.3235 | 29.2943 | 29.072836 | 2.796064 | 2.42 |
| 9 | H | 26.68777 | 26.72285 | 26.6901 | 28.789 | 26.6982 | 26.7931 | 26.5135 | 26.7052 | 27.07771 | 4.79119 | 4.81 |
| 10 | H | 24.5368 | 24.5057 | 24.5564 | 30.5027 | 24.5249 | 24.6917 | 24.5797 | 24.5007 | 25.625245 | 6.243655 | 6.60 |
| 13 | H | 30.1589 | 29.9094 | 30.0437 | 29.50045 | 29.9312 | 30.0079 | 30.3209 | 29.9431 | 29.930557 | 1.938343 | 1.77 |
| 13 | H | 30.0352 | 29.7595 | 29.87597 | 29.86437 | 29.7588 | 29.89515 | 28.6606 | 29.7537 | 29.864679 | 2.004221 | 1.77 |
| 13 | H | 29.8698 | 30.0418 | 30.1471 | 29.86437 | 30.046 | 30.1019 | 30.2414 | 30.0411 | 30.01306 | 1.85584 | 1.77 |
| 14 | H | 27.1863 | 27.1925 | 27.299 | 24.5303 | 27.3044 | 27.0488 | 27.1237 | 27.1869 | 26.75539 | 5.11351 | 4.37 |
| 15a | H | 26.68777 | 26.6371 | 26.6901 | 30.3593 | 26.6982 | 26.46315 | 26.7508 | 26.5973 | 27.34815 | 4.52075 | 4.80 |
| 15b | H | 26.68777 | 26.72285 | 26.3376 | 29.2675 | 26.3381 | 26.46315 | 26.6602 | 26.2974 | 26.962689 | 4.906211 | 5.03 |
| 16 | H | 28.3938 | 28.3968 | 28.3954 | 25.4785 | 28.399 | 28.3303 | 28.4392 | 28.4017 | 27.863805 | 4.005095 | 3.20 |
| 16 | H | 28.1628 | 28.1657 | 28.1723 | 30.033 | 28.1759 | 28.176 | 28.2241 | 28.1648 | 28.50965 | 3.35925 | 3.20 |
| 16 | H | 28.7907 | 28.7926 | 28.8325 | 30.159 | 28.8333 | 28.8594 | 28.7814 | 28.7901 | 29.060435 | 2.808465 | 3.20 |
| 3-OH | H | 29.8698 | 29.9094 | 31.2024 | 29.86437 | 31.2042 | 27.9855 | 29.8797 | 30.5055 | 30.548603 | 1.320297 | 4.90 |
| 12-COOH | H | 25.4851 | 25.3674 | 25.489 | 29.50045 | 25.3622 | 25.4467 | 25.5869 | 25.3639 | 26.18195 | 5.68695 | 12.40 |

[a] Calculated using the GIAO method at the B3LYP/6-311+G(d,p) level in DMSO (PCM). [b] Measured in DMSO-$d_6$ (400 and 100 MHz).

**Table S8** Calculated $^{1}H$ and $^{13}C$ NMR chemical shifts of compound **1b**.

| Position | Type | Shielding tensors (calculated) [a] | | | | | | Chemical shifts (ppm) (calculated) | Chemical shifts (ppm) (experimental) [b] |
|---|---|---|---|---|---|---|---|---|---|
| | | **1b-1** | **1b-2** | **1b-3** | **1b-4** | **1b-5** | **1b** (averaged) | **1b** (unscaled) | Brasilterpene A (**1**) |
| 1 | C | 126.8673 | 126.9803 | 127.1546 | 127.0295 | 126.4992 | 126.8703 | 57.09645 | 51.1 |
| 2 | C | 14.5307 | 14.5615 | 14.2979 | 14.3107 | 17.6692 | 14.86201 | 169.1048 | 153.8 |
| 3 | C | 109.1947 | 109.1953 | 109.2252 | 109.2782 | 109.2818 | 109.2051 | 74.76175 | 64.8 |
| 4 | C | 146.2069 | 146.2703 | 146.3825 | 146.4152 | 141.7258 | 145.7646 | 38.2022 | 35.9 |
| 5 | C | 138.1753 | 138.1976 | 138.9825 | 138.9603 | 138.3587 | 138.2198 | 45.74704 | 38.7 |
| 6 | C | 119.9326 | 119.8808 | 122.1739 | 122.2038 | 120.3351 | 120.0097 | 63.95715 | 54.2 |
| 7 | C | 153.6784 | 153.6837 | 153.402 | 153.3337 | 153.8263 | 153.6886 | 30.27816 | 29.1 |
| 8 | C | 138.029 | 138.2677 | 138.9138 | 138.9603 | 138.1575 | 138.1384 | 45.82835 | 40.8 |
| 9 | C | 103.6849 | 103.9735 | 99.1035 | 98.6802 | 103.5881 | 103.6581 | 80.30872 | 73.0 |
| 10 | C | 26.3264 | 25.7646 | 26.7619 | 26.6285 | 26.2996 | 26.15496 | 157.8118 | 142.1 |
| 11 | C | 48.9163 | 48.7574 | 52.2765 | 51.7789 | 48.945 | 48.94226 | 135.0245 | 128.3 |
| 12 | C | 5.8509 | 5.9364 | 4.4983 | 4.2794 | 5.8397 | 5.844187 | 178.1226 | 168.6 |
| 13 | C | 168.909 | 168.6527 | 169.2185 | 169.2191 | 168.9243 | 168.8366 | 15.13015 | 12.7 |
| 14 | C | 70.5191 | 70.4922 | 70.6361 | 70.6643 | 70.4391 | 70.50515 | 113.4616 | 105.7 |
| 15 | C | 64.6031 | 64.5641 | 64.4498 | 64.4925 | 61.967 | 64.31309 | 119.6537 | 110.7 |
| 16 | C | 126.7121 | 126.7311 | 126.8201 | 126.8172 | 126.7146 | 126.7208179 | 57.24598 | 54.0 |
| 1 | H | 28.9706 | 28.9621 | 29.0822 | 29.0979 | 28.9378 | 28.96717 | 2.901732 | 2.61 |
| 3 | H | 27.3027 | 27.3054 | 27.32535 | 27.3292 | 27.2047 | 27.2939 | 4.575004 | 4.28 |
| 4a | H | 29.9672 | 29.9662 | 29.9021 | 29.9076 | 30.05857 | 29.97495 | 1.893952 | 1.64 |
| 4b | H | 29.4105 | 29.4134 | 29.4036 | 29.4065 | 29.2672 | 29.39636 | 2.472541 | 2.38 |

| | | | | | | | | | |
|---|---|---|---|---|---|---|---|---|---|
| 5 | H | 29.5511 | 29.5513 | 29.5679 | 29.5731 | 29.5348 | 29.54989 | 2.319011 | 2.23 |
| 7a | H | 30.0433 | 30.0495 | 29.7994 | 29.7921 | 30.05857 | 30.04121 | 1.827686 | 1.55 |
| 7b | H | 29.779 | 29.77775 | 29.6869 | 29.6787 | 29.747 | 29.7731 | 2.095796 | 2.17 |
| 8a | H | 29.88015 | 29.8918 | 29.6301 | 29.6787 | 29.87105 | 29.87752 | 1.991377 | 1.97 |
| 8b | H | 29.2737 | 29.2922 | 29.2559 | 29.2725 | 29.2672 | 29.27859 | 2.590312 | 2.42 |
| 9 | H | 26.7284 | 26.7465 | 26.55035 | 26.5403 | 26.7176 | 26.72885 | 5.140051 | 4.81 |
| 10 | H | 24.5327 | 24.5157 | 24.5461 | 24.5367 | 24.5153 | 24.52575 | 7.34315 | 6.60 |
| 13 | H | 30.1701 | 29.9662 | 28.6329 | 28.5494 | 29.87105 | 30.03869 | 1.830207 | 1.77 |
| 13 | H | 30.0433 | 29.77775 | 30.2284 | 30.15805 | 30.1651 | 29.97574 | 1.893163 | 1.77 |
| 13 | H | 29.88015 | 30.0495 | 30.3226 | 30.15805 | 30.05857 | 29.96118 | 1.907722 | 1.77 |
| 14 | H | 27.4065 | 27.4065 | 27.32535 | 27.3292 | 27.3811 | 27.40203 | 4.466874 | 4.37 |
| 15a | H | 26.5707 | 26.5637 | 26.6891 | 26.6678 | 26.41525 | 26.55485 | 5.314048 | 4.80 |
| 15b | H | 26.4806 | 26.4839 | 26.55035 | 26.5403 | 26.41525 | 26.47636 | 5.392539 | 5.03 |
| 16 | H | 28.1887 | 28.1955 | 28.2514 | 28.25 | 28.1825 | 28.19163 | 3.677271 | 3.20 |
| 16 | H | 28.8494 | 28.8504 | 28.8373 | 28.8362 | 28.8483 | 28.84932 | 3.019583 | 3.20 |
| 16 | H | 28.3926 | 28.3971 | 28.4479 | 28.4433 | 28.4006 | 28.39609 | 3.472813 | 3.20 |
| 3-OH | H | 30.9158 | 30.9251 | 31.0301 | 31.0394 | 30.5954 | 30.88806 | 0.980837 | 4.90 |
| 12-COOH | H | 25.5066 | 25.36 | 25.6084 | 25.4144 | 25.4969 | 25.46013 | 6.40877 | 12.40 |

[a] Calculated using the GIAO method at the B3LYP/6-311+G(d,p) level in DMSO (PCM). [b] Measured in DMSO-$d_6$ (400 and 100 MHz).

**Figure S45** Correlation plots of experimental $^{13}C$ NMR chemical shifts of brasilterpene A (**1**) corresponding calculated $^{13}C$ NMR chemical shifts of **1a** and **1b**.

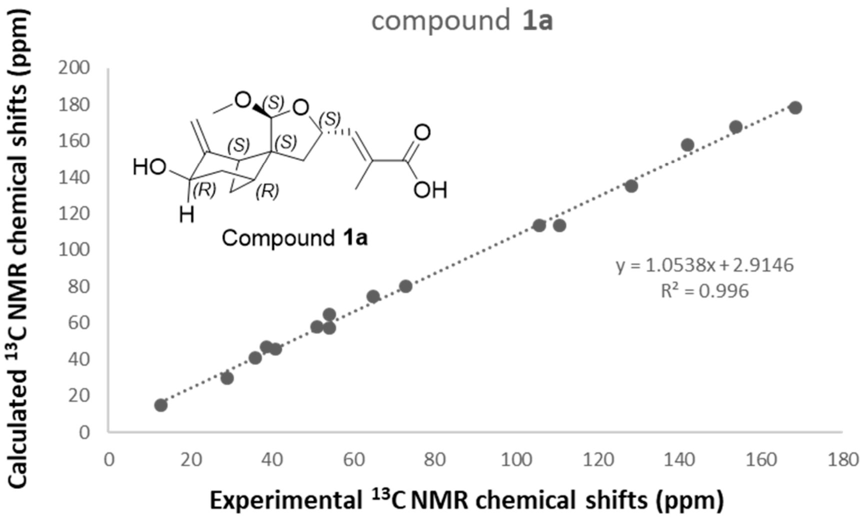


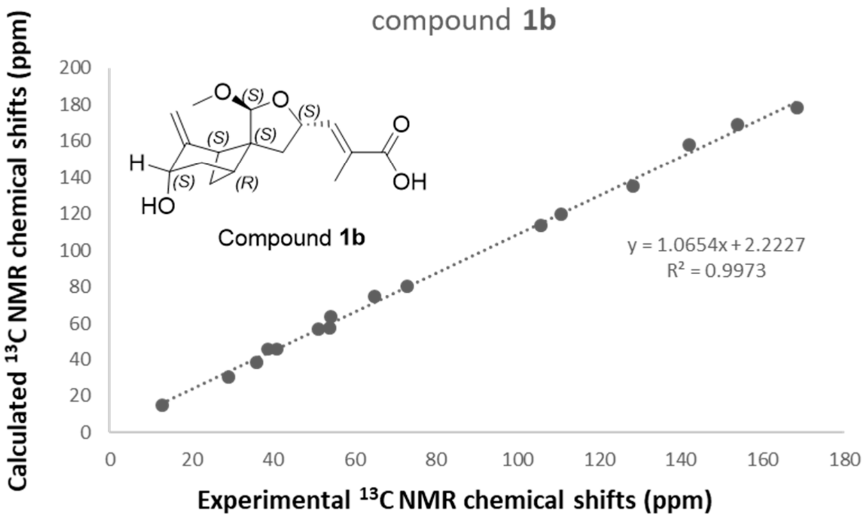

**Table S9** DP4+ analysis using the calculated shielding tensors for compound **1a** and **1b**.

| Functional | Solvent? | | Basis Set | | Type of Data | |
|---|---|---|---|---|---|---|
| B3LYP | PCM | | 6-311+G(d,p) | | Shielding Tensors | |
| | Isomer 1 | Isomer 2 | Isomer 3 | Isomer 4 | Isomer 5 | Isomer 6 |
| sDP4+ (H data) | 0.00% | 100.00% | – | – | – | – |
| sDP4+ (C data) | 0.89% | 99.11% | – | – | – | – |
| sDP4+ (all data) | 0.00% | 100.00% | – | – | – | – |
| uDP4+ (H data) | 0.00% | 100.00% | – | – | – | – |
| uDP4+ (C data) | 4.59% | 95.41% | – | – | – | – |
| uDP4+ (all data) | 0.00% | 100.00% | – | – | – | – |
| DP4+ (H data) | 0.00% | 100.00% | – | – | – | – |
| DP4+ (C data) | 0.04% | 99.96% | – | – | – | – |
| DP4+ (all data) | 0.00% | 100.00% | – | – | – | – |

[a] sDP4+: scaled DP4+; [b] uDP4+: unscaled DP4+.

**Figure S46** DFT-optimized structures for low-energy conformers of **1** (1*S*,3*S*,5*R*,6*S*,9*S*,14*S*-**1**) at B3LYP/6-31+G(d) level in MeOH (PCM).

Conformer populations were calculated using the Gibbs free energy and Boltzmann population at 298 K estimated thereof.

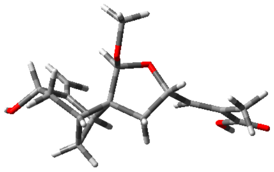

Conf. 1-1 56.0%

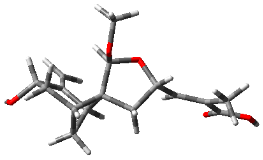

Conf. 1-2 31.8%

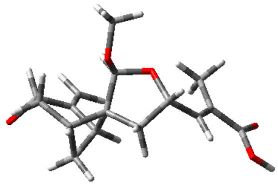

Conf. 1-3 1.5%

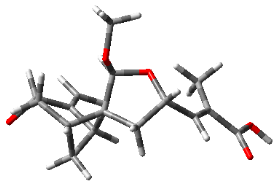

Conf. 1-4 0.7%

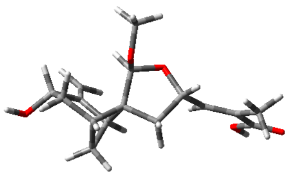

Conf. 1-5 9.9%

**Table S10** Harmonic frequencies (cm**$^{-1}$) of compound **1**.

| **Conformations** | **Frequencies** | | |
|---|---|---|---|
| | 1 | 2 | 3 |
| 1-1 | 21.6203 | 26.3272 | 51.6263 |
| 1-2 | 21.9977 | 26.2790 | 51.6923 |
| 1-3 | 11.6008 | 24.9585 | 56.8813 |
| 1-4 | 12.8130 | 26.3628 | 57.0150 |
| 1-5 | 20.1432 | 25.6184 | 51.2415 |

**Table S11** Important thermodynamic parameters (a.u.) and Boltzmann distributions of the optimized compound **1** at B3LYP/6-31G+(d) level of theory with PCM solvent model for MeOH.

| Conformations | E+ZPE | G | % |
|---|---|---|---|
| 1-1 | -998.681656 | -998.732395 | 56.0% |
| 1-2 | -998.681260 | -998.731860 | 31.8% |
| 1-3 | -998.677686 | -998.728998 | 1.5% |
| 1-4 | -998.677348 | -998.728302 | 0.7% |
| 1-5 | -998.679895 | -998.730760 | 9.9% |

E+ZPE, G: total energy with zero point energy (ZPE) and Gibbs free energy at B3LYP/6-31G+(d) level of theory with PCM solvent model for MeOH. %: Boltzmann distributions, using the relative Gibbs free energies as weighting factors

**Table S12** Cartesian coordinates of the low-energy re-optimized conformers of compound **1** (1*S*,3*S*,5*R*,6*S*,9*S*,14*S*-**1**) calculated at B3LYP/6-31+G(d) level of theory with PCM solvent model for MeOH.

| **Conformer 1-1** | | Standard Orientation (Ångstroms) | | |
|---|---|---|---|---|
| I | Atom | X | Y | Z |
| 1 | C | 2.54326 | -0.28775 | -0.45061 |
| 2 | C | 3.47355 | 0.39018 | 0.5294 |
| 3 | C | 2.74563 | 1.42362 | 1.41713 |
| 4 | C | 1.41948 | 1.88673 | 0.77813 |
| 5 | C | 0.37884 | 0.72555 | 0.49251 |
| 6 | C | 1.28748 | 0.46869 | -0.78289 |
| 7 | C | 2.72638 | -1.51813 | -0.95159 |
| 8 | C | 1.52448 | 2.0202 | -0.77012 |
| 9 | C | -1.06647 | 1.11706 | 0.19013 |
| 10 | C | -1.80834 | -0.21403 | 0.30836 |
| 11 | O | -0.98943 | -1.10142 | 1.08646 |
| 12 | C | 0.17552 | -0.39095 | 1.51157 |

| | | | | |
|---|---|---|---|---|
| 13 | O | -0.05196 | 0.20649 | 2.78973 |
| 14 | C | -0.13403 | -0.76891 | 3.81987 |
| 15 | C | -2.0365 | -0.87835 | -1.01915 |
| 16 | C | -3.17689 | -0.8834 | -1.73951 |
| 17 | C | -3.25631 | -1.61807 | -3.0293 |
| 18 | O | -2.09009 | -2.15101 | -3.43042 |
| 19 | C | -4.44626 | -0.1762 | -1.3645 |
| 20 | O | -4.26212 | -1.74137 | -3.70648 |
| 21 | O | 4.48256 | 1.06743 | -0.21679 |
| 22 | H | 3.98435 | -0.34038 | 1.16644 |
| 23 | H | 3.38812 | 2.29541 | 1.58981 |
| 24 | H | 2.53364 | 0.9836 | 2.39812 |
| 25 | H | 1.00239 | 2.75855 | 1.29305 |
| 26 | H | 0.77729 | 0.10625 | -1.68086 |
| 27 | H | 2.00641 | -1.97572 | -1.62471 |
| 28 | H | 3.60408 | -2.10577 | -0.69836 |
| 29 | H | 0.72753 | 2.61271 | -1.23495 |
| 30 | H | 2.47026 | 2.39039 | -1.17627 |
| 31 | H | -1.22028 | 1.60888 | -0.77533 |
| 32 | H | -1.43185 | 1.80279 | 0.96518 |
| 33 | H | -2.7493 | -0.0824 | 0.85063 |
| 34 | H | 1.01919 | -1.08403 | 1.5765 |
| 35 | H | -0.97919 | -1.44308 | 3.65258 |
| 36 | H | 0.79776 | -1.339 | 3.88522 |
| 37 | H | -0.28943 | -0.25021 | 4.76975 |
| 38 | H | -1.17242 | -1.42388 | -1.39535 |
| 39 | H | -2.31975 | -2.58808 | -4.27618 |
| 40 | H | -4.805 | 0.43656 | -2.19899 |
| 41 | H | -5.22445 | -0.90301 | -1.10939 |
| 42 | H | -4.32464 | 0.49906 | -0.51375 |
| 43 | H | 4.76649 | 0.46501 | -0.92504 |

| **Conformer 1-2** | | Standard Orientation (Ångstroms) | | |
|---|---|---|---|---|
| I | Atom | X | Y | Z |
| 1 | C | 2.48443 | 0.72767 | -0.48921 |
| 2 | C | 3.24833 | 1.3309 | 0.66733 |
| 3 | C | 2.32509 | 1.74018 | 1.83619 |
| 4 | C | 0.86463 | 1.91889 | 1.37037 |
| 5 | C | 0.21513 | 0.63144 | 0.71149 |
| 6 | C | 1.02956 | 1.0963 | -0.56888 |

| | | | | |
|---|---|---|---|---|
| 7 | C | 2.99175 | -0.1535 | -1.36357 |
| 8 | C | 0.77107 | 2.55425 | -0.04841 |
| 9 | C | -1.30223 | 0.60214 | 0.53781 |
| 10 | C | -1.575 | -0.86497 | 0.20797 |
| 11 | O | -0.4543 | -1.63176 | 0.67517 |
| 12 | C | 0.4706 | -0.74742 | 1.31207 |
| 13 | O | 0.19922 | -0.68134 | 2.7138 |
| 14 | C | 0.53209 | -1.89591 | 3.37208 |
| 15 | C | -1.69695 | -1.12255 | -1.26606 |
| 16 | C | -2.83247 | -1.29542 | -1.97253 |
| 17 | C | -2.66927 | -1.56202 | -3.42402 |
| 18 | O | -3.83085 | -1.88421 | -4.01609 |
| 19 | C | -4.22593 | -1.21655 | -1.42793 |
| 20 | O | -1.63362 | -1.51188 | -4.06337 |
| 21 | O | 3.91938 | 2.49659 | 0.1933 |
| 22 | H | 4.02037 | 0.64664 | 1.03635 |
| 23 | H | 2.67742 | 2.6747 | 2.28916 |
| 24 | H | 2.35818 | 0.97369 | 2.61865 |
| 25 | H | 0.24899 | 2.40044 | 2.13735 |
| 26 | H | 0.5729 | 0.88694 | -1.54128 |
| 27 | H | 2.38797 | -0.58584 | -2.15731 |
| 28 | H | 4.02921 | -0.47028 | -1.30878 |
| 29 | H | -0.21231 | 2.96744 | -0.30242 |
| 30 | H | 1.5099 | 3.3199 | -0.30221 |
| 31 | H | -1.69523 | 1.29078 | -0.21649 |
| 32 | H | -1.78627 | 0.85309 | 1.49026 |
| 33 | H | -2.45909 | -1.22076 | 0.74508 |
| 34 | H | 1.48935 | -1.11245 | 1.15297 |
| 35 | H | -0.07147 | -2.72477 | 2.99064 |
| 36 | H | 1.59721 | -2.11789 | 3.25474 |
| 37 | H | 0.31922 | -1.7762 | 4.43784 |
| 38 | H | -0.74227 | -1.19479 | -1.78806 |
| 39 | H | -3.57604 | -2.03821 | -4.9494 |
| 40 | H | -4.7007 | -2.20291 | -1.44904 |
| 41 | H | -4.26039 | -0.85228 | -0.39792 |
| 42 | H | -4.83255 | -0.524 | -2.0217 |
| 43 | H | 4.29493 | 2.27593 | -0.67596 |

| **Conformer 1-3** | | Standard Orientation (Ångstroms) | | |
|---|---|---|---|---|
| I | Atom | X | Y | Z |

| | | | | |
|---|---|---|---|---|
| 1 | C | 1.62992 | -0.0037 | -1.43887 |
| 2 | C | 3.01196 | 0.32763 | -0.92357 |
| 3 | C | 2.97556 | 1.17759 | 0.36561 |
| 4 | C | 1.62111 | 1.8979 | 0.53313 |
| 5 | C | 0.36051 | 0.9387 | 0.58786 |
| 6 | C | 0.53143 | 0.91906 | -0.99031 |
| 7 | C | 1.32829 | -1.08169 | -2.17735 |
| 8 | C | 1.0445 | 2.39318 | -0.82636 |
| 9 | C | -0.96021 | 1.51363 | 1.09425 |
| 10 | C | -1.82215 | 0.25733 | 1.24683 |
| 11 | O | -0.92618 | -0.83189 | 1.48324 |
| 12 | C | 0.42208 | -0.36904 | 1.37022 |
| 13 | O | 0.94931 | -0.08781 | 2.6684 |
| 14 | C | 1.15458 | -1.27433 | 3.42271 |
| 15 | C | -2.68695 | 0.00826 | 0.03354 |
| 16 | C | -2.90051 | -1.12908 | -0.66367 |
| 17 | C | -3.7555 | -1.11183 | -1.88117 |
| 18 | O | -4.37374 | 0.06144 | -2.10019 |
| 19 | C | -2.35293 | -2.48117 | -0.30931 |
| 20 | O | -3.91627 | -2.04565 | -2.64752 |
| 21 | O | 3.69232 | 1.06998 | -1.93352 |
| 22 | H | 3.59948 | -0.57964 | -0.74427 |
| 23 | H | 3.78163 | 1.92108 | 0.35321 |
| 24 | H | 3.14792 | 0.53487 | 1.23617 |
| 25 | H | 1.6565 | 2.65242 | 1.32598 |
| 26 | H | -0.38566 | 0.83364 | -1.58139 |
| 27 | H | 0.31073 | -1.29255 | -2.49523 |
| 28 | H | 2.09435 | -1.78735 | -2.48555 |
| 29 | H | 0.2555 | 3.14987 | -0.74259 |
| 30 | H | 1.75973 | 2.76439 | -1.56612 |
| 31 | H | -1.41017 | 2.27518 | 0.45056 |
| 32 | H | -0.80688 | 1.96956 | 2.08083 |
| 33 | H | -2.48418 | 0.36271 | 2.1137 |
| 34 | H | 1.02967 | -1.13984 | 0.88741 |
| 35 | H | 0.20874 | -1.79667 | 3.59407 |
| 36 | H | 1.86496 | -1.93455 | 2.91579 |
| 37 | H | 1.57441 | -0.99416 | 4.39262 |
| 38 | H | -3.19375 | 0.91406 | -0.30058 |
| 39 | H | -4.88695 | -0.10296 | -2.91812 |
| 40 | H | -2.3496 | -2.64573 | 0.77236 |
| 41 | H | -2.9639 | -3.29052 | -0.72326 |

| | | | | |
|---|---|---|---|---|
| 42 | H | -1.33623 | -2.59608 | -0.69729 |
| 43 | H | 3.50657 | 0.63374 | -2.78228 |

| **Conformer 1-4** | | Standard Orientation (Ångstroms) | | |
|---|---|---|---|---|
| I | Atom | X | Y | Z |
| 1 | C | 1.7022 | 0.08531 | -1.31632 |
| 2 | C | 3.03942 | 0.45281 | -0.71455 |
| 3 | C | 2.89815 | 1.29888 | 0.57008 |
| 4 | C | 1.51705 | 1.98283 | 0.65099 |
| 5 | C | 0.2812 | 0.99069 | 0.624 |
| 6 | C | 0.55379 | 0.97908 | -0.93989 |
| 7 | C | 1.47524 | -1.00167 | -2.06826 |
| 8 | C | 1.0163 | 2.46569 | -0.7425 |
| 9 | C | -1.08497 | 1.52786 | 1.04396 |
| 10 | C | -1.92043 | 0.24791 | 1.13686 |
| 11 | O | -1.01193 | -0.81686 | 1.43136 |
| 12 | C | 0.32743 | -0.31683 | 1.40771 |
| 13 | O | 0.76077 | -0.02466 | 2.73784 |
| 14 | C | 0.94624 | -1.20673 | 3.50405 |
| 15 | C | -2.69021 | -0.02371 | -0.13305 |
| 16 | C | -2.83525 | -1.16749 | -0.83565 |
| 17 | C | -3.6197 | -1.05979 | -2.09096 |
| 18 | O | -3.39133 | -2.08779 | -2.9246 |
| 19 | C | -2.32314 | -2.52148 | -0.45013 |
| 20 | O | -4.37512 | -0.15654 | -2.40297 |
| 21 | O | 3.76279 | 1.21467 | -1.67898 |
| 22 | H | 3.63841 | -0.43878 | -0.49837 |
| 23 | H | 3.68337 | 2.06344 | 0.60951 |
| 24 | H | 3.03141 | 0.65911 | 1.44964 |
| 25 | H | 1.48149 | 2.73621 | 1.44489 |
| 26 | H | -0.32044 | 0.87131 | -1.58934 |
| 27 | H | 0.4849 | -1.23935 | -2.44733 |
| 28 | H | 2.27707 | -1.68786 | -2.325 |
| 29 | H | 0.20383 | 3.20117 | -0.70923 |
| 30 | H | 1.76753 | 2.85719 | -1.43455 |
| 31 | H | -1.51238 | 2.2795 | 0.3738 |
| 32 | H | -1.009 | 1.98383 | 2.03946 |
| 33 | H | -2.64292 | 0.33241 | 1.95637 |
| 34 | H | 0.98643 | -1.06951 | 0.96549 |
| 35 | H | 0.00472 | -1.75297 | 3.61341 |

| | | | | |
|---|---|---|---|---|
| 36 | H | 1.70461 | -1.84811 | 3.04469 |
| 37 | H | 1.29468 | -0.91756 | 4.49927 |
| 38 | H | -3.1879 | 0.86905 | -0.51797 |
| 39 | H | -3.95575 | -1.88303 | -3.69862 |
| 40 | H | -2.28712 | -2.65564 | 0.63438 |
| 41 | H | -2.98257 | -3.31706 | -0.81417 |
| 42 | H | -1.32447 | -2.6865 | -0.86565 |
| 43 | H | 3.63559 | 0.78095 | -2.53967 |

| **Conformer 1-5** | | Standard Orientation (Ångstroms) | | |
|---|---|---|---|---|
| I | Atom | X | Y | Z |
| 1 | C | 1.97589 | -0.42074 | -1.62305 |
| 2 | C | 3.34627 | -0.1341 | -1.05833 |
| 3 | C | 3.28775 | 0.70223 | 0.2406 |
| 4 | C | 1.94385 | 1.44734 | 0.37737 |
| 5 | C | 0.66209 | 0.51448 | 0.37583 |
| 6 | C | 0.88343 | 0.51739 | -1.19508 |
| 7 | C | 1.67666 | -1.47295 | -2.39871 |
| 8 | C | 1.42214 | 1.97714 | -0.9908 |
| 9 | C | -0.65849 | 1.11864 | 0.8491 |
| 10 | C | -1.54627 | -0.11131 | 1.03105 |
| 11 | O | -0.68539 | -1.25106 | 1.17881 |
| 12 | C | 0.67301 | -0.80827 | 1.13561 |
| 13 | O | 1.15117 | -0.55803 | 2.45957 |
| 14 | C | 1.29012 | -1.7604 | 3.20445 |
| 15 | C | -2.43062 | -0.37843 | -0.15313 |
| 16 | C | -3.73969 | -0.0764 | -0.27408 |
| 17 | C | -4.49089 | -0.43605 | -1.50551 |
| 18 | O | -3.73078 | -0.9707 | -2.47541 |
| 19 | C | -4.56694 | 0.63695 | 0.75488 |
| 20 | O | -5.68641 | -0.26772 | -1.67272 |
| 21 | O | 4.06344 | 0.59034 | -2.05469 |
| 22 | H | 3.8975 | -1.06331 | -0.87386 |
| 23 | H | 4.10769 | 1.42988 | 0.26424 |
| 24 | H | 3.42041 | 0.04486 | 1.10726 |
| 25 | H | 1.9699 | 2.18815 | 1.18344 |
| 26 | H | -0.01719 | 0.46093 | -1.81453 |
| 27 | H | 0.66898 | -1.65225 | -2.76114 |
| 28 | H | 2.44591 | -2.17365 | -2.70935 |
| 29 | H | 0.64717 | 2.74946 | -0.91913 |

| | | | | |
|---|---|---|---|---|
| 30 | H | 2.16902 | 2.34407 | -1.70086 |
| 31 | H | -1.09322 | 1.8649 | 0.17701 |
| 32 | H | -0.51253 | 1.604 | 1.82253 |
| 33 | H | -2.13368 | -0.02802 | 1.95016 |
| 34 | H | 1.28998 | -1.57901 | 0.66481 |
| 35 | H | 0.32266 | -2.255 | 3.33154 |
| 36 | H | 1.99958 | -2.43583 | 2.71663 |
| 37 | H | 1.67951 | -1.50582 | 4.19393 |
| 38 | H | -1.9301 | -0.89329 | -0.972 |
| 39 | H | -4.36914 | -1.14348 | -3.19754 |
| 40 | H | -3.97054 | 1.04943 | 1.5726 |
| 41 | H | -5.09896 | 1.48084 | 0.30172 |
| 42 | H | -5.3057 | -0.04564 | 1.18757 |
| 43 | H | 4.97243 | 0.70045 | -1.73083 |

**Figure S47** DFT-optimized structures for low-energy conformers of **2** (1*S*,3*S*,5*R*,6*S*,9*S*,14*R*-**2**) at B3LYP/6-31+G(d) level in MeOH (PCM).

Conformer populations were calculated using the Gibbs free energy and Boltzmann population at 298 K estimated thereof.

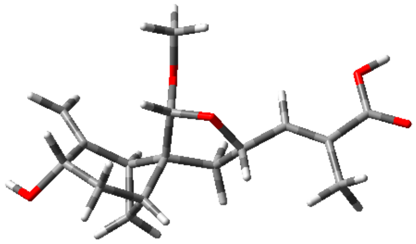

Conf. 2-1 41.06%

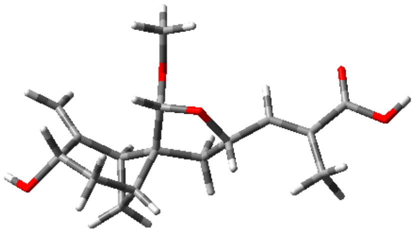

Conf. 2-2 17.8%

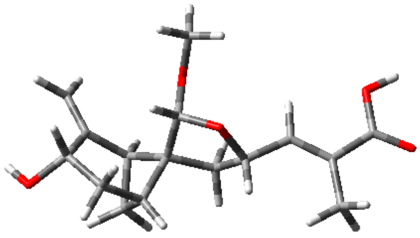

Conf. 2-3 41.1%

**Table S13** Harmonic frequencies (cm**$^{-1}$) of compound **2**.

| **Conformations** | **Frequencies** | | |
|---|---|---|---|
| | 1 | 2 | 3 |

| | | | |
|---|---|---|---|
| 2-1 | 24.2970 | 30.1406 | 44.1924 |
| 2-2 | 26.0256 | 31.5825 | 45.6885 |
| 2-3 | 24.3081 | 30.1579 | 44.1860 |

**Table S14** Important thermodynamic parameters (a.u.) and Boltzmann distributions of the optimized compound **2** at B3LYP/6-31G+(d) level of theory with PCM solvent model for MeOH.

| Conformations | E+ZPE | G | % |
|---|---|---|---|
| 2-1 | -998.681458 | -998.732066 | 41.06% |
| 2-2 | -998.680987 | -998.731277 | 17.8% |
| 2-3 | -998.681460 | -998.732068 | 41.1% |

E+ZPE, G: total energy with zero point energy (ZPE) and Gibbs free energy at B3LYP/6-31G+(d) level of theory with PCM solvent model for MeOH. %: Boltzmann distributions, using the relative Gibbs free energies as weighting factors

**Table S15** Cartesian coordinates of the low-energy re-optimized conformers of compound **2** (1*S*,3*S*,5*R*,6*S*,9*S*,14*R*-**2**) calculated at B3LYP/6-31+G(d) level of theory with PCM solvent model for MeOH.

| **Conformer 2-1** | | Standard Orientation (Ångstroms) | | |
|---|---|---|---|---|
| I | Atom | X | Y | Z |
| 1 | C | -2.8643 | -1.04166 | 1.18018 |
| 2 | C | -3.53072 | 0.29667 | 1.39856 |
| 3 | C | -2.52396 | 1.46831 | 1.38414 |
| 4 | C | -1.0846 | 0.97936 | 1.64231 |
| 5 | C | -0.53777 | -0.12361 | 0.63567 |
| 6 | C | -1.39963 | -1.1073 | 1.5227 |
| 7 | C | -3.46246 | -2.10815 | 0.62986 |
| 8 | C | -1.03947 | -0.15565 | 2.71447 |
| 9 | C | 0.98136 | -0.27652 | 0.63025 |
| 10 | C | 1.37851 | 0.69522 | -0.4795 |
| 11 | O | 0.26231 | 0.7916 | -1.38326 |
| 12 | C | -0.8153 | 0.0098 | -0.85847 |
| 13 | O | -0.80037 | -1.29597 | -1.43872 |
| 14 | C | -1.16054 | -1.26602 | -2.81256 |
| 15 | C | 2.56942 | 0.23525 | -1.26157 |
| 16 | C | 3.81707 | 0.74657 | -1.2332 |
| 17 | C | 4.88627 | 0.1869 | -2.1019 |
| 18 | O | 4.50727 | -0.881 | -2.8226 |
| 19 | C | 4.27698 | 1.87972 | -0.36348 |

| | | | | |
|---|---|---|---|---|
| 20 | O | 6.02377 | 0.61827 | -2.18005 |
| 21 | O | -4.16951 | 0.26957 | 2.67322 |
| 22 | H | -4.31297 | 0.48061 | 0.6538 |
| 23 | H | -2.55782 | 1.98034 | 0.41597 |
| 24 | H | -2.79537 | 2.21114 | 2.14409 |
| 25 | H | -0.4034 | 1.81709 | 1.82629 |
| 26 | H | -1.00825 | -2.12503 | 1.62249 |
| 27 | H | -2.92221 | -3.03375 | 0.44987 |
| 28 | H | -4.50596 | -2.08111 | 0.33124 |
| 29 | H | -0.05461 | -0.33746 | 3.16074 |
| 30 | H | -1.7522 | -0.08921 | 3.54164 |
| 31 | H | 1.2541 | -1.30962 | 0.37943 |
| 32 | H | 1.47587 | -0.02531 | 1.57334 |
| 33 | H | 1.51258 | 1.7005 | -0.06963 |
| 34 | H | -1.76526 | 0.50256 | -1.08382 |
| 35 | H | -1.15092 | -2.29192 | -3.19058 |
| 36 | H | -2.16926 | -0.86062 | -2.93757 |
| 37 | H | -0.4421 | -0.67847 | -3.39174 |
| 38 | H | 2.35077 | -0.59969 | -1.92736 |
| 39 | H | 5.31272 | -1.11602 | -3.32684 |
| 40 | H | 4.50331 | 2.75942 | -0.97498 |
| 41 | H | 3.53843 | 2.17346 | 0.3862 |
| 42 | H | 5.18216 | 1.59834 | 0.18615 |
| 43 | H | -4.60396 | -0.59658 | 2.75521 |

| **Conformer 2-2** | | Standard Orientation (Ångstroms) | | |
|---|---|---|---|---|
| I | Atom | X | Y | Z |
| 1 | C | -2.03801 | -1.91195 | 1.76271 |
| 2 | C | -3.15745 | -0.91866 | 1.97015 |
| 3 | C | -2.72323 | 0.53327 | 1.67027 |
| 4 | C | -1.18944 | 0.6848 | 1.71544 |
| 5 | C | -0.37182 | -0.26309 | 0.73481 |
| 6 | C | -0.64265 | -1.35274 | 1.84677 |
| 7 | C | -2.21502 | -3.19792 | 1.42658 |
| 8 | C | -0.5547 | -0.16335 | 2.8633 |
| 9 | C | 1.06529 | 0.18352 | 0.47621 |
| 10 | C | 0.8935 | 1.04688 | -0.77244 |
| 11 | O | -0.27259 | 0.5682 | -1.46702 |
| 12 | C | -0.86287 | -0.48067 | -0.69302 |
| 13 | O | -0.38858 | -1.75068 | -1.14405 |

| | | | | |
|---|---|---|---|---|
| 14 | C | -0.88397 | -2.0705 | -2.43649 |
| 15 | C | 2.05767 | 0.96033 | -1.70915 |
| 16 | C | 2.97602 | 1.91227 | -1.9707 |
| 17 | C | 4.01786 | 1.56479 | -2.9707 |
| 18 | O | 4.80374 | 2.61208 | -3.27157 |
| 19 | C | 3.04569 | 3.26709 | -1.33466 |
| 20 | O | 4.19585 | 0.48121 | -3.49734 |
| 21 | O | -3.56643 | -0.99207 | 3.33436 |
| 22 | H | -4.03299 | -1.16921 | 1.36099 |
| 23 | H | -3.08183 | 0.83527 | 0.67988 |
| 24 | H | -3.17618 | 1.22188 | 2.3939 |
| 25 | H | -0.88976 | 1.73816 | 1.69824 |
| 26 | H | 0.13719 | -2.11142 | 1.96988 |
| 27 | H | -1.37242 | -3.85847 | 1.24014 |
| 28 | H | -3.20782 | -3.62281 | 1.31441 |
| 29 | H | 0.4663 | 0.12103 | 3.14413 |
| 30 | H | -1.1242 | -0.24983 | 3.79323 |
| 31 | H | 1.7006 | -0.68959 | 0.27965 |
| 32 | H | 1.52786 | 0.75031 | 1.28976 |
| 33 | H | 0.66432 | 2.07775 | -0.4874 |
| 34 | H | -1.95111 | -0.43757 | -0.79237 |
| 35 | H | -0.50691 | -3.05922 | -2.7117 |
| 36 | H | -1.97767 | -2.10611 | -2.43189 |
| 37 | H | -0.53197 | -1.34847 | -3.17915 |
| 38 | H | 2.12546 | 0.00484 | -2.23264 |
| 39 | H | 5.42459 | 2.2439 | -3.93398 |
| 40 | H | 4.04408 | 3.44671 | -0.92089 |
| 41 | H | 2.82802 | 4.04669 | -2.07205 |
| 42 | H | 2.34259 | 3.38805 | -0.50703 |
| 43 | H | -3.59699 | -1.93473 | 3.57143 |

| **Conformer 2-3** | | Standard Orientation (Ångstroms) | | |
|---|---|---|---|---|
| I | Atom | X | Y | Z |
| 1 | C | -2.50094 | -1.01324 | 1.72152 |
| 2 | C | -3.18668 | 0.31302 | 1.95297 |
| 3 | C | -2.21475 | 1.50877 | 1.84354 |
| 4 | C | -0.74883 | 1.06266 | 2.01539 |
| 5 | C | -0.24131 | -0.05086 | 0.99982 |
| 6 | C | -1.01532 | -1.03361 | 1.96549 |
| 7 | C | -3.10612 | -2.10799 | 1.23845 |

| | | | | |
|---|---|---|---|---|
| 8 | C | -0.60179 | -0.04391 | 3.10792 |
| 9 | C | 1.2764 | -0.16597 | 0.89213 |
| 10 | C | 1.57358 | 0.80221 | -0.25634 |
| 11 | O | 0.40545 | 0.82326 | -1.08999 |
| 12 | C | -0.61907 | 0.03719 | -0.47438 |
| 13 | O | -0.61874 | -1.28323 | -1.01961 |
| 14 | C | -1.08671 | -1.30112 | -2.36031 |
| 15 | C | 2.80719 | 0.42455 | -1.02877 |
| 16 | C | 3.07952 | 0.62008 | -2.33674 |
| 17 | C | 4.34731 | 0.11574 | -2.92781 |
| 18 | O | 5.18666 | -0.43884 | -2.03618 |
| 19 | C | 2.20587 | 1.36199 | -3.30582 |
| 20 | O | 4.6528 | 0.17896 | -4.10615 |
| 21 | O | -3.73816 | 0.30195 | 3.26801 |
| 22 | H | -4.02145 | 0.45861 | 1.25843 |
| 23 | H | -2.32688 | 1.99552 | 0.86827 |
| 24 | H | -2.45453 | 2.26328 | 2.60263 |
| 25 | H | -0.0796 | 1.92155 | 2.13397 |
| 26 | H | -0.59105 | -2.03839 | 2.06207 |
| 27 | H | -2.55461 | -3.02397 | 1.04398 |
| 28 | H | -4.16766 | -2.11469 | 1.01069 |
| 29 | H | 0.41521 | -0.18973 | 3.49079 |
| 30 | H | -1.25914 | 0.02514 | 3.97955 |
| 31 | H | 1.55625 | -1.19493 | 0.63167 |
| 32 | H | 1.82692 | 0.10814 | 1.79706 |
| 33 | H | 1.70358 | 1.8138 | 0.1468 |
| 34 | H | -1.59101 | 0.50756 | -0.64794 |
| 35 | H | -1.0745 | -2.33603 | -2.71282 |
| 36 | H | -2.11459 | -0.92953 | -2.41394 |
| 37 | H | -0.43644 | -0.708 | -3.00942 |
| 38 | H | 3.55589 | -0.07759 | -0.41668 |
| 39 | H | 5.94911 | -0.71219 | -2.5865 |
| 40 | H | 1.5968 | 0.66055 | -3.88412 |
| 41 | H | 1.54703 | 2.08007 | -2.81002 |
| 42 | H | 2.80682 | 1.95186 | -4.00704 |
| 43 | H | -4.1425 | -0.57265 | 3.39943 |

**Figure S48** DFT-optimized structures for low-energy conformers of **3** (1*S*,5*S*,6*S*,9*S*,14*S*-**3**) at B3LYP/6-31+G(d) level in MeOH (PCM).

Conformer populations were calculated using the Gibbs free energy and Boltzmann population at 298 K estimated thereof.

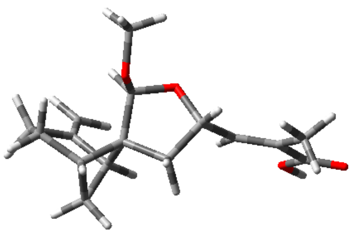

Conf. 3-1 67.8%

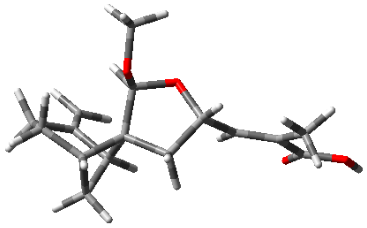

Conf. 3-2 31.0%

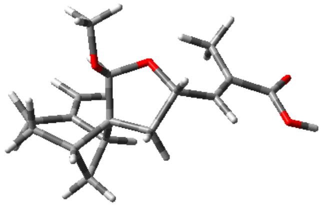

Conf. 3-3 0.6%

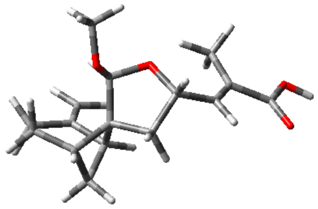

Conf. 3-4 0.3%

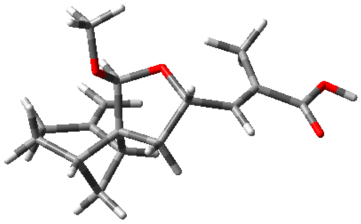

Conf. 3-5 0.3%

**Table S16** Harmonic frequencies (cm**$^{-1}$) of compound **3**.

| **Conformations** | **Frequencies** | | |
|---|---|---|---|
| | 1 | 2 | 3 |
| 3-1 | 18.3149 | 25.0466 | 51.3692 |
| 3-2 | 20.0477 | 26.5908 | 51.5991 |
| 3-3 | 15.1774 | 26.5726 | 63.4862 |
| 3-4 | 16.3926 | 29.8734 | 61.5577 |
| 3-5 | 16.4050 | 29.8643 | 61.5653 |

**Table S17** Important thermodynamic parameters (a.u.) and Boltzmann distributions of the optimized compound **3** at B3LYP/6-31G+(d) level of theory with PCM solvent model for MeOH.

| Conformations | E+ZPE | G | % |
|---|---|---|---|
| 3-1 | -923.467275 | -923.517014 | 67.8% |
| 3-2 | -923.466815 | -923.516276 | 31.0% |
| 3-3 | -923.463038 | -923.512484 | 0.6% |
| 3-4 | -923.462759 | -923.511969 | 0.3% |
| 3-5 | -923.462760 | -923.511969 | 0.3% |

E+ZPE, G: total energy with zero point energy (ZPE) and Gibbs free energy at B3LYP/6-31G+(d) level of theory with PCM solvent model for MeOH. %: Boltzmann distributions, using the relative Gibbs free energies as weighting factors.

**Table S18** Cartesian coordinates of the low-energy re-optimized conformers of compound **3** (1*S*,5*S*,6*S*,9*S*,14*S*-**3**) calculated at B3LYP/6-31+G(d) level of theory with PCM solvent model for MeOH.

| **Conformer 3-1** | | Standard Orientation (Ångstroms) | | |
|---|---|---|---|---|
| I | Atom | X | Y | Z |
| 1 | C | -1.34455 | -0.13987 | -2.35161 |
| 2 | C | -2.41803 | -1.19532 | -2.46456 |
| 3 | C | -2.35301 | -2.2104 | -1.30476 |
| 4 | C | -0.94359 | -2.27269 | -0.68661 |
| 5 | C | -0.43159 | -0.90299 | -0.07601 |
| 6 | C | -0.11644 | -0.54851 | -1.58948 |
| 7 | C | -1.45599 | 1.11007 | -2.81904 |
| 8 | C | 0.17235 | -2.08347 | -1.75752 |
| 9 | C | 0.78446 | -0.93895 | 0.84702 |
| 10 | C | 0.76404 | 0.44985 | 1.485 |
| 11 | O | -0.566 | 0.97401 | 1.34659 |
| 12 | C | -1.38413 | -0.0184 | 0.72139 |
| 13 | O | -2.01661 | -0.82658 | 1.71749 |
| 14 | C | -3.02227 | -0.10571 | 2.41618 |
| 15 | C | 1.69123 | 1.41953 | 0.80977 |
| 16 | C | 2.91616 | 1.79834 | 1.22867 |
| 17 | C | 3.70434 | 2.79902 | 0.46246 |
| 18 | O | 3.13555 | 3.17686 | -0.69438 |
| 19 | C | 3.61681 | 1.28008 | 2.45033 |
| 20 | O | 4.78186 | 3.25485 | 0.80416 |
| 21 | H | -2.26453 | -1.73341 | -3.40885 |

| | | | | |
|---|---|---|---|---|
| 22 | H | -3.41928 | -0.75239 | -2.51269 |
| 23 | H | -2.63929 | -3.20003 | -1.68024 |
| 24 | H | -3.08742 | -1.94827 | -0.53492 |
| 25 | H | -0.82025 | -3.14133 | -0.03162 |
| 26 | H | 0.75292 | 0.09044 | -1.77293 |
| 27 | H | -0.66625 | 1.84431 | -2.68801 |
| 28 | H | -2.34599 | 1.43381 | -3.35057 |
| 29 | H | 1.17231 | -2.40326 | -1.44192 |
| 30 | H | -0.00874 | -2.52246 | -2.74389 |
| 31 | H | 1.7332 | -1.17011 | 0.35288 |
| 32 | H | 0.63231 | -1.69567 | 1.62718 |
| 33 | H | 0.97573 | 0.38297 | 2.55623 |
| 34 | H | -2.14546 | 0.47209 | 0.10803 |
| 35 | H | -3.4758 | -0.77695 | 3.15051 |
| 36 | H | -2.59234 | 0.74805 | 2.94824 |
| 37 | H | -3.80283 | 0.23158 | 1.72736 |
| 38 | H | 1.28434 | 1.86 | -0.09934 |
| 39 | H | 3.77202 | 3.82471 | -1.06046 |
| 40 | H | 3.11011 | 0.42435 | 2.90365 |
| 41 | H | 4.62903 | 0.94444 | 2.19871 |
| 42 | H | 3.69164 | 2.06742 | 3.20756 |

| **Conformer 3-2** | | Standard Orientation (Ångstroms) | | |
|---|---|---|---|---|
| I | Atom | X | Y | Z |
| 1 | C | -0.99891 | 0.61341 | -2.49565 |
| 2 | C | -1.92167 | -0.39538 | -3.1356 |
| 3 | C | -1.90428 | -1.74665 | -2.39159 |
| 4 | C | -0.59673 | -1.93977 | -1.60066 |
| 5 | C | -0.33186 | -0.84251 | -0.4883 |
| 6 | C | 0.1491 | 0.03545 | -1.71864 |
| 7 | C | -1.18188 | 1.93955 | -2.52968 |
| 8 | C | 0.62892 | -1.32493 | -2.34048 |
| 9 | C | 0.74019 | -1.12848 | 0.56095 |
| 10 | C | 0.47343 | -0.05538 | 1.6161 |
| 11 | O | -0.87604 | 0.40283 | 1.44111 |
| 12 | C | -1.48038 | -0.34995 | 0.38596 |
| 13 | O | -2.15206 | -1.49126 | 0.92643 |
| 14 | C | -3.32317 | -1.12005 | 1.6405 |
| 15 | C | 1.36574 | 1.14352 | 1.47281 |
| 16 | C | 2.45323 | 1.4437 | 2.21152 |

| | | | | |
|---|---|---|---|---|
| 17 | C | 3.14673 | 2.71277 | 1.87557 |
| 18 | O | 4.10562 | 3.0195 | 2.76463 |
| 19 | C | 3.02741 | 0.60315 | 3.31078 |
| 20 | O | 2.92268 | 3.43761 | 0.92266 |
| 21 | H | -1.58022 | -0.55947 | -4.16578 |
| 22 | H | -2.94987 | -0.02157 | -3.19951 |
| 23 | H | -2.02175 | -2.55705 | -3.12082 |
| 24 | H | -2.76367 | -1.81365 | -1.71509 |
| 25 | H | -0.46897 | -2.97387 | -1.26436 |
| 26 | H | 0.95856 | 0.74669 | -1.52744 |
| 27 | H | -0.50361 | 2.62558 | -2.02978 |
| 28 | H | -2.01888 | 2.37817 | -3.06476 |
| 29 | H | 1.60473 | -1.67688 | -1.98591 |
| 30 | H | 0.63728 | -1.40147 | -3.43249 |
| 31 | H | 1.76852 | -1.11556 | 0.18666 |
| 32 | H | 0.56802 | -2.11824 | 1.00272 |
| 33 | H | 0.54366 | -0.48163 | 2.62109 |
| 34 | H | -2.19777 | 0.28146 | -0.14612 |
| 35 | H | -3.79685 | -2.03085 | 2.01674 |
| 36 | H | -3.07448 | -0.48248 | 2.49401 |
| 37 | H | -4.03091 | -0.60955 | 0.98017 |
| 38 | H | 1.0576 | 1.83897 | 0.69129 |
| 39 | H | 4.46364 | 3.86724 | 2.42865 |
| 40 | H | 2.56602 | -0.38558 | 3.37609 |
| 41 | H | 4.09758 | 0.43483 | 3.14763 |
| 42 | H | 2.89534 | 1.09778 | 4.27859 |

| **Conformer 3-3** | | Standard Orientation (Ångstroms) | | |
|---|---|---|---|---|
| I | Atom | X | Y | Z |
| 1 | C | -0.47418 | 0.24115 | -2.2207 |
| 2 | C | -1.52927 | -0.65133 | -2.82838 |
| 3 | C | -1.7429 | -1.93849 | -2.00555 |
| 4 | C | -0.50444 | -2.279 | -1.15557 |
| 5 | C | -0.10169 | -1.16537 | -0.10316 |
| 6 | C | 0.5456 | -0.45507 | -1.36562 |
| 7 | C | -0.43974 | 1.57343 | -2.3516 |
| 8 | C | 0.82237 | -1.91133 | -1.88524 |
| 9 | C | 0.877 | -1.54208 | 1.00588 |
| 10 | C | 0.79492 | -0.32781 | 1.93515 |
| 11 | O | -0.50016 | 0.24905 | 1.7495 |

| | | | | |
|---|---|---|---|---|
| 12 | C | -1.1795 | -0.44199 | 0.69688 |
| 13 | O | -2.05681 | -1.42441 | 1.25407 |
| 14 | C | -3.17239 | -0.82613 | 1.89935 |
| 15 | C | 1.89237 | 0.67403 | 1.66323 |
| 16 | C | 1.82956 | 2.01605 | 1.52019 |
| 17 | C | 3.05496 | 2.7923 | 1.18895 |
| 18 | O | 4.18037 | 2.05725 | 1.16606 |
| 19 | C | 0.59264 | 2.84968 | 1.6901 |
| 20 | O | 3.09548 | 3.98627 | 0.94821 |
| 21 | H | -1.19426 | -0.93254 | -3.83512 |
| 22 | H | -2.48389 | -0.12661 | -2.94846 |
| 23 | H | -1.96423 | -2.76651 | -2.68947 |
| 24 | H | -2.62143 | -1.82923 | -1.35996 |
| 25 | H | -0.5495 | -3.29592 | -0.75232 |
| 26 | H | 1.45123 | 0.13309 | -1.18852 |
| 27 | H | 0.32717 | 2.17765 | -1.8754 |
| 28 | H | -1.18061 | 2.09894 | -2.94689 |
| 29 | H | 1.72103 | -2.38598 | -1.47443 |
| 30 | H | 0.84812 | -2.05988 | -2.96956 |
| 31 | H | 1.89348 | -1.76551 | 0.6687 |
| 32 | H | 0.50672 | -2.4291 | 1.53576 |
| 33 | H | 0.88895 | -0.65076 | 2.97813 |
| 34 | H | -1.75745 | 0.27492 | 0.10654 |
| 35 | H | -2.84996 | -0.20022 | 2.73667 |
| 36 | H | -3.75677 | -0.23464 | 1.18789 |
| 37 | H | -3.80921 | -1.62422 | 2.29049 |
| 38 | H | 2.86798 | 0.19626 | 1.57032 |
| 39 | H | 4.87194 | 2.71311 | 0.94036 |
| 40 | H | -0.02734 | 2.48916 | 2.51644 |
| 41 | H | 0.83055 | 3.88939 | 1.93901 |
| 42 | H | 0.0033 | 2.8524 | 0.7681 |

| **Conformer 3-4** | | Standard Orientation (Ångstroms) | | |
|---|---|---|---|---|
| I | Atom | X | Y | Z |
| 1 | C | -0.86732 | -0.34118 | -2.06056 |
| 2 | C | -1.91478 | -1.41346 | -2.23928 |
| 3 | C | -1.83728 | -2.48722 | -1.13436 |
| 4 | C | -0.4346 | -2.54706 | -0.50066 |
| 5 | C | 0.03618 | -1.19889 | 0.18573 |
| 6 | C | 0.36087 | -0.75952 | -1.30391 |

| | | | | |
|---|---|---|---|---|
| 7 | C | -1.00059 | 0.92703 | -2.46953 |
| 8 | C | 0.68861 | -2.27644 | -1.5461 |
| 9 | C | 1.23689 | -1.24794 | 1.12685 |
| 10 | C | 1.20066 | 0.14216 | 1.76828 |
| 11 | O | -0.16113 | 0.57761 | 1.73383 |
| 12 | C | -0.9459 | -0.3784 | 1.01479 |
| 13 | O | -1.59631 | -1.25802 | 1.93598 |
| 14 | C | -2.62885 | -0.59662 | 2.65364 |
| 15 | C | 2.10861 | 1.11975 | 1.06186 |
| 16 | C | 1.87967 | 2.38887 | 0.66194 |
| 17 | C | 2.98988 | 3.04483 | -0.07304 |
| 18 | O | 2.57851 | 4.11769 | -0.7684 |
| 19 | C | 0.64528 | 3.1965 | 0.9233 |
| 20 | O | 4.15361 | 2.68588 | -0.10695 |
| 21 | H | -1.74048 | -1.89726 | -3.209 |
| 22 | H | -2.92548 | -0.99122 | -2.2743 |
| 23 | H | -2.09277 | -3.46231 | -1.56597 |
| 24 | H | -2.58836 | -2.28576 | -0.36246 |
| 25 | H | -0.29745 | -3.4449 | 0.11075 |
| 26 | H | 1.21671 | -0.09249 | -1.44532 |
| 27 | H | -0.22814 | 1.67044 | -2.29432 |
| 28 | H | -1.89065 | 1.25656 | -2.99725 |
| 29 | H | 1.69248 | -2.58803 | -1.23492 |
| 30 | H | 0.52956 | -2.66834 | -2.55584 |
| 31 | H | 2.19138 | -1.48198 | 0.6462 |
| 32 | H | 1.06582 | -2.00807 | 1.89991 |
| 33 | H | 1.52416 | 0.07895 | 2.81333 |
| 34 | H | -1.69578 | 0.1458 | 0.41536 |
| 35 | H | -3.09482 | -1.32102 | 3.32714 |
| 36 | H | -2.22374 | 0.22265 | 3.25491 |
| 37 | H | -3.39393 | -0.22012 | 1.96775 |
| 38 | H | 3.09276 | 0.69612 | 0.85052 |
| 39 | H | 3.39651 | 4.43466 | -1.20397 |
| 40 | H | 0.16609 | 2.9242 | 1.86757 |
| 41 | H | 0.87751 | 4.26289 | 1.01903 |
| 42 | H | -0.07341 | 3.07369 | 0.10745 |

| **Conformer 3-5** | | Standard Orientation (Ångstroms) | | |
|---|---|---|---|---|
| I | Atom | X | Y | Z |
| 1 | C | -0.83666 | 0.19791 | -2.15083 |

| | | | | |
|---|---|---|---|---|
| 2 | C | -1.78428 | -0.86529 | -2.65268 |
| 3 | C | -1.63863 | -2.1903 | -1.87793 |
| 4 | C | -0.25705 | -2.30547 | -1.20949 |
| 5 | C | 0.06048 | -1.1636 | -0.1597 |
| 6 | C | 0.39428 | -0.30763 | -1.45243 |
| 7 | C | -1.05447 | 1.51463 | -2.25962 |
| 8 | C | 0.87142 | -1.66851 | -2.07618 |
| 9 | C | 1.23042 | -1.38717 | 0.79507 |
| 10 | C | 1.02429 | -0.28564 | 1.83659 |
| 11 | O | -0.34325 | 0.11893 | 1.77929 |
| 12 | C | -1.01794 | -0.68002 | 0.80284 |
| 13 | O | -1.60452 | -1.82312 | 1.43042 |
| 14 | C | -2.71236 | -1.46393 | 2.24439 |
| 15 | C | 1.97677 | 0.86752 | 1.64835 |
| 16 | C | 1.76767 | 2.17705 | 1.39494 |
| 17 | C | 2.99335 | 3.01628 | 1.32737 |
| 18 | O | 2.72318 | 4.32861 | 1.42071 |
| 19 | C | 0.45801 | 2.8653 | 1.16307 |
| 20 | O | 4.14087 | 2.62206 | 1.21523 |
| 21 | H | -1.55085 | -1.05285 | -3.70876 |
| 22 | H | -2.82639 | -0.52805 | -2.61495 |
| 23 | H | -1.78826 | -3.02711 | -2.57056 |
| 24 | H | -2.42796 | -2.27166 | -1.12228 |
| 25 | H | -0.05601 | -3.32222 | -0.85633 |
| 26 | H | 1.18605 | 0.44092 | -1.34803 |
| 27 | H | -0.35057 | 2.24669 | -1.87595 |
| 28 | H | -1.94544 | 1.89759 | -2.74855 |
| 29 | H | 1.88898 | -1.97142 | -1.80335 |
| 30 | H | 0.78309 | -1.78037 | -3.16166 |
| 31 | H | 2.21866 | -1.36095 | 0.32574 |
| 32 | H | 1.12777 | -2.36623 | 1.28046 |
| 33 | H | 1.20689 | -0.69288 | 2.83831 |
| 34 | H | -1.79844 | -0.08325 | 0.32176 |
| 35 | H | -3.12433 | -2.3769 | 2.68286 |
| 36 | H | -2.40116 | -0.80078 | 3.05692 |
| 37 | H | -3.49236 | -0.98614 | 1.64369 |
| 38 | H | 3.01227 | 0.5457 | 1.78598 |
| 39 | H | 3.60747 | 4.74748 | 1.37864 |
| 40 | H | -0.31761 | 2.19891 | 0.786 |
| 41 | H | 0.1021 | 3.32812 | 2.08877 |
| 42 | H | 0.55451 | 3.64777 | 0.40186 |

**Figure S49** DFT-optimized structures for low-energy conformers of **4** (1*S*,5*S*,6*S*,9*S*,14*R*-**4**) at B3LYP/6-31+G(d) level in MeOH (PCM).

Conformer populations were calculated using the Gibbs free energy and Boltzmann population at 298 K estimated thereof.

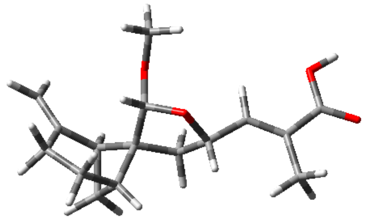

Conf. 4-1 39.9%

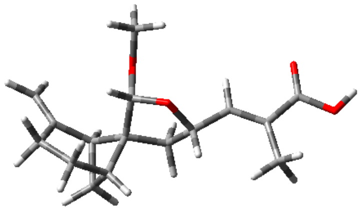

Conf. 4-2 19.6%

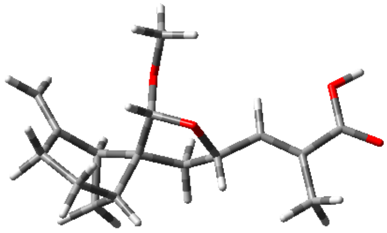

Conf. 4-3 40.5%

**Table S19** Harmonic frequencies (cm**$^{-1}$) of compound **4**.

| **Conformations** | **Frequencies** | | |
|---|---|---|---|
| | 1 | 2 | 3 |
| 4-1 | 26.3742 | 31.1182 | 47.1196 |
| 4-2 | 27.7961 | 31.5383 | 48.0854 |
| 4-3 | 26.2287 | 31.0826 | 47.1166 |

**Table S20** Important thermodynamic parameters (a.u.) and Boltzmann distributions of the optimized compound **4** at B3LYP/6-31G+(d) level of theory with PCM solvent model for MeOH.

| Conformations | E+ZPE | G | % |
|---|---|---|---|
| 4-1 | -923.466817 | -923.515931 | 39.9% |
| 4-2 | -923.466347 | -923.515262 | 19.6% |
| 4-3 | -923.466820 | -923.515944 | 40.5% |

E+ZPE, G: total energy with zero point energy (ZPE) and Gibbs free energy at B3LYP/6-31G+(d) level of theory with PCM solvent model for MeOH. %: Boltzmann distributions, using the relative Gibbs free energies as weighting factors.

**Table S21** Cartesian coordinates of the low-energy re-optimized conformers of compound **4** (1*S*,5*S*,6*S*,9*S*,14*R*-**4**) calculated at B3LYP/6-31+G(d) level of theory with PCM solvent model for MeOH.

| **Conformer 4-1** | | Standard Orientation (Ångstroms) | | |
|---|---|---|---|---|
| I | Atom | X | Y | Z |
| 1 | C | 2.53717 | 0.35688 | -2.22331 |
| 2 | C | 1.89106 | -0.22625 | -3.45632 |
| 3 | C | 0.60704 | -1.01158 | -3.11919 |
| 4 | C | 0.60209 | -1.4794 | -1.6533 |
| 5 | C | 0.7168 | -0.3279 | -0.56274 |
| 6 | C | 2.25092 | -0.36595 | -0.93497 |
| 7 | C | 3.26283 | 1.48219 | -2.20989 |
| 8 | C | 2.03089 | -1.89927 | -1.1798 |
| 9 | C | 0.28385 | -0.74555 | 0.84009 |
| 10 | C | -1.19509 | -0.36134 | 0.82553 |
| 11 | O | -1.35101 | 0.71008 | -0.12339 |
| 12 | C | -0.08088 | 0.96048 | -0.73539 |
| 13 | O | 0.59489 | 2.00871 | -0.03683 |
| 14 | C | -0.01255 | 3.27131 | -0.26949 |
| 15 | C | -1.68764 | 0.1211 | 2.15465 |
| 16 | C | -2.51295 | -0.52082 | 3.00649 |
| 17 | C | -2.93125 | 0.11683 | 4.28298 |
| 18 | O | -2.3608 | 1.31012 | 4.51517 |
| 19 | C | -3.08347 | -1.89357 | 2.80185 |
| 20 | O | -3.71232 | -0.36604 | 5.08484 |
| 21 | H | 2.60943 | -0.91382 | -3.92106 |
| 22 | H | 1.6669 | 0.54584 | -4.2011 |
| 23 | H | 0.53455 | -1.87864 | -3.78675 |
| 24 | H | -0.27864 | -0.39822 | -3.31866 |
| 25 | H | -0.18457 | -2.21828 | -1.46731 |
| 26 | H | 2.9463 | -0.13014 | -0.12295 |
| 27 | H | 3.67431 | 1.88391 | -1.28823 |
| 28 | H | 3.45891 | 2.03777 | -3.12163 |
| 29 | H | 2.06124 | -2.51522 | -0.27349 |
| 30 | H | 2.67425 | -2.38604 | -1.91986 |
| 31 | H | 0.41625 | -1.80735 | 1.0686 |
| 32 | H | 0.84269 | -0.17413 | 1.59224 |
| 33 | H | -1.79479 | -1.18873 | 0.43523 |
| 34 | H | -0.22999 | 1.24623 | -1.78049 |
| 35 | H | 0.55359 | 4.02913 | 0.27884 |

| | | | | |
|---|---|---|---|---|
| 36 | H | 0.01567 | 3.52175 | -1.33438 |
| 37 | H | -1.04431 | 3.28106 | 0.09419 |
| 38 | H | -1.32363 | 1.1157 | 2.41268 |
| 39 | H | -2.73873 | 1.58308 | 5.37585 |
| 40 | H | -2.90067 | -2.51998 | 3.68208 |
| 41 | H | -4.16455 | -1.83444 | 2.63816 |
| 42 | H | -2.64381 | -2.41951 | 1.95105 |

| **Conformer 4-2** | | Standard Orientation (Ångstroms) | | |
|---|---|---|---|---|
| I | Atom | X | Y | Z |
| 1 | C | 1.86241 | 0.4078 | -2.84984 |
| 2 | C | 1.10237 | -0.50427 | -3.78158 |
| 3 | C | 0.12986 | -1.4278 | -3.0197 |
| 4 | C | 0.55431 | -1.60299 | -1.55107 |
| 5 | C | 0.65345 | -0.26425 | -0.69889 |
| 6 | C | 2.03611 | -0.09729 | -1.44308 |
| 7 | C | 2.30685 | 1.62692 | -3.18042 |
| 8 | C | 2.10908 | -1.66343 | -1.40597 |
| 9 | C | 0.64635 | -0.47908 | 0.81244 |
| 10 | C | -0.84592 | -0.37371 | 1.12481 |
| 11 | O | -1.44177 | 0.44792 | 0.10408 |
| 12 | C | -0.42554 | 0.80437 | -0.83961 |
| 13 | O | 0.1416 | 2.06852 | -0.48883 |
| 14 | C | -0.76855 | 3.13488 | -0.71647 |
| 15 | C | -1.12357 | 0.26185 | 2.45132 |
| 16 | C | -1.59744 | -0.33172 | 3.56535 |
| 17 | C | -1.80474 | 0.54889 | 4.74333 |
| 18 | O | -2.40822 | -0.09252 | 5.75802 |
| 19 | C | -1.91261 | -1.78818 | 3.72093 |
| 20 | O | -1.49138 | 1.72113 | 4.84683 |
| 21 | H | 1.83225 | -1.12797 | -4.31363 |
| 22 | H | 0.55442 | 0.05993 | -4.54479 |
| 23 | H | 0.10222 | -2.40428 | -3.5181 |
| 24 | H | -0.8903 | -1.0312 | -3.06902 |
| 25 | H | 0.01059 | -2.42168 | -1.06788 |
| 26 | H | 2.8257 | 0.40841 | -0.87798 |
| 27 | H | 2.814 | 2.26307 | -2.46045 |
| 28 | H | 2.16772 | 2.02619 | -4.18021 |
| 29 | H | 2.47486 | -2.08108 | -0.46072 |
| 30 | H | 2.66223 | -2.15861 | -2.21055 |

| | | | | |
|---|---|---|---|---|
| 31 | H | 1.05516 | -1.43776 | 1.14559 |
| 32 | H | 1.22038 | 0.31416 | 1.30821 |
| 33 | H | -1.31959 | -1.35475 | 1.0266 |
| 34 | H | -0.86108 | 0.85807 | -1.84137 |
| 35 | H | -0.2729 | 4.07095 | -0.44526 |
| 36 | H | -1.04691 | 3.18447 | -1.77362 |
| 37 | H | -1.66202 | 3.02494 | -0.09481 |
| 38 | H | -0.92021 | 1.33421 | 2.47104 |
| 39 | H | -2.48468 | 0.60012 | 6.44666 |
| 40 | H | -1.59114 | -2.38932 | 2.86697 |
| 41 | H | -1.40253 | -2.20035 | 4.59854 |
| 42 | H | -2.99082 | -1.93458 | 3.84314 |

| **Conformer 4-3** | | Standard Orientation (Ångstroms) | | |
|---|---|---|---|---|
| I | Atom | X | Y | Z |
| 1 | C | 2.30179 | -0.62475 | -2.31303 |
| 2 | C | 1.42311 | -1.0398 | -3.46773 |
| 3 | C | 0.03288 | -1.512 | -2.99521 |
| 4 | C | 0.06374 | -1.97197 | -1.52717 |
| 5 | C | 0.54303 | -0.88125 | -0.47401 |
| 6 | C | 1.98463 | -1.2676 | -0.98993 |
| 7 | C | 3.26315 | 0.30419 | -2.38997 |
| 8 | C | 1.39827 | -2.70907 | -1.18419 |
| 9 | C | 0.16357 | -1.19075 | 0.97066 |
| 10 | C | -1.19388 | -0.49015 | 1.08372 |
| 11 | O | -1.17446 | 0.6021 | 0.15271 |
| 12 | C | 0.04778 | 0.55525 | -0.59097 |
| 13 | O | 1.01512 | 1.41792 | 0.0109 |
| 14 | C | 0.70717 | 2.78681 | -0.20664 |
| 15 | C | -1.49437 | -0.03187 | 2.48421 |
| 16 | C | -2.20167 | 1.04393 | 2.89112 |
| 17 | C | -2.33466 | 1.35621 | 4.33884 |
| 18 | O | -1.78333 | 0.43844 | 5.15188 |
| 19 | C | -2.93626 | 1.99379 | 1.99066 |
| 20 | O | -2.88926 | 2.33736 | 4.80321 |
| 21 | H | 1.91717 | -1.8716 | -3.98621 |
| 22 | H | 1.30962 | -0.23398 | -4.20179 |
| 23 | H | -0.2998 | -2.33692 | -3.6367 |
| 24 | H | -0.70475 | -0.71162 | -3.12062 |
| 25 | H | -0.84857 | -2.51249 | -1.25364 |

| | | | | |
|---|---|---|---|---|
| 26 | H | 2.79081 | -1.20001 | -0.25234 |
| 27 | H | 3.8429 | 0.5976 | -1.51935 |
| 28 | H | 3.49155 | 0.80341 | -3.32645 |
| 29 | H | 1.37513 | -3.31906 | -0.27366 |
| 30 | H | 1.8389 | -3.32725 | -1.97302 |
| 31 | H | 0.08439 | -2.25545 | 1.21014 |
| 32 | H | 0.90053 | -0.75034 | 1.65461 |
| 33 | H | -1.98426 | -1.17806 | 0.75994 |
| 34 | H | -0.14107 | 0.86964 | -1.62134 |
| 35 | H | 1.47992 | 3.39202 | 0.27501 |
| 36 | H | 0.70631 | 3.01506 | -1.27688 |
| 37 | H | -0.25934 | 3.04534 | 0.23503 |
| 38 | H | -1.05865 | -0.67878 | 3.24514 |
| 39 | H | -1.95636 | 0.79909 | 6.04566 |
| 40 | H | -2.32666 | 2.88092 | 1.79379 |
| 41 | H | -3.21589 | 1.53524 | 1.03828 |
| 42 | H | -3.87825 | 2.32047 | 2.44533 |

**Figure S50** DFT-optimized structures for low-energy conformers of **5** (1*S*,5*S*,6*S*,9*S*,14*R*-**5**) at B3LYP/tzvp level in MeOH (PCM).

Conformer populations were calculated using the Gibbs free energy and Boltzmann population at 298 K estimated thereof.

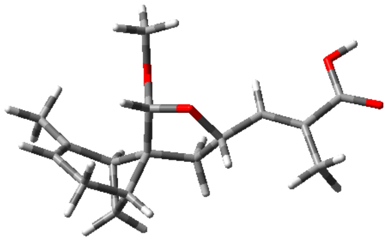

Conf. 5-1 41.3%

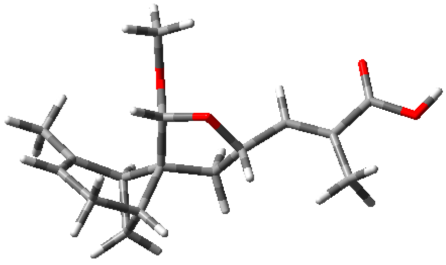

Conf. 5-2 17.1%

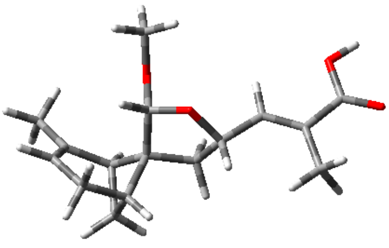

Conf. 5-3 41.4%

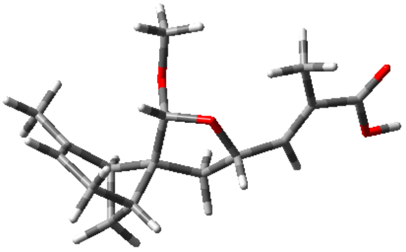

Conf. 5-4 0.1%

**Table S22** Harmonic frequencies (cm**$^{-1}$) of compound **5**.

| **Conformations** | **Frequencies** | | |
|---|---|---|---|
| | 1 | 2 | 3 |
| 5-1 | 20.8988 | 30.2225 | 44.1280 |
| 5-2 | 22.9947 | 30.4718 | 45.9334 |
| 5-3 | 20.8697 | 30.2121 | 44.1263 |
| 5-4 | 25.1160 | 31.5041 | 47.8987 |

**Table S23** Important thermodynamic parameters (a.u.) and Boltzmann distributions of the optimized compound **5** at B3LYP/tzvp level of theory with PCM solvent model for MeOH.

| Conformations | E+ZPE | G | % |
|---|---|---|---|
| 5-1 | -923.764225 | -923.813918 | 41.3% |
| 5-2 | -923.763721 | -923.813088 | 17.1% |
| 5-3 | -923.764226 | -923.813921 | 41.4% |
| 5-4 | -923.759765 | -923.808588 | 0.1% |

E+ZPE, G: total energy with zero point energy (ZPE) and Gibbs free energy at B3LYP/tzvp level of theory with PCM solvent model for MeOH. %: Boltzmann distributions, using the relative Gibbs free energies as weighting factors.

**Table S24** Cartesian coordinates of the low-energy re-optimized conformers of compound **5** (1*S*,5*S*,6*S*,9*S*,14*R*-**5**) calculated at B3LYP/tzvp level of theory with PCM solvent model for MeOH.

| **Conformer 5-1** | | Standard Orientation (Ångstroms) | | |
|---|---|---|---|---|
| I | Atom | X | Y | Z |
| 1 | C | 1.24502 | -2.14306 | -2.29182 |
| 2 | C | 0.05138 | -2.71538 | -2.54501 |
| 3 | C | -0.97912 | -2.87955 | -1.44985 |
| 4 | C | -0.42364 | -2.28365 | -0.1413 |
| 5 | C | 0.26714 | -0.8573 | -0.28129 |
| 6 | C | 1.47201 | -1.7019 | -0.86831 |
| 7 | C | 2.32346 | -1.91537 | -3.29718 |

| | | | | |
|---|---|---|---|---|
| 8 | C | 1.02184 | -2.81695 | 0.1396 |
| 9 | C | 0.47028 | -0.10711 | 1.03129 |
| 10 | C | -0.81299 | 0.71572 | 1.11038 |
| 11 | O | -1.2607 | 0.94048 | -0.23792 |
| 12 | C | -0.40318 | 0.21799 | -1.13013 |
| 13 | O | 0.61191 | 1.0859 | -1.63932 |
| 14 | C | 0.08141 | 2.03386 | -2.55435 |
| 15 | C | -0.6136 | 2.04938 | 1.76078 |
| 16 | C | -1.03469 | 2.43818 | 2.98159 |
| 17 | C | -0.77732 | 3.81928 | 3.46876 |
| 18 | O | -0.04456 | 4.57082 | 2.63121 |
| 19 | C | -1.77053 | 1.57773 | 3.96645 |
| 20 | O | -1.16725 | 4.2718 | 4.53148 |
| 21 | H | -0.2029 | -3.06698 | -3.5409 |
| 22 | H | -1.20334 | -3.94318 | -1.31437 |
| 23 | H | -1.90804 | -2.37417 | -1.73343 |
| 24 | H | -1.1252 | -2.39212 | 0.69216 |
| 25 | H | 2.4712 | -1.29201 | -0.6929 |
| 26 | H | 2.03395 | -2.27187 | -4.29095 |
| 27 | H | 2.55002 | -0.8473 | -3.37514 |
| 28 | H | 3.23572 | -2.44312 | -3.00186 |
| 29 | H | 1.38257 | -2.68286 | 1.16565 |
| 30 | H | 1.22616 | -3.85518 | -0.14301 |
| 31 | H | 0.58982 | -0.74213 | 1.91413 |
| 32 | H | 1.3577 | 0.53507 | 0.96291 |
| 33 | H | -1.60474 | 0.1329 | 1.59016 |
| 34 | H | -1.00169 | -0.17999 | -1.95427 |
| 35 | H | 0.90396 | 2.65654 | -2.91651 |
| 36 | H | -0.37322 | 1.52847 | -3.41191 |
| 37 | H | -0.65173 | 2.68146 | -2.06446 |
| 38 | H | -0.076 | 2.75865 | 1.1313 |
| 39 | H | 0.02749 | 5.42988 | 3.09482 |
| 40 | H | -1.27989 | 1.61012 | 4.94566 |
| 41 | H | -2.80021 | 1.93126 | 4.08388 |
| 42 | H | -1.8085 | 0.52568 | 3.67397 |

| **Conformer 5-2** | | Standard Orientation (Ångstroms) | | |
|---|---|---|---|---|
| I | Atom | X | Y | Z |
| 1 | C | -1.31836 | -2.36064 | -2.08033 |
| 2 | C | -2.5616 | -2.23619 | -1.57508 |

| | | | | |
|---|---|---|---|---|
| 3 | C | -2.77703 | -1.91803 | -0.11202 |
| 4 | C | -1.40716 | -1.75907 | 0.57657 |
| 5 | C | -0.34731 | -0.87206 | -0.21146 |
| 6 | C | -0.18973 | -2.1752 | -1.09832 |
| 7 | C | -0.99155 | -2.65255 | -3.50639 |
| 8 | C | -0.46977 | -2.96445 | 0.22855 |
| 9 | C | 0.84439 | -0.40225 | 0.61712 |
| 10 | C | 0.34822 | 0.95114 | 1.11986 |
| 11 | O | -0.60067 | 1.44372 | 0.15799 |
| 12 | C | -0.79527 | 0.44033 | -0.84625 |
| 13 | O | 0.04609 | 0.70463 | -1.9708 |
| 14 | C | -0.38435 | 1.85001 | -2.69204 |
| 15 | C | 1.44226 | 1.9641 | 1.25104 |
| 16 | C | 1.96773 | 2.46532 | 2.38708 |
| 17 | C | 3.03518 | 3.48526 | 2.22493 |
| 18 | O | 3.39694 | 4.03366 | 3.39692 |
| 19 | C | 1.58497 | 2.07395 | 3.78176 |
| 20 | O | 3.56474 | 3.83353 | 1.18507 |
| 21 | H | -3.43834 | -2.35432 | -2.2055 |
| 22 | H | -3.34414 | -2.72808 | 0.35932 |
| 23 | H | -3.36133 | -0.99742 | -0.01298 |
| 24 | H | -1.50494 | -1.54464 | 1.64571 |
| 25 | H | 0.8 | -2.33692 | -1.53586 |
| 26 | H | -1.89443 | -2.75726 | -4.1166 |
| 27 | H | -0.38985 | -1.84277 | -3.9311 |
| 28 | H | -0.42272 | -3.58451 | -3.58269 |
| 29 | H | 0.40064 | -3.08875 | 0.88264 |
| 30 | H | -0.94632 | -3.94589 | 0.1327 |
| 31 | H | 1.11793 | -1.05857 | 1.4486 |
| 32 | H | 1.72852 | -0.29508 | -0.02428 |
| 33 | H | -0.21369 | 0.82275 | 2.04958 |
| 34 | H | -1.84395 | 0.4458 | -1.15612 |
| 35 | H | 0.28568 | 1.98845 | -3.5449 |
| 36 | H | -1.4011 | 1.70706 | -3.07058 |
| 37 | H | -0.33601 | 2.74623 | -2.06649 |
| 38 | H | 1.81907 | 2.32447 | 0.29195 |
| 39 | H | 4.08687 | 4.6794 | 3.13837 |
| 40 | H | 0.91068 | 1.21477 | 3.81534 |
| 41 | H | 2.47239 | 1.79312 | 4.35958 |
| 42 | H | 1.08733 | 2.90692 | 4.28912 |

| Conformer 5-3 | | Standard Orientation (Ångstroms) | | |
|---|---|---|---|---|
| I | Atom | X | Y | Z |
| 1 | C | 0.85446 | -2.63931 | -1.8013 |
| 2 | C | -0.42389 | -2.98431 | -2.05271 |
| 3 | C | -1.47755 | -2.90251 | -0.9705 |
| 4 | C | -0.83175 | -2.36707 | 0.32263 |
| 5 | C | 0.11984 | -1.10536 | 0.1375 |
| 6 | C | 1.14731 | -2.18837 | -0.39288 |
| 7 | C | 1.96653 | -2.66428 | -2.79545 |
| 8 | C | 0.48257 | -3.15277 | 0.65103 |
| 9 | C | 0.44804 | -0.34896 | 1.41962 |
| 10 | C | -0.66852 | 0.69658 | 1.44858 |
| 11 | O | -1.01967 | 0.96053 | 0.08395 |
| 12 | C | -0.32265 | 0.04052 | -0.76475 |
| 13 | O | 0.84458 | 0.66404 | -1.30366 |
| 14 | C | 0.51651 | 1.62923 | -2.292 |
| 15 | C | -0.26925 | 1.94549 | 2.1852 |
| 16 | C | -0.61351 | 3.22776 | 1.93904 |
| 17 | C | -0.05307 | 4.33047 | 2.76473 |
| 18 | O | 0.71454 | 3.91568 | 3.78756 |
| 19 | C | -1.5788 | 3.68331 | 0.88391 |
| 20 | O | -0.24428 | 5.52025 | 2.58114 |
| 21 | H | -0.73054 | -3.32363 | -3.03805 |
| 22 | H | -1.90155 | -3.89739 | -0.79617 |
| 23 | H | -2.29042 | -2.24273 | -1.29088 |
| 24 | H | -1.54942 | -2.30449 | 1.147 |
| 25 | H | 2.20453 | -1.9688 | -0.21618 |
| 26 | H | 1.62421 | -3.00143 | -3.77915 |
| 27 | H | 2.39359 | -1.6632 | -2.91219 |
| 28 | H | 2.75827 | -3.34285 | -2.46283 |
| 29 | H | 0.85182 | -3.0455 | 1.67719 |
| 30 | H | 0.48796 | -4.22213 | 0.41447 |
| 31 | H | 0.44869 | -0.9577 | 2.32869 |
| 32 | H | 1.43535 | 0.12293 | 1.33333 |
| 33 | H | -1.55369 | 0.26815 | 1.93417 |
| 34 | H | -0.98878 | -0.26961 | -1.57471 |
| 35 | H | 1.44632 | 2.06967 | -2.662 |
| 36 | H | -0.00093 | 1.1572 | -3.13282 |
| 37 | H | -0.10005 | 2.42777 | -1.86982 |
| 38 | H | 0.39752 | 1.74469 | 3.02349 |

| | | | | |
|---|---|---|---|---|
| 39 | H | 0.99525 | 4.75342 | 4.20981 |
| 40 | H | -1.04139 | 3.97864 | -0.02233 |
| 41 | H | -2.31256 | 2.91366 | 0.62953 |
| 42 | H | -2.16564 | 4.54242 | 1.22781 |

| **Conformer 5-4** | | Standard Orientation (Ångstroms) | | |
|---|---|---|---|---|
| I | Atom | X | Y | Z |
| 1 | C | -2.33994 | -2.19477 | -0.19407 |
| 2 | C | -3.26322 | -1.48761 | 0.48682 |
| 3 | C | -2.88164 | -0.69368 | 1.71579 |
| 4 | C | -1.36541 | -0.83404 | 1.95546 |
| 5 | C | -0.44662 | -0.66354 | 0.66457 |
| 6 | C | -0.93571 | -2.13982 | 0.35299 |
| 7 | C | -2.6028 | -2.99862 | -1.42315 |
| 8 | C | -0.93648 | -2.33938 | 1.90868 |
| 9 | C | 1.0312 | -0.42576 | 0.95838 |
| 10 | C | 1.06458 | 1.09544 | 1.00369 |
| 11 | O | 0.13964 | 1.55434 | 0.01324 |
| 12 | C | -0.74364 | 0.46879 | -0.32029 |
| 13 | O | -0.44941 | 0.00786 | -1.63935 |
| 14 | C | -1.0095 | 0.85969 | -2.62827 |
| 15 | C | 2.4458 | 1.66318 | 0.84713 |
| 16 | C | 3.03857 | 2.21575 | -0.23247 |
| 17 | C | 4.41271 | 2.77497 | -0.13249 |
| 18 | O | 5.00079 | 2.57306 | 1.05998 |
| 19 | C | 2.42467 | 2.29839 | -1.59784 |
| 20 | O | 5.00629 | 3.36722 | -1.01691 |
| 21 | H | -4.30184 | -1.46805 | 0.16912 |
| 22 | H | -3.43655 | -1.07035 | 2.58195 |
| 23 | H | -3.14973 | 0.35885 | 1.57851 |
| 24 | H | -1.03843 | -0.29066 | 2.84787 |
| 25 | H | -0.22899 | -2.76907 | -0.19637 |
| 26 | H | -3.6546 | -2.94975 | -1.72289 |
| 27 | H | -1.99761 | -2.62774 | -2.25636 |
| 28 | H | -2.34945 | -4.0494 | -1.25103 |
| 29 | H | 0.04324 | -2.56476 | 2.34469 |
| 30 | H | -1.64736 | -3.06581 | 2.31677 |
| 31 | H | 1.3955 | -0.86715 | 1.89045 |
| 32 | H | 1.6437 | -0.8125 | 0.1341 |

| | | | | |
|---|---|---|---|---|
| 33 | H | 0.66661 | 1.44533 | 1.96513 |
| 34 | H | -1.77536 | 0.82785 | -0.26333 |
| 35 | H | -0.64024 | 1.88371 | -2.51933 |
| 36 | H | -0.71173 | 0.48527 | -3.61134 |
| 37 | H | -2.10216 | 0.84519 | -2.5692 |
| 38 | H | 3.00694 | 1.62341 | 1.78064 |
| 39 | H | 5.87756 | 2.99495 | 0.9488 |
| 40 | H | 1.87474 | 1.3856 | -1.84016 |
| 41 | H | 1.7519 | 3.15881 | -1.6651 |
| 42 | H | 3.18055 | 2.40097 | -2.38356 |
